# Cholinergic System and Its Therapeutic Importance in Inflammation and Autoimmunity

**DOI:** 10.3389/fimmu.2021.660342

**Published:** 2021-04-15

**Authors:** Namrita Halder, Girdhari Lal

**Affiliations:** Laboratory of Autoimmunity and Tolerance, National Centre for Cell Science, Ganeshkhind, Pune, India

**Keywords:** choline acetyltransferase (ChAT), cholinergic system (CS), muscarinic acetylcholine receptors (mAChR), neuroimmunology, neurotransmitters, nicotinic acetylcholine receptors (nAChR)

## Abstract

Neurological and immunological signals constitute an extensive regulatory network in our body that maintains physiology and homeostasis. The cholinergic system plays a significant role in neuroimmune communication, transmitting information regarding the peripheral immune status to the central nervous system (CNS) and vice versa. The cholinergic system includes the neurotransmitter\ molecule, acetylcholine (ACh), cholinergic receptors (AChRs), choline acetyltransferase (ChAT) enzyme, and acetylcholinesterase (AChE) enzyme. These molecules are involved in regulating immune response and playing a crucial role in maintaining homeostasis. Most innate and adaptive immune cells respond to neuronal inputs by releasing or expressing these molecules on their surfaces. Dysregulation of this neuroimmune communication may lead to several inflammatory and autoimmune diseases. Several agonists, antagonists, and inhibitors have been developed to target the cholinergic system to control inflammation in different tissues. This review discusses how various molecules of the neuronal and non-neuronal cholinergic system (NNCS) interact with the immune cells. What are the agonists and antagonists that alter the cholinergic system, and how are these molecules modulate inflammation and immunity. Understanding the various functions of pharmacological molecules could help in designing better strategies to control inflammation and autoimmunity.

## Introduction

The complex bi-directional neuroimmune communication maintains each organ’s physiological balance and functions in the body. The central and peripheral neuronal circuits, immune cells and cytokines, neuro-endocrine hormonal systems, gut microbiota and their metabolites, and the blood-brain and intestinal mucosal barriers are important players throughout this regulatory network. Any disturbance in these systems alters the delicate balance between health and disease (1). The physiological mechanism of cross-talk within the neural network and reticuloendothelial system that regulates immune response, metabolism, and a vast array of pivotal functions constitute the inflammatory reflex (IR). The parasympathetic afferent and efferent arms of the Vagus nerve (VN) serve as a control center that connects impulses between the brain and internal organs (2). The afferent fibers of the VN have innervation in the reticuloendothelial system and major organs of the body. It is activated by low cytokines or endotoxins present in the tissues and communicates *via* neuronal signals sent to the poor cytokine milieu of the central nervous system (CNS) (3).

ACh has also been detected in cells of non-neural origins and microbes. It is vastly found in cardiomyocytes, the entire gastrointestinal (GI) tract, bladder urothelial cells, and various human leukemic cells, demonstrating its diverse function within an organism. The non-neuronal cholinergic system (NNCS) is made up of neurotransmitter acetylcholine, its synthesizing and degrading enzymes, transporters, and receptors within epithelial cells in airways, intestine, skin, urothelium, vagina, placenta, cornea, granulosa cells, endothelial cells, immune cells and mesenchymal cells (4). Signal transduction in keratinocytes, lymphocyte proliferation and differentiation, regulation of cytoskeleton of epithelial cells, differentiation and migration of cells in the epidermis for wound healing, ciliary activities, and regulation of the permeability in the epithelial lining of airways in an autocrine/paracrine manner (5). The details of neuronal origin cholinergic systems, their components, and signaling in the tissues have been discussed earlier (6, 7).

In this review, several immune cells that express components of NNCS and respond to neurotransmitters, specific agonists, and antagonists and their contribution to inflammation and autoimmunity are discussed. We further explored the different cholinergic agonists, antagonists, and AChE inhibitors (AChEI) that modulate the immune system and their effect on the differentiation and function of various immune cells.

## Components of the Non-Neuronal Cholinergic System (NNCS) in Immunity

### Acetylcholine (ACh)

**Figure 1:**
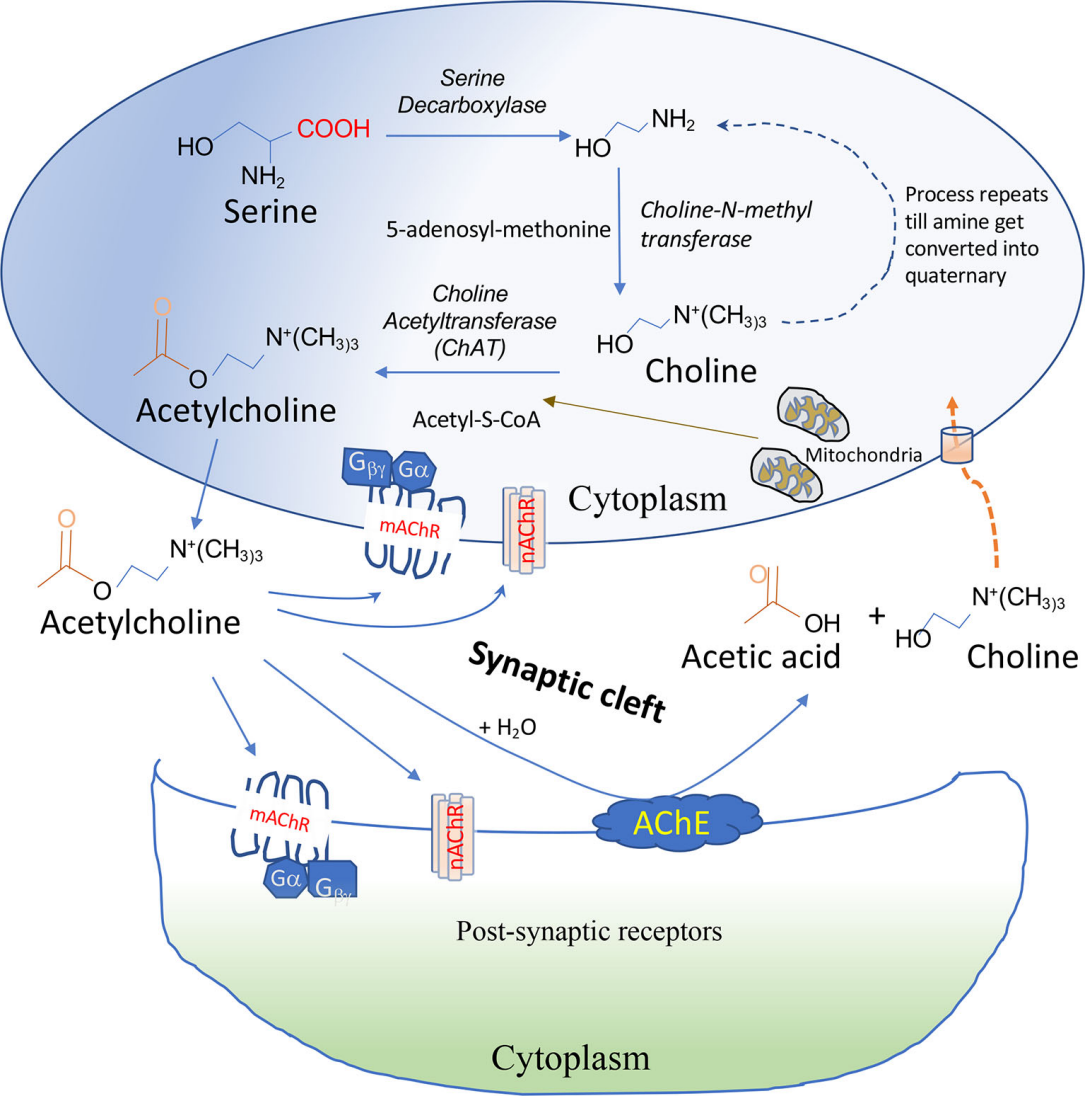
Synthesis and degradation of ACh. ACh is synthesized from Acetyl-S-CoA and choline by the choline acetyltransferase (ChAT) enzyme in the cytoplasm. ACh is secreted out immediately after synthesis in non-immune cells but stored in a specialized vesicle in neuronal cells secreted at presynaptic neurons after activation. Release of ACh requires an influx of Ca2+ ion in the cells followed by docking of ACh-containing vesicle docking at membrane and fusion and release of neurotransmitter into the synaptic cleft *via* a process known as exocytosis. ACh binds *via* the autocrine or paracrine mechanism to nicotinic acetylcholine receptors (nAChRs) or muscarinic acetylcholine receptors (mAChRs) on post-synaptic neurons or immune cells. Acetylcholinesterase (AChE) present on the membrane can degrade ACh into choline and acetic acid. Extracellular choline formed is transported into the cells by choline transporters.

The cholinergic system, which is found in both neuronal and non-neuronal cells, forms a network that performs various complex functions in the body. The ChAT enzyme synthesizes ACh from the precursor molecules, choline (8). The majority of choline is formed by the degradation of lipid, especially lecithin, and hydrolysis of acetylcholine (9). Acetyl-coenzyme A (Acetyl-CoA), produced by mitochondria, is used for the esterification of choline by the cytoplasmic enzyme ChAT in the parasympathetic nervous system and motor neurons **(** **Figure 1** **)**. In addition to the VN, T cells, B cells, dendritic cells (DCs), and macrophages in the follicular and marginal zones of the spleen are other major sources of ACh (10). Immune cells have the machinery to synthesize ACh and directly release it into the bloodstream. In contrast, neuronal cells store ACh after synthesis in a specialized neurosecretory vesicle and release it *via* exocytosis at specialized synaptic clefts (11).

The relative concentration of ACh in humans is found to be 8.66 ± 1.02 pmol/ml in the blood and 3.12 ± 0.36 pmol/ml in plasma (12). Ach is also produced by gut microbes like *Lactobacillus plantarum* (13). Physiological levels of ACh present in the bloodstream affect immune cells in the lymphoid tissues and those that are migrating to the site of inflammation in an autocrine and paracrine manner. Recent studies have correlated lower levels of ACh in chronic inflammatory neurodegenerative diseases like Alzheimer’s disease (AD), vascular dementia (VD), and multiple sclerosis (MS) (14–16). The elevated ACh level is also linked to inflammatory diseases like atopic dermatitis, chronic obstructive pulmonary disease (COPD), and periodontal disease (17–19). Patients with acute ischemic stroke had higher levels of lymphocyte-derived-ACh, which was linked to an increase in post-stroke infection and mortality (20). The diverse ways in which ACh binds to and activates different types of receptors on the surface of various cells and tissues explain its differential outcome and functions within an organism.

### Choline Acetyltransferase (ChAT) Enzyme

ChAT is responsible for the biosynthesis of ACh. The ACh content in cells is proportional to the expression of ChAT within the cells (21). This enzyme is synthesized in the perikaryon of cholinergic neurons and is under the control of multiple regulatory elements (22). The enzyme occurs in both soluble and membrane-bound forms and is transcribed from various ChAT mRNA species that share identical coding regions but differ in the 5’-noncoding regions (23). R, N0, N1, N2, and M-types are some of the ChAT mRNA species that have been identified (24). N1 and N2 type mRNA transcript of ChAT is expressed by T cells, thus differing from the R type in CNS (25). In a murine model, ChAT mRNA is constitutively expressed in T and B cells and mononuclear lymphocytes isolated from the renal vasculature (21). Upon immunological activation, peritoneal macrophages and bone marrow-derived DCs increase ChAT transcription compared to cells in the resting stages (26). ChAT mRNA expression is also detected in human leukemic T cell lines, human peripheral blood T cell and B cells, human lung and alveolar macrophages, and monocytes (27–29). COPD patients’ neutrophils were observed to have over-expression of ChAT. In contrast, epithelial cells of ulcerative colitis patients displayed downregulation of ChAT, indicating, ChAT has differential involvement in different diseases affecting epithelial linings and smooth muscles (30, 31). Several natural and synthetic compounds have been identified as having ChAT stimulatory or inhibitory functions, consequently affecting the immune cells. The summary of the effect of ChAT activators and inhibitors is listed in **Table 1**.

**Table 1:** Effect of ChAT activators and inhibitors on the immune system.

Molecules	Cholinergic effects	Effect of immune status	Experimental model
Estradiol	Increases ChAT activity in the forebrain (32, 33).	CD4^+^ T cells, B cells, and macrophages express estrogen receptors (34). Regulates innate immunity, antigen presentation, and adaptive immune response and has a protective anti-inflammatory effect (35).	1. Ovariectomized RA mice (36). 2. Cancer model (37) 3. Autoimmune disease (38)
Trimethyltin (TMT)	TMT increases ChAT activity in the dentate gyrus (39).	TMT treatment causes atrophy of the thymus, spleen, and lymph nodes. Show reduced antibody levels, lymphocyte proliferation, NK cell function, and peritoneal macrophages’ phagocytic activity (40, 41). Induce microglial/astroglial activation (42).	1.TMT-induced neurotoxic and seizure model (43). 2. Autophagy-induced Alzheimer and epilepsy (44)
A23187	Affect calcium ionophore and increases ChAT expression in leukemic T cells.	A23187 induces the expression of IL-2 receptors in purified T cells (45). It stimulates the proliferation of allogeneic T cells and increases DC-stimulated cytotoxic T lymphocytes (46). A23187 treatment in macrophages causes leukotriene C4 release and enhanced macrophage anti-tumor activity (47).	
Anti-thymocyte globulin (ATG)-Fresenius	Upregulate ChAT expression mediated by CD11a and ACh release through transient increases in intracellular Ca^2+^ (48).	ATG induced a semi-mature phenotype DC with a tolerogenic phenotype that actively suppressed the T cell proliferation (49). Negatively influence B-cell immune reconstitution and deplete cytotoxic T cells (50).	Solid-organ transplantation and allogeneic stem cell transplantation in human (51).
Dibutyryl cAMP	PKA activator upregulates ChAT mRNA expression and ChAT activity and ACh production in the human leukemic cell (52). Dibutyryl cAMP treatment on adipocytes induced Chrna2 expression that controls whole-body metabolism (53).	Dibutyryl-cAMP induces the endogenous production of cAMP and mimics the inhibitory effect of epinephrin on cytotoxic T lymphocytes (54). Cyclic AMP suppress the production of IL-2 in T cells but stimulate antigen-specific and polyclonal antibody production in B cells (55).	–
Phorbol 12-myristate 13-acetate (PMA)	Nonspecific PKC activator. It promotes the expression of both M_3_/M_5_ mAChR and ChAT mRNA in endothelial cells and spinal cord neurons. Thus, activating cholinergic signaling (56, 57).		
Phytohemagglutinin (PHA)	Antigen-induced T cell activation via TCR/CD3ε complexes enhances upregulation of ChAT and M_5_ mAChR expression (58).	PHA-activated lymphocytes respond to cholinergic stimulation with an increase in their free cytoplasmic Ca^2+^ levels.	
Naphthyl-vinyl-pyridine derivatives (NVP)	NVP’s method of ACh antagonism involves inhibiting the enzyme.	LPS challenged Splenic Lymphocyte-derived ACh was prevented by cotreatment with NVP (59).	
α-NETA	α-NETA exhibits a potent inhibitory activity of ChAT	α-NETA treatment significantly delays the onset of EAE. It antagonizes Chemokine-like receptor-1 (CMKLR1) and inhibits β-arrestin-2 cell migration (60).	
Bromoacetylcholine and Bromoacetylcarnitine	Inhibits ChAT and carnitine acetyltransferase (CarAT) activity to synthesize ACh	Synthesis of ACh was reduced by 50 percent in various leukemic T cell lines upon inhibition of ACh synthesizing enzymes (61).	
FK-506 (tacrolimus)	Reduces PHA-induced expression of ChAT mRNA and ACh synthesis through the calcineurin-mediated pathway		Treatment of MG (62).

### Cholinesterase (ChE) and Cholinesterase Inhibitors (ChEI)

The degradation of ACh into choline and acetate ions is regulated by acetylcholinesterase (AChE; EC 3.1.1.7) and butyrylcholinesterase (BChE; EC 3.1.1.7) enzymes, as shown in **Figure 1**. AChE is a 537-amino-acid protein that functions as a primary serine hydrolase. It has a recovery time of around 100 microseconds and can hydrolyze 6 X 10^5^ ACh molecules per minute (63). BChE is a nonspecific serine hydrolase capable of hydrolyzing broad choline-based esters, thus serving as a co-regulator of cholinergic transmission (64). With a half-life of 20 to 60 days, AChE is predominantly found in the neuromuscular junction (NMJ), plasma, liver, and erythrocytes, while BChE is primarily found in the liver and blood plasma, with a reduced half-life of 10 to 14 days in these tissues (65, 66). The cholinergic system-specific catalytic activity of AChE/BChE degrades signal transmission by ACh and determines one’s cholinergic status (CS) (67). Ubiquitous expression of AChE is found within mouse lymphocytes, DCs, and macrophages (68).

In two independent studies, serum AChE levels and CS were substantially higher in patients with irritable bowel syndrome (IBS), whereas CS was significantly lower in IBD patients (69, 70). AChE immunoreactivity was also higher in cirrhotic livers, suggesting a connection between CS dysregulation and GI diseases (71). The possible link of reduced AChE and BChE enzyme activity to proinflammatory processes through hydrolysis of ACh was evident in diseases like MS and AD (72, 73). ChE activity, in turn, can be modulated by ChE inhibitors (AChEIs and BChEIs), thereby increasing ACh levels in the body. The pharmacokinetic properties of ChEIs are thus exploited for the treatment of neurodegenerative and inflammatory diseases like myasthenia gravis (MG) and AD (74). Some ChEIs, such as donepezil, galantamine, and rivastigmine, are currently being used to treat AD (75, 76). Some of the synthetic molecules that enhance or inhibit ChE and affect cholinergic transmission are listed in **Table 2**. While many of these molecules have been studied in the context of neurological diseases, how they modulate inflammation and autoimmunity is still under investigation.

**Table 2:** Modulators of the AChE enzyme.

Molecules	Cholinergic effect	Effect on immune status	Applications
GAL (Galantamine)	Weak competitive and reversible ChEI also allosterically modulates nicotinic acetylcholine receptors.	Treg suppressive activity is enhanced post GAL incubation (77). GAL sensitizes microglial α7-nAChRs and induces Ca^2+^ influx signaling cascades that stimulate Aβ phagocytosis in the AD model (78) GAL resulted in reduced mucosal inflammation associated with decrease MHC II levels and proinflammatory cytokine secretion by splenic CD11c⁺ cells (79).	Improves cognitive function in AD and dementia (80).
Rivastigmine	Rivastigmine inhibits both AChE and BChE in CNS. It preferentially inhibits the G1 enzymatic form of AChE, predominantly found in AD patients.	Rivastigmine significantly decreases nitric oxide release, IL-1β, IL-6, and TNF-α from stimulated macrophages (81). In the EAE model, it reduces microglial activation, encephalitogenic T cells proliferation, and TNF-α and IFN-γ production (82).	Used in improving functional and clinical symptoms of AD and Parkinson’s (83, 84)
Hup A	A highly selective, centrally-acting AChE inhibitor also antagonizes NMDA receptors.	HupA administration showed a reduction of proinflammatory cytokines TNF-α and IL-1β in sepsis-associated encephalopathy. Increased expressions of ChAT and CHRM1 attributed to reduced neuronal apoptosis and septic symptoms relief (85, 86). HupA reduces proinflammatory cytokines (IFN-*γ* and IL-17) and chemokines in the EAE while increases anti-inflammatory cytokines (IL-4 and IL-10 (4).	Hup A is administered for the treatment of AD and schizophrenia (86, 87)
Neostigmine	Blocks the active site of AChE and has limited ability to pass the blood-brain barrier.	Neostigmine increases HLA-DR expression and stimulates TNF-α production in resting DCs. It significantly reduced TNF-α and IL-12p70 production and prevented up-regulation of HLA-DR expression triggered by LPS (26).	It is administered for neurophysiological modifications in MG. It is also used to treat acute colonic pseudo-obstruction, Ogilvie syndrome, and GI disorders (88, 89).
Pyridostigmine (PY)	A potent carbamate peripheral inhibitor of AChE increases the transmission of impulses from cholinergic neurons across the synaptic cleft.	PY enhances anti-inflammatory response in HIV-1-infected patients by reducing T cell proliferation and IFN-γ production and increases IL-4 and IL-10 expression (90). It has a pro-eosinophilic effect through downregulation of IL-5, IL-13, and eotaxin in DSS-induced colitis. It also attenuates DSS-induced microbiota dysbiosis and improves epithelial integrity (91). Cholinergic modulation with PY induces greater recruitment of M2 macrophages and circulatory Treg cells soon after myocardial infarction in rats (92).	PY is used for the management of MG (93). Oral PY to be helpful in different GI dysmotility (94).
Physostigmine	Interfere with acetylcholine signaling such as atropine, scopolamine.	Physostigmine significantly decreases the expression of IL-1β, TNF-α, and IL-10 in the spleen and plasma in mice models, along with reduced neurodegeneration in the hippocampus (95).	ChEI was first investigated for the treatment of AD however discontinued for multiple adverse effects (96).
Ambenonium chloride	AChE inhibitor and down-regulates α6β2 -nAChR mediated dopamine release.		Reduce the aggregation of the β-amyloid peptide (Aβ) and a prion-peptide in AD (97)
Acotiamide hydrochloride	A selective, reversible AChE inhibitor improved clonidine-induced hypomotility.		Used for treatment of functional dyspepsia (98).
Corydaline	Inhibits AChE in a dose-dependent manner.	Inhibits pro-inflammatory cytokines expression (TNF-*α*, IL-6) in LPS-challenged macrophages.	
Donepezil	Centrally acting reversible AChEI and also upregulates nAChR in neurons (99, 100).	It shows anti-inflammatory effects and prevents BBB degradation by modulating MMP-2/9, NGF/proNGF, IFN-*γ*/IL-4, and p-Akt in EAE (101). Pre-treatment with donepezil suppressed TNF-α−induced sustained intracellular Ca^2+^ elevation via the PI3K pathway in rodent microglial cells. It also suppresses NO production and increases the phagocytic activity of mouse primary microglial cells (102). In macrophages, donepezil reduces inflammatory cytokines (IL-1β, IL-2, IL-6, IL-18, and TNF-α) and attenuates LPS-induced nuclear translocation of NF-*κ*B (103). It inhibits RANK-induced bone degradation by inhibiting osteoclast differentiation (104).	It is mainly used to treat AD, PD, Schizophrenia, and depression (105–106).
Choline alfoscerate (α-GPC)	Parasympathomimetic acetylcholine precursor, acting as acetylcholine release promoter (107).	–	–
Cisapride	Stimulate serotonin receptors mediated increases of acetylcholine release in the enteric nervous system (108).	–	–
Curcumin	Stimulates vagus nerve and enhance ACh biosynthesis by upregulating enhanced ChAT activity and expression of VAChT in murine RA model. (109) Upregulates gene expression of M_1_ and M_3_ mAChR, choline acetyltransferase, and GLUT3 in the cerebral cortex of diabetic rats (110).	–	–

### Choline Transporter (ChT) and Vesicular Acetylcholine Transporter (VAChT)

ChTs are expressed on the cell membranes of cholinergic neurons in presynaptic terminals and regulate the ACh reservoirs during autonomic, cognitive, and motor functions (111). This membrane protein helps transport the precursor molecule choline into the neurons for the synthesis of ACh (111). ChTs are predominantly found on the plasma membrane of microvascular cells. They are also highly expressed on the mitochondrial membrane, where they are involved in choline oxidation upon absorption. Bone marrow-derived macrophages treated with lipopolysaccharide (LPS) show increased transcript and protein expression of the choline transporter-like protein-1 (CTL1) (112). The human leukemic T-cell line expresses *CHT1* mRNA and mediates choline uptake in T cells (113). Activation of protein kinase C causes inhibition of CTL1 in macrophages, thereby causing altered cytokine secretion (114). The distribution of ChT on different immune cells and its importance in the specific tissue microenvironment in controlling inflammation and immunity need to be further investigated.

ACh is packaged into the secretory vesicles by a specific transporter protein VAChT using an exchange of protons (H^+^) (115). Most cholinergic neurons in the brain, spinal cord, and NMJ with skeleton muscle express VAChT (116). Alteration of VAChT expression has consequences on the concentration of acetylcholine loaded in the secretory vehicle, thus indirectly maintaining the neurotransmitter release. Dysregulation of VAChT has been reported in several diseases like AD, Epilepsy, and Sepsis (117–119). VAChT knockdown (VAChT-KD) mice, upon LPS challenge, show increased susceptibility to inflammation and greater mortality. LPS challenge increases the levels of proinflammatory cytokines (TNF-α, IL-1β, and IL-6) in the spleen, brain (120). Human melanocytes, keratinocytes, and alpha-cells also express non-neuronal VAChT, thereby regulating the acetylcholine release and cholinergic activity (121, 122). The expression and function of VAChT on immune cells are still unclear and thus have potential physiological consequences in the peripheral immune response.

### Acetylcholine Receptors (AChRs)

The diversified role of ACh is governed by different types of receptors, known as cholinergic receptors (AChRs), which are classified according to their affinity for various chemical ligands (123).

#### Nicotinic Acetylcholine Receptors (nAChRs)

**Figure 2:**
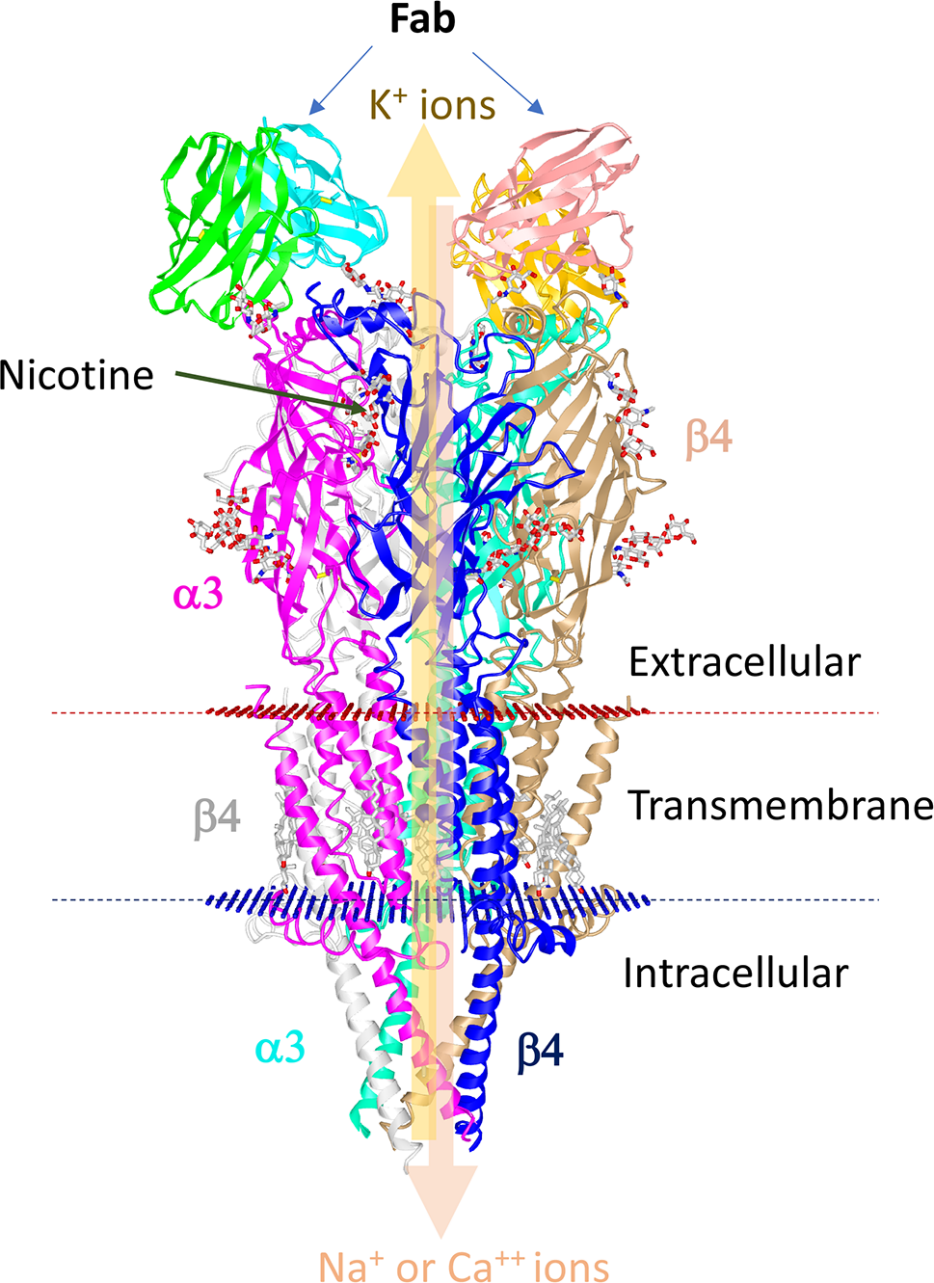
Structure of human α3β4-nAChR complexes with nicotine.**** The human nAChR complex with α3β4 nicotine acetylcholine receptor (Protein data bank Id: 6PV7) is displayed using the online iCn3D software. The structure is composed of two α3 chains and three β4 chains. The Fab fragments of the antibody used for stabilization of the sample are shown at the top. Nicotine is shown in balls and sticks. Red and blue discs represent the plasma membrane. The thick arrow depicts the regulation of the movement of ions by the central pore.

These receptors respond to the ligand, nicotine. These ligand-gated ion channels are composed of four distinct subunits (α1–10, β1–4, γ, and δ) bound in different stoichiometric ratios around a central pore with the help of ϵ subunits **(** **Figure 2** **)**. These receptors exist as homomers (with all subunits of one type), such as (α7)_5_, or as heteromers with at least one α and one β subunit among the five subunits that are combined in various combinations, such as (α4)_3_(β2)_2_, (α4)_2_(β2)_3_, (α3)_2_(β4)_3_, α4α6β3(β2)_2_ (124). The structure of the human α3β4-nAChR complex, solved using cryoelectron microscopy (125), is shown in **Figure 2**. This structure shows a central channel formed by different subunits of nAChRs. The channels help move ions from the extracellular environment to the cytoplasmic side or vice versa in the cells after stimulation with specific ligands **(** **Figure 2** **)**. The α3β4-nAChR subtype is located in the autonomic ganglia and adrenal glands, which forms the main relay between the central and peripheral nervous systems in the hypothalamic-pituitary-adrenal axis (HPA) axis upon activation (126). Diverse nAChR subtypes confer differential selectivity for nicotinic drugs in the central and peripheral nervous system, muscles, and many other tissues (127). The ligand binds to the specific site of the receptor leading to the triggering of all subunits of nAChR to change conformation, resulting in the opening of a non-selective cation channel, which then regulates the movement of ions (128).

In the immune system, nAChRs are known to regulate inflammatory processes (129). Pathological causes in acquired neurodegenerative diseases, such as autoimmune ganglionopathy, autoimmune encephalitis, and MG, are caused by an autoimmune reaction against nAChRs in the NMJ (130–132). Non-neuronal nAChRs are also involved in the pathogenesis of palmoplantar pustulosis, psoriasis, and rheumatic diseases (133–135). Overexpression of nAChR in gastric, colorectal, pancreatic, liver, lungs, and breast tumors appears to regulate cancer cell processes such as proliferation, apoptosis, angiogenesis epithelial-mesenchymal transformation (136, 137). Several of the nAChR agonists and antagonists are known to work in a receptor-specific and selective manner. Some of the agonists and antagonists are listed in **Tables 3** and **4**. Treatment with these ligands and their effect on immune cells is not very well studied and needs detailed investigation.

**Table 3:** Agonists of acetylcholine receptors (AChRs).

Agonists	Cholinergic effects	Effect of immune status	Experimental models
**Pan-cholinergic agonists**			
Acetylcholine chloride	Non-selective cholinergic agonist mimics the effect of the endogenous compound acetylcholine.		Its multi-faceted action, toxicity, and rapid inactivation by cholinesterase do not offer a therapeutic value.
Carbachol	Non-selective cholinomimetic agonist stimulates both muscarinic and nicotinic receptors.	Carbachol treatment reduces the expression of IL-1β; MHC-II, CD86, and IL-12p70 in splenic DCs at the early phases of sepsis (138). It reduces the release of inflammatory cytokines (TNF-α, IL-1β, and IL-6) and expression of caspase-3 in myocardial cells and improves the cardiac function and survival rate from sepsis in rats (139). Carbachol increases the expression of inflammatory genes (IL-6, IL-8, and cyclooxygenase-2) in smooth muscles (140).	Used for treatment of glaucoma in humans (141).
**Selective to nicotinic receptors**			
Nicotine	Nicotine induces non-selective activation of nAChRs.	Nicotine-treated cells produce lower Th1 cytokines (IL-2 and IFN-*γ*), but significantly higher Th2 cytokines (IL-4 and IL-10) (142). Nicotine suppresses IL-18-mediated systemic inflammatory responses and downregulates expression of TNF-α, IL-12, and IFN-*γ* in PBMCs (143).	Nicotine has anti-inflammatory and depressive activity in neurodegenerative and depressed patients (144, 145).
Cotinine	Activates, desensitizes nAChR at a much lower potency than nicotine.	A high cotinine concentration stimulates extracellular ROS generation and oxidative stress-mediated tissue damage by activated neutrophils (146). Pre-treatment of monocytes with cotinine mounts IL-10-dominated anti-inflammatory response via α7-nAChR through PI3K/GSK-3β-dependent pathway (147).	
ABT-418		ABT-418 has a neuroprotective effect on rat cortical cells to glutamate (Glu)-induced cytotoxicity mediated via interaction with α7-nAChRs (148).	A clinical trial of ABT-418 was conducted to treat adults with attention deficit hyperactivity disorder and AD (149, 150).
Epibatidine	Binds to the α4/β2-nAChR and also binds to the α3/β4-nAChR subtype.	Stimulation of α4β2 nAChRs with epibatidine increases the IgM-mediated proliferation of B cells (151). *In vivo* administration of epibatidine (5 μg/kg, s.c.) increases plasma corticosterone levels and reduces the lymphocyte proliferation in the presence of concanavalin A (152).	
Succinylcholine chloride	Irreversible and competitive agonist on muscle type (α1)2β1δϵ-nAChR (153). It is resistant to acetylcholinesterase and is quickly degraded by plasma butyrylcholinesterase.	Patients who received succinylcholine as an anesthetic had lower CD4/CD8 frequency and IgE levels in their peripheral blood. It also changes the oxidative state of lymphocytes by impairing glutathione levels and prompting T cells to produce more reactive oxygen species (ROS) (154, 155).	
PNU-282987	Selective α7-nAChR agonist	PNU-282987 has a protective function in the lung injury model. PNU−282987 inhibits TNF−α and IL−6 release and decreases the phosphorylation levels of p38, JNK, and ERK in peritoneal macrophages (156). In the bronchoalveolar microenvironment, PNU-282987 reduces the neutrophil recruitment and inflammatory cytokines secretion (157). It also has an anti-inflammatory role in NK cells by reducing the NF-κB levels and its translocation to the nucleus, down-regulating the expression of NKG2D receptors, and inhibiting IFN-γ secretion and NKG2D-dependent NK cell cytotoxicity (158)	It has been used as an anti-inflammatory therapy in animal models of diseases such as airway inflammation, cardiomyopathies, and AD (159–160).
Cris-104	Neuronal α4β2-nAChR agonist	Cris-104 increases nor-epinephrine concentration and increases neuronal activity in the brain, thus having an anti-nociceptive efficacy in rodent models of acute and chronic pain (161).	
PHA-543613	Selective α7-nAChR agonist	PHA-543613 suppresses CDC42 and MMP2 mRNA expression in macrophages (162). Administration of PHA-543613 induces activation of PI3K/AKT/GSK-3β to reduce neuroinflammation and oxidative stress (163, 164).	It’s being studied as a potential cure for cognitive deficits in schizophrenia, PD, and intracerebral hemorrhage (165, 166).
NS6740	Silent non-ionotropic agonist of α7-nAChRs but an effective modulator of the cholinergic anti-inflammatory.	NS6740 shows an anti-inflammatory property in LPS challenge microglial cells by reducing TNF-α release (167).	
GAT107	Positive modulator of α7-nAChR	GAT107 shows a dose‐dependently attenuation of CFA‐induced inflammatory pain by reducing phosphorylation of intracellular p38MAPK (168). In macrophages, GAT107 improves superoxide dismutase 1 activity, Nrf2, and hemeoxygenase-1 expression (169).	
AR-R17779	Selective α_7_-nAChR agonist	AR-R17779 has a protective role mediated by α_7_-nAChR in intestinal colitis and post-operative infections model (170, 171). In CFA-induced arthritis, it plays a contradictory role. It decreased TNF-α levels in plasma and synovial tissue, as well as exacerbates arthritis (172, 173).	
Nifene	Selective α4β2-nAChR receptor partial agonist		^8^F-Nifene is used in PET and SPECT imaging agents to screen lung cancer (174).
**Selective to muscarinic receptors**			
Muscarine	Non-selective agonist of the mAChR (175). It has both excitatory and inhibitory effect on ACh release at NMJ due to differential binding to various mAChRs.	Intravenous administration of muscarine chloride increases IgA secretion from the perfused intestinal loops in rats (176).	
L-Satropane	mAChR agonists	L-satropane defends against CoCl2-induced neurotoxicity by increasing retinal neuron survival in a dose-dependent manner. L-satropane substantially reverses the Aβ production (177).	
Oxotremorine (Oxo-M)	Non-selective (mAChR) agonists with positive allosteric modulation via M_4_ subtype.	Oxo-M promotes TCR/CD3ε-induced IL-2 secretion in human PBMCs. It also increases the cell surface expression of CD2, CD3, CD4, CD8, and IL-25 (178, 179) and promotes T cell proliferation (180).	
McN-A-343	Selective M1 mAChR agonist, however, is partial agonist with a similar affinity at all five mAChR.	McN-A 343 therapy results in a substantial reduction in colitic score. McN-A-343 therapy reduced colonic inflammation and decreased pro-inflammatory Th1/Th17 colonic and splenic DC cytokine secretion mediated by the 7nAChR and NF-kB signaling pathways. CD4^+^ T cell priming was diminished after cholinergic activation (181) McN-A-343 inhibits endotoxin-induced systemic TNF-α levels in a dose-dependent manner (182)	
Cevimeline	Stimulates SSN neurons mainly by M_1_ mAChR and M_3_ mAChR.		Orally administered in the treatment of Sjogren’s syndrome (183).
Bethanechol chloride	Muscarinic agonist selectively activates M_2_ mAChR.	Suppresses tumorigenesis through MAPK and PI3K/AKT signaling (184). Bethanechol treatment of bone marrow-derived macrophages upregulates M_3_ mAChR gene expression and induces a classically-activated macrophage phenotype (185).	It has a bactericidal effect and increases intracellular cyclic GMP levels in the patient suffering from hidradenitis suppurativa (186). Administered to treat urinary retention and gastrointestinal motility (187, 188).
Arecaidine propargyl Ester (APE) and Arecaidine But-2-ynyl Ester Tosylate (ABET)	Highly selective M_1_ mAChR and M_2_ mAChR agonist.	APE treatment inhibits the proliferation of cancer stem cells in glioblastoma multiforme by lowering the expression of mir210 in hypoxia conditions (189).	
7,8-dihydroxyflavone (7,8-DHF)	Positive allosteric modulator increased M_3 _mAChR.	It inhibits iNOS and COX-2 expression and reduces the synthesis of NO and PGE2. Besides, 7,8-DHF blocks the release and expression of inflammatory cytokines such as TNF-α and IL-1 (190, 191).	It shows a therapeutic efficacy for treating Alzheimer’s disease, Huntington’s disease, and schizophrenia in the animal model (192–193).
Amiodarone	Gq-mediated responses are positively modulated at M_1_ mAChR and M_3_ mAChR but inhibited in a more discriminating fashion at the M_1_ mAChR (194, 195).	TNF-α, IL-6, of IL-1β production, was inhibited by amiodarone at 0.1-1 µM concentration. Modulation of IL-6 and IL-1β production by amiodarone was biphasic and significantly increased at a concentration beyond 10 µM (196).	Amiodarone is an anti-arrhythmic drug used to treat several congestive heart failure (197, 198).
Xanomelin	M_1_/M_4_ mAChR preferring muscarinic agonist.	Xanomelin suppresses TNF-α and IL-6 levels and improves survival in an endotoxemia model. Treatment with *ex vivo* endotoxin-stimulated splenocytes shows significantly less sensitivity to inflammatory activation and lower secretion of TNF-α, IFN-γ, MCP1, IL-6, and IL-10 (199).	It was used in the treatment of both Alzheimer’s disease and schizophrenia (200–201).
Dihydroquinazolinone	Selective and CNS-penetrant M_1_ mAChR and M_4_ mAChR agonists.	It shows a potent inhibitor of p38alpha MAP kinase and suppresses TNF-α production in LPS-stimulated PBMCs (202).	–
Clozapine	Agonist at the M_4 _mAChR and antagonized agonist-induced responses at the other four mAChR.	Clozapine inhibits T-bet expression and promotes STAT6 and GATA3 expression in PBMCs (203). Clozapine therapy inhibits the production of IL-6, IL-8, and IL-12 and increases the production of IL-10 in LPS-stimulated macrophages (204). In neutrophils, clozapine increases cell surface Mac-1 expression and activates the AKT signaling pathway and phagocytosis of bacteria (205).	It is a highly effective antipsychotic medication (206).

**Table 4:** Antagonists of selective acetylcholine receptors (AChRs).

Antagonists	Cholinergic effects	Effect on immune status	Experimental models
**Selective to nicotinic receptors**			
Hexamethonium	Nicotinic receptor blocker	Hexamethonium blockade of peripheral nAChR increases neutrophil migration and mechanical hyperalgesia in the RA model (207). It suppresses stress-induced mast cell activation and inhibits elevated PGE2 levels after exposure to stress (208).	–
α-Bungarotoxin (α –BTX)	Bind to nAChRs in an irreversible antagonistic manner, blocking ACh’s activity at the post-synaptic membrane, inhibiting channel opening and ion flow, and cause paralysis (209).	α –BTX treatment to T cells activated by sub-optimal PHA concentrations causes blockade of α7-nAChR that enhance T cell proliferation (210).	–
Mecamylamine	To neutralize the effects of nicotine, it is used as a competitive non-selective (α3β4, α4β2, α3β2, and α7) nAChR antagonist	Mecamylamine reverses the ant-inflammatory role of nicotine in the nAChR-mediated cholinergic pathway.	Mecamylamine is licensed for the treatment of hypertension. It attenuates all of the nicotine and cigarettes symptoms, including seizures, rendering it an important pharmacotherapy for tobacco addiction (211).
Dihydro-beta-erythroidine	Selective α4β2-nAChR antagonist (161).	–	–
Dextromethorphan (DXM)	α3β4-nAChR, α4β2-nAChR, and α7-nAChR antagonist in the cholinergic pathway (212). Also, It is a selective antagonism of N-methyl-d-aspartate receptors and/or show interaction with opiate receptors (213).	DXM decreases the expression of CD40, CD80, CD86, MHC class I, and MHC class II in both murine BMDCs and human monocyte-driven DCs upon LPS challenge. DXM pre-treatment results in dose-dependent substantial reductions in TNF-α, IL-6, IL-12, and ROS production. It inhibits the ability of LPS-stimulated BMDCs to promote ovalbumin-specific T cell proliferation by downregulating MAPK and NF-*κ*B pathways (214). DXM is neuroprotective in cerebral ischemia models, spinal cord injury, PD, and epilepsy by downregulating NADPH oxidase, thus, reducing superoxide free radicals and intracellular (ROS) (215, 216). It prevents immune cell filtration, inhibits NOX2 activity, and has an anti-inflammatory effect in EAE (217). Proinflammatory cytokines (TNF-α, IL-6, and IL-17A) expression levels decrease in CIA mice and RA patients. In collagen-reactive CD4^+^ T cells, DXM reduced the production of anti-CII IgG, IFN-*γ*, and IL-17A (218).	DXM is under development for the treatment of depression, AD, ALS, and neuropathic pain (219–220).
Methyllycaconatine (MLA)	α7-nAChR antagonist	MLA (2.4 mg/kg per day) treatment in acute viral myocarditis increases the frequency of Th1 and Th17 cells, lowers the frequency of Th2 and Treg cells in the spleen. It also increases proinflammatory cytokines, cellular infiltration, and severity of myocardium lesions in viral myocarditis (221).	–
N,N-decane-1,10-diyl-bis-3-picolinium diiodide (bPiDI)	Selective α6β2-nAChR antagonist (222). Nicotine-evoked dopamine activation and nicotine reinforcement are mediated by α6β2-nAChRs expressed by dopaminergic neurons. bPiDI blocks nicotine’s effects on these receptors, making them therapeutic targets for nicotine addiction (223).		
α-conotoxins	Specific to α3β2-nAChR, α9α10-nAChR, and α3β4-nAChR (224).	α-conotoxins (5.5 μM) increases IL-10 production in Tregs and decreased IL-17 production in T cells (225). In PMA-activated macrophages, α-conotoxins upregulate the TNF-α and IL-6 in a concentration-dependent manner (226).	–
SR16584	High affinity for α3β4-nAChR and 10 nM for α4β2-nAChR (227, 228).	–	–
18-methoxycoronaridine (18-MC)	Highly selective α3β4-nAChR antagonist (229).	–	–
AT-1001	High-affinity and selective to α3β4-nAChR (230).	–	In humans with Th1-mediated celiac disease, it plays a therapeutic role by inhibiting cell permeability (231).
MG 624	α7-nAChR antagonist (232).	–	–
**Selective to muscarinic receptors**			
Atropine	A nonspecific antagonist that competitively inhibits acetylcholine (ACh) at postganglionic muscarinic sites and CNS (232). Abolish the effect of vagus nerve stimulation.	Prior to the LPS-induced activation of the inflammatory response, atropine decreases TNF-α and raises IL-10 plasma levels without affecting IL-6 production. This reduction in TNF-α improved the rate of survival from endotoxic shock in mice (233). Suppresses T cell proliferation and proinflammatory cytokine production in turpentine-induced inflammation. In reaction to the potent neutrophil/macrophage chemoattractant fMLP, atropine therapy decreases both chemokinesis and chemotaxis of PBMCs (234, 235).	–
Hyoscyamine	Non-competitively inhibits acetylcholine (ACh).	In the acute lung injury model in rats, hyoscyamine derivatives cause substantial reductions in TNF-α, IL-6, IL-1, and p38MAPK, NFB, and AP1 activation, as well as TLR4 expression (236).	–
Scopolamine hydrobromide	A non-selective muscarinic acetylcholine receptor (mAChR).	Scopolamine hydrobromide treatment shows upregulation of TLR3, TLR7, TLR8, and cytokines such as IL-4 and IL-10 (237). Mice treated with scopolamine show an increased density of CD4^+^, CD11c^+,^ and CD11b^+^ cells. And also show elevated levels of IL-1β, IL-2, IL-6, IL-12Rβ1, IL-17A, IL-17R, IFN-γ, and TNF-α transcripts (238).	Used for treatment of motion sickness, and GI obstruction (239, 240)
Gallamine Triethiodide	Non-competitive inhibition by altering the affinity of the agonist for its binding site.	–	–
VU0255035	Selective M_1_ mAChR antagonist	–	–
Pirenzepine	Selective M_1_ mAChR selective antagonist.	–	Used in peptic ulcers and also reduces muscle spasms (241, 242).
Methoctramine	Selective M_2_ mAChR antagonist	Methoctramine increases the high-frequency component of heart rate variability and inhibits systemic TNF−α release by activating muscarinic receptors (182). Methoctramine abolishes the ACh-elicited anti-apoptotic property and reduces the TNF-α-activated apoptotic pathway via EGFR-PI3K signaling in cardiomyocytes (243).	–
AF-DX 384	Selective M_2_ mAChR and M_4_ mAChR antagonist.	–	–
Darifenacin	Selective M_3_ mAChR antagonist	–	Effectively used for the treatment of overactive bladder disorder (244).
4-diphenylacetoxy-N-(2-chloroethyl)-piperidine (4-DAMP mustard)	Selective M_1_/M_3_ mAChR antagonist.	4-DAMP abolishes mAChR-mediated immunoglobulin class switching to IgG in B cells. It inhibits the production of IL-6 and the maturation of B cells into IgG-producing plasma cells (245). M_3_ mAChR-mediated IL-8 expression in regulating inflammatory response via PKC/NF-κB signaling axis is completely antagonized by 4-DAMP (246). It also inhibits human T cell growth by inhibiting M1 mAChR-mediated expression of both IL-2 and IL-2R (179).	–
Tropicamide	M_4_ mAChR antagonist responsible for increased phosphorylation of AMPA receptor.	–	Inhibiting cholinergic stimulation responses, producing dilation of the pupil and relaxation of the ciliary muscle in ophthalmic surgery (247).

#### Muscarinic Acetylcholine Receptors (mAChRs)

**Figure 3:**
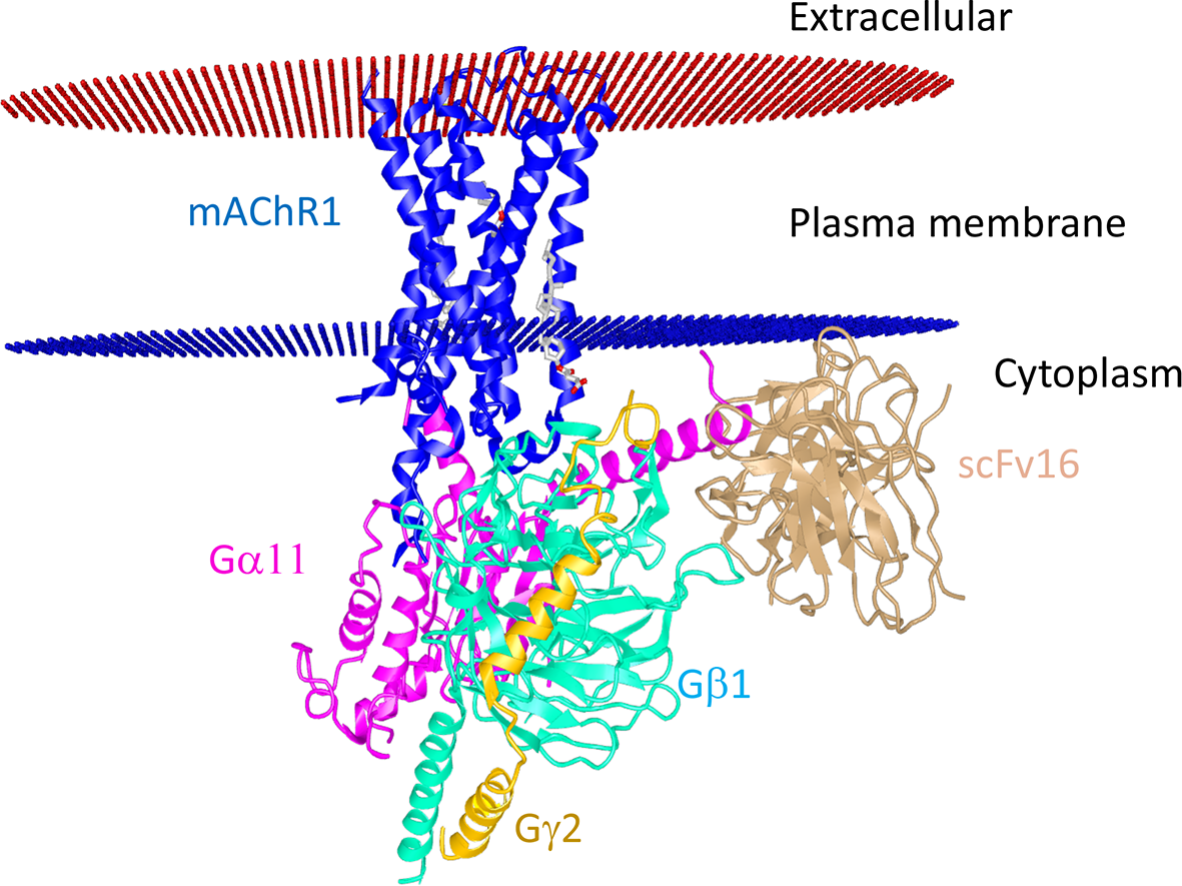
Structural interaction of M_1_ mAChR with the G protein-coupled receptor. The human muscarinic acetylcholine receptor 1G11 protein complex (Protein data bank Id: 6OIJ) structure (3.3 Å resolution) is displayed using the online iCn3D software. M_1_ mAChR interacts with G-proteins α11 and *γ*2-β1. The scFV16 nanobody used for stabilizing the structure is also shown. The allosteric ligand is shown in the ball and stick. Red and blue discs represent the plasma membrane.

These are metabotropic receptors consisting of seven transmembrane subunit G protein-coupled receptors (GPCRs) that respond to ACh and muscarine (248). M_1_ to M_5_ mAChR subtypes share 64 to 68 percent sequence identity and 82 to 92 percent sequence similarity, indicating that they have a high degree of sequence homology. Their G-protein coupling preferences and physiological functions, however, are different. mAChRs have been separated into two groups based on their functional coupling. The M_1_ mAChR, M_3_ mAChR, and M5 mAChR are coupled to G_q/11_ proteins, which mediate the activation of phospholipase C (PLC) activity (249). The M_2_ mAChR and M_4 _mAChR are coupled to the G_i/o_ protein, which mediates inhibition of adenylate cyclase (AC) and thus causes a decrease in cyclic adenosine monophosphate (cAMP) (248). Based on the physiology and distribution of the individual receptor, mAChRs can trigger different signal transduction pathways in the cells in a tissue-specific manner. Recently, the structures of the human M_1_ mAChR and M_2_ mAChR2 with G-protein complexes were published (250). The structure visualization of human M_1_ mAChR with the G-protein complex is shown in **Figure 3**. mAChRs have an extracellular ligand-binding domain and a transmembrane and intracellular signaling domain. The intracellular domain interacts with G proteins and other signaling molecules and helps intracellular signaling **(** **Figure 3** **)**. mAChRs are abundant in the hippocampus, cortex, thalamus, gastric and salivary glands, smooth muscle, and cardiac tissue, each having a specific downstream signaling cascade. Thus, the structural differences, ligand specificity, and functioning mechanism help understand each receptor’s roles within specific tissues. In the murine endotoxemia model, muscarinic receptor-mediated cholinergic signaling in the forebrain regulates peripheral immune function and inflammation to suppress serum TNF-α levels (251). Conversely, the major cause for the pathogenesis of autoimmune Sjögren’s syndrome is the production of auto-antibodies against the M_3_ mAChR (252). Autoantibodies against muscarinic receptors also triggered chronic immune activation in patients with chronic fatigue syndrome and periodontitis (253, 254). Patients with airway inflammatory infections/allergic rhinitis had increased expression of M_3_ mAChR mRNA and protein (255). However, the specific patterns of mAChR subunit distribution in tissues and expression in particular immune cell types are not well defined. Some of the selective mAChR agonists and antagonists are listed in **Tables 3** and **4**, respectively. Given the diverse distribution of mAChRs in different immune cells, the mechanism by which selective ligands alter specific immune cells in the tissue microenvironment during inflammation and immunity needs detailed investigation.

### The Role Played by the Cholinergic System in Various Immune Cells

The neuronal and lymphoid cholinergic system evokes various downstream functional and biochemical effects through AChRs present on immune cells. The importance of different components of the complex cholinergic system in different immune cells is discussed below-

#### Role in T Cells

T cells and their effector and regulatory function play an important role in inflammation and autoimmunity (256, 257). Some splenic and intestinal T cell subsets have been found to express functional ChAT and produce ACh (ChAT^+^ T cells) (21, 258). Most of these T cell subsets are found in the vicinity of catecholamine splenic nerve fibers forming a cholinergic non-neuronal reservoir (259). The ACh produced in the microenvironment activates α7-nAChR on T cells and facilitates the activation and proliferation of T cells (260). *In vitro* administrations of nicotine or ACh in a micromolar range inhibits DC mediated-T cell proliferation and differentiation, as well as reduce CD28 and cytotoxic T-lymphocyte-associated protein 4 (CTLA-4) expression and diminished maturation in T cells (261, 262). Endogenously released ACh in human leukemic T cell upregulates several Ca^2+^-permeable ACh-gated ion channels. Nicotine also impairs antigen receptor-mediated signal transduction in lymphocytes and causes T cell anergy by arresting cells in the G0/G1 phase (263). It is also known to alter the expression of co-stimulatory and adhesion molecules (such as ICAM and CD44) on immune cells and suppress the production of inflammatory cytokines (TNF-α, IFN-*γ*, and IL-6) (264). Nicotine binds to various forms of nAChR on T cells with variable affinity and mediates the apparently paradoxical effects of fostering cell survival while also causing apoptosis by inducing expression of FasL and survivin gene (265). The receptors, α7-nAChRs and α4-nAChR are predominantly known to be involved in CD4^+^ T cell proliferation and function (266, 267). The α7-nAChR antagonist, α-bungarotoxin (α-BTX), and methyllycaconitine (MLA) show an increased proliferative response to the T cell mitogen, phytohemagglutinin (PHA) (268). Activation of the α7-nAChR by nicotine in experimental autoimmune encephalomyelitis (EAE) mice model ameliorate clinal symptoms by directing naive CD4^+^ T cells towards the IL-4-producing Th2 phenotype and subsequently leads to decreased production of Th1 cytokines (such as TNF-α, IFN-γ, IL-2) and Th17 cytokines (such as IL-17, IL-17F, IL-21, and IL-22). However, activation of α7-nAChR with agonist does not affect the differentiation of Th17 cells (269). α7-nAChR activation with nicotine in human PBMC and CD4^+^ T cells results in a similar reduction of IL-17 production, suggesting that it has an anti-inflammatory property (270). Nicotine-induced activation of α7-nAChRs also increases the suppressive function of CD4^+^CD25^+^Tregs/CD4^+^CD25^−^ T-cell by up-regulation of CTLA-4 as well as Foxp3 expression and decreased IL-2 secretion (271). These studies suggest that nAChRs activation can modulate the function of various subsets of CD4^+^ T cells.

In humans, all five subtypes of mAChRs are known to be expressed on lymphocytes. However, each receptor’s expression pattern differs among different cell types in an individual (29). PHA is known to increase M_5_ mAChR mRNA expression *in vitro*, while lymphocytes stimulated with phorbol 12-myristate 13-acetate (PMA), a protein kinase C activator, plus ionomycin, a calcium ionophore, increase M_3_ and M_5_ mAChR mRNA expression, demonstrating that differential expression of mAChR is caused by different immunological stimulations (272). Activation of the M_3_ mAChR using methacholine on CD4^+^ T cells isolated from airway inflammatory infections/allergic rhinitis patients leads to increased production of IL-4 and TNF-α (255). Treatment of Jurkat cell lines with the mAChR agonist, oxotremorine (OXO-M), increases the expression of IL-2 receptors on lymphocytes and enhances the PMA-induced IL-2 secretion (273). Interestingly, it was found that arecoline, a non-selective muscarinic agonist improves cognitive function and memory in AD (274). Chronic treatment with arecoline leads to a reduction in the size of the spleen, thymus, and mesenteric lymph nodes, as compared to untreated control mice. *In-vitro* arecoline treatment was shown to reduce lymphocyte proliferation and IL-2 production (275). M_3_ mAChR knockout (M3^-/-^) mice were reported to have trouble in clearing bacterial and helminth infections. The absence of M_3_ mAChR also led to delayed expulsion of *Nippostrongylus brasiliensis* due to inhibition of smooth muscle contraction, a reduction in the activation of CD4^+^ T cells, and lower levels of expression of IL-4 and IL-13. The activation of the M_3_ mAChR, specifically with ACh on activated T cells, increases IL-13 and IFN-γ cytokine production (276). The distribution of the M_1_-M_5_ mAChR on each subset of CD4^+^ T cells in humans and mice has not been systemically studied and is therefore open for further investigation.

#### Role in B Cells

Mouse B cells are known to express α4, α5, α7, β2, and β4 subunits of nAChR, with the expression of different subunits being regulated at the various stages of B cell maturation (277). At the primary level, nAChRs are required for the development and survival of B lymphocytes within the primary lymphoid tissues and spleen. α4 and β2 knockout mice have reduced B cell populations, underscoring their critical role in regulating lymphocyte survival (278). The CD19^+^B220^+^IgM^+^ B lymphocytes mainly expressed α7, α4, and β2 subunits of nAChRs. Mice deficient in these subunits of nAChRs showed reduced amounts of serum IgG, while β2^−/−^ mice had a reduced number of IgG-producing cells in the spleen. However, the IgG response to horse cytochrome *c* in α4 and β2 knockouts was stronger than in wild-type (WT) mice, with the β2^−/−^ mice having high cytochrome *c*-specific antibodies after immunization (279). Also, α7-nAChR influences IgM antibody production but not IgM to IgG class switching (280). α7-nAChR is constitutively expressed on CD5^+^CD1d^+^ regulatory B lymphocytes, which increases with the activation of B lymphocytes. Inhibiting α7-nAChR with methyllicaconitine inhibits CD40-mediated B lymphocyte proliferation (281). α4β2-nAChR, α7-nAChR, and α9α10-nAChR on B lymphocytes are differentially involved in B cell-mediated immune cell interactions. α7 and α9(α10) subunits of nAChRs are linked to CD40-mediated B cell proliferation, while the α4β2-nAChR is linked to IgM antibody production (151). However, the initial levels of IgM in WT and ${\text{M}}_{1}/{\text{M}}_{5}^{-/-}\text{double}$ knockout mice were similar. The OVA-antigen-specific IgG1 levels in ${\text{M}}_{1}/{\text{M}}_{5}^{-/-}\text{KO}$ mice were significantly lower, suggesting that mAChRs are not required for antibody production but are involved in immunoglobulin class switching (282). M_1_ mAChR knockout (${\text{M}}_{1}^{-/-}$) mice show increased splenic noradrenaline production and a decrease in the number of IgG-producing B cells (283). M_3_ mAChR in B cells show an increase in calcium signaling and c-Fos gene expression, thereby affecting several downstream signaling pathways (284). However, an autoantibody against the M_3_ mAChR has been reported in the immunopathogenesis of Sjögren’s syndrome and correlates with a significant risk of developing B cell lymphoma (285). Together, these studies suggest that AChRs contribute to mounting an effective humoral response. A detailed investigation of cholinergic systems’ expression in the different developmental and differentiation stages of B cells needs to be investigated, and its clinical importance in the inflammatory disease yet to be established.

#### Role in Dendritic Cells (DCs)

Nicotine impairs the capability of DCs to capture antigens and reduces the responsiveness of DCs to maturation stimuli. The nicotine-treated DCs fail to produce IL-12, IL-1β, IL-10, and TNF-α and thus unable to induce APC-dependent T cell responses and Th1-cell polarization. DCs exposed to nicotine tend to polarize CD4^+^ T cells towards the Th2 phenotype. These show increased expression of OX40L and significantly high amounts of IL‐4, IL‐5, and IL‐13 (261, 286). CD205^+^ DCs express mRNA encoding secreted lymphocyte antigen-6/Urokinase-type plasminogen activator receptor-related peptide (SLURP)-1, an endogenous α7-nAChR allosteric ligand that stimulates DCs to produce ACh in an autocrine manner (260). Treatment with GTS-21, an α7-AChR agonist, showed a robust anti-inflammatory action during collagen-induced arthritis (CIA) in DBA/1 mice by modulating DCs. GTS-21 treatment down-regulated CD80 and MHC II expression on the surface of DCs, leading to suppression of its infiltrating capacity and differentiation. These inhibitory effects were successfully reversed by the α7-nAChR antagonist, methyllycaconitine (287, 288). Treatment of DCs with ACh also resulted in the upregulation of the macrophage‐derived chemokine (CCL22) in the thymus and the activation‐regulated chemokine (CCL17). CCL22 and CCL17 help in the recruitment of Th2 cells at the site of inflammation (289). In both mice and humans, DCs treated with nicotine utilize upregulation of notch ligands and nuclear receptor peroxisome proliferator-activated receptors γ (PPAR γ) to modulate the Th1/Th2 balance in favor of Th2 lineage (290). Activation of nAChRs on immature DCs (imDCs) has also been shown to have anti-tumorigenic effects. Activation of nAChR promotes the expression of co-stimulatory molecules CD80/CD86 and 4-1BBL on imDCs, thereby increasing their ability to stimulate T-cell proliferation. Transfer of nicotine-treated imDCs has been shown to reduce tumor growth by generating an effective cytotoxic T cell response in the tumor microenvironment (291).

DCs have been found to express the M_3_ mAChR, M_4_ mAChR, M_5_ mAChR, ChAT, and AChE (289). Methacholine, a synthetic choline ester, and a non-selective mAChR agonist were shown to increase expression of the OX40L on DCs, which helps in the interaction co-stimulation with T cells (255). Cholinergic activation using the M_1_ mAChR-specific agonist, McN-A-343, in colitis was found to decrease IFN-γ, IL-17, IL-12p70, and IL-23 in the splenic CD11c^+^ DCs (181). These studies suggest that AChRs affect the immune response by altering innate immune cells like DCs. Further detailed molecular mechanism of cholinergic receptor signaling in the differentiation and function of DCs under different inflammatory conditions and tissues needs to be investigated.

#### Role in Macrophages

The intracellular signaling of alveolar macrophages is mediated by α9, α10, and β2-nAChR. Macrophage populations previously exposed to nicotine show a lower rise in ATP-induced intracellular Ca^2+^ release, which is independent of STAT3 phosphorylation (292). The α7-nAChR on macrophages is vastly sensitive to ACh released by the ChAT^+^ T cells, and its interaction with ACh leads to reduced production and release of proinflammatory cytokines. Antigen-stimulated spleen cells from α7 knockout mice produce significantly higher amounts of TNF-α, IL-6, and IFN-γ than splenocytes from WT mice (264). Vagus nerve stimulation (VNS) for a duration of 0.1–60s in WT mice modulates cytokine release from macrophages *via* the α7-nAChR receptor. However, the α7 subunit deficiency rendered the VN ineffective in inhibiting TNF-α release (293). Priming macrophages with a cognate ligand for α7-nAChRs results in a pharmacological inhibition of AC which in turn increases the cAMP levels in the cells. Thus, activation of α7-nAChR in macrophages promotes expression and phosphorylation of c-FOS and CREB, required for a sustained decrease in the endotoxin-induced release of TNF-α (294). In LPS-stimulated human macrophages, ACh-induced activation diminishes pro-inflammatory cytokines like TNF-α, IL-1β, IL-6, and IL-18, but not the anti-inflammatory cytokine, IL-10 (295). VNS causes activation of nAChRs on macrophages, thus hampering their activation *via* the JAK2-STAT3 signaling pathway. nAChR antagonist treatment of macrophages causes enhanced expression of JAK2 and STAT3, which negatively regulate metalloproteinase 9 (MMP-9) production and inhibit macrophage migration (296, 297). It was found that nicotine treatment led to overexpression of IL-1 receptor-associated kinase M (IRAK-M), a negative regulator of TLR4 signaling, *via* α7-nAChRs. Upregulation of IRAK-M expression is required for the anti-inflammatory effect of nicotine on LPS-induced TNF-α production by peritoneal macrophages (298). In these macrophages, nicotine treatment significantly lowered ATP-induced intracellular Ca^2+^ signaling *via* β2-nAChR only (299, 300).

The muscarinic agonist, carbachol, causes a moderate enhancement in phagocytosis of zymosan particles by primary peritoneal macrophages (301). Depending on the local microenvironment, several mAChR subtypes were reported on differentiated resident macrophages. Activation of M_1_–M_3_ mAChR causes tumor macrophages to proliferate mainly by activating the arginase pathway, producing high prostaglandin E2, and promoting potent angiogenesis. Likewise, in normal macrophages, activation of M1–M_2_ mAChR- triggers protein kinase C activity and induces moderate prostaglandin E_2_ liberation for proliferation (302).

#### Role in Mast Cells

Treatment of patients suffering from allergic diseases with nicotine leads to suppressing the production of Th2 cytokines and cysteinyl leukotriene LTC_4_. Crosslinking of the high-affinity receptor of IgE on mast cells causes its activation. Upon activation, mast cells at the early phase release preformed inflammatory mediators, and in the late phase, they synthesize and secrete cytokines/chemokines and leukotrienes. Treatment with low concentrations of nicotine leads to suppressing the late-phase, but not of the degranulation response. α7/α9-nAChR antagonists, methyllycaconitine, and alpha-bungarotoxin, successfully reverses nicotine’s suppressive effect on the late-phase response (303). mAChRs have also been characterized in human mast cells as tissue-based mediators that regulate histamine release and control hypersensitivity (304, 305). Upon treatment with nicotine, the human basophil cell line, KU-812, and the human mast cell line, HMC-1, are known to express nAChRs, thereby corroborating ACh expression reports by several cell types outside the neuromuscular system (306).

Atropine, a non-selective mAChR antagonist, has been shown to reduce the permeability of colon tissue in patients with ulcerative colitis and also further diminish histamine release and disrupt the interactions between mast cells corticotropin-releasing factor (CRF), and eosinophils in the mucosal barrier (307). OXO-M, a stable agonist of mAChR, and physostigmine, an AChE inhibitor, suppress histamine release (308). Methoctramine, an M_2_ mAChR antagonist, has been reported to activate phosphoinositide breakdown at high concentrations *via* pertussis toxin-sensitive G proteins, with subsequent histamine (309). M_1_ mAChR signaling modulates phosphoinositide (PI) 3-kinases, which are critical regulators of mast cell degranulation (310). Mast cell degranulation requires IgE signaling and receptor-mediated calcium mobilization (311). The lethal toxin of *Clostridium sordellii* is known to inhibit Rac, thereby disrupting calcium turnover and blocking M_1_-mediated exocytosis in RBL 2H3-hm1 mast cells (312). Together, these studies suggest that AChRs can alter mast cell function and contribute to the pathogenesis of mast cell-mediated diseases.

#### Role in Neutrophils

The mRNA and protein expression of several nAChR subunits, such as α1, α3, α4, α7, β2, and β4, are also reported in human polymorphonuclear neutrophils (PMN). The α7, α4β2, and α3β4 subunits of nAChR on PMNs have been shown to have regulatory roles in their maturation at the site of inflammation (313). During inflammation, cell surface adhesion molecules play an integral role in the migration of neutrophils from lymphoid organs to the peripheral inflammatory site. nAChRs are known to regulate the expression of the cell surface protein, CD11b, on the surface of neutrophils. Nicotine administration and VNS significantly reduce surface expression of CD11b on neutrophils *via* suppression of F-actin polymerization, thereby reducing neutrophil attachment to the endothelium surface and transmigration to inflamed sites caused by microbial infection (314).

In mice, treatment with the non-selective cholinergic antagonist, atropine, led to an increase in the neutrophil population and increased serum corticosterone (CORT) concentrations in treated mice compared to WT mice (315). Neutrophil chemotactic activity is regulated by acetylcholine-mediated IL-8 release from epithelial cells *via* mAChR. ACh significantly stimulates the ERK1/2 and NFkB pathways, leading to an increase in chemotaxis by neutrophils, which can be reversed by tiotropium, an antagonist of the M_3_ mAChR (316). The M_3_ mAChR takes active participation in triggering cell death and contributes to the pathophysiology observed in several autoimmune diseases like vasculitic inflammation and thrombosis. The stimulation of M_3_ receptors on neutrophils induces neutrophil extracellular trap formation *via* the Akt, RAF/MEK/ERK pathway, ROS induction, and peptidyl arginine deiminase activation (317). Blocking the M3 mAChR reduces the proinflammatory effect of ACh on smooth muscles, as well as epithelial and endothelial cells. M_3 _mAChR knockout mice show altered neutrophil recruitment due to the downregulation of cell adhesion molecules like fibrinogen-α and CD177 (318). Sputum samples in healthy smokers and chronic obstructive pulmonary disease (COPD) patients have been shown to have increased TGF-β1 and ACh concentrations, consequently increasing neutrophil adhesion to epithelial cells. TGF-β1 depletion significantly reduces M_3_ mAChR and ChAT expression on epithelial cells, thereby establishing autocrine/paracrine feedback during neutrophilic inflammation (319). It is also noticed that during airway hyper-responsiveness due to infections, TNF-α production by neutrophils negatively regulates the M_2_ mAChR, causing vagally-mediated bronchoconstriction (320).

#### Role in Natural Killer (NK) Cells

NK cells play a very important role in several inflammatory and chronic diseases (321). Human NK cells show the complete cholinergic machinery expression, including ChAT, VAChT, AChE, and ChT1 (322). Upon acute inflammation, ChAT^+^ NK cells upregulate the synthesis of ACh to stimulate monocytes, modulate cytokine expression in the tissue microenvironment, and reduce inflammatory damages (322). Highly purified NK cells have been reported to express α4, α5, α6, β2, and β3 subunits of nAChR receptor (323). Under inflammatory disease conditions, NK cells increase the production of ACh by upregulation of ChAT enzymes. ChAT expression also increases along with the maturation of NK cells. Adoptive transfer of ChAT-expressing NK cells (ChAT^+^ NK cells) into the cerebral ventricles of CX3CR1^−/−^ mice reduces inflammation and autoimmune responses in the experimental autoimmune encephalomyelitis (EAE) model (322). ChAT^+^ NK cells have been shown to successfully reduce the infiltration of CCR2^+^Ly6C^hi^ monocytes and lower the secretion of proinflammatory cytokines. The anti-inflammatory effect of NK cells is mediated *via* α7-nAChRs. NK stimulation with cytokines (IL-12, IL-18, and IL-15) increases the transcription and translation of α7-nAChR (322). Activation of α7-nAChR in NK cells decreases their NK group 2D member (NKG2D)-dependent cell-mediated cytotoxicity and IFN-γ production, thereby showing anti-inflammatory properties during inflammation (158). β2-nAChR modulates NK cell functions *via* NF-κB-induced transcriptional activity in NK cells (323). Aberrant functioning of NK cells is the major cause of tumorigenesis and multiple cancers. It has been reported that single nucleotide polymorphisms (SNPs) of mAChR in natural killer cells result in dysregulation of Ca^2+^ signaling and reduced NK cell cytotoxic activity, leading to the pathophysiology observed in myalgic encephalomyelitis/chronic fatigue syndrome (324). The cytotoxicity of NK cells towards YAC-1 target cells was inhibited by the addition of ACh, suggesting that AChRs on NK cells control the cytotoxic function of NK cells. Furthermore, pilocarpine, an agonist of the mAChR, showed a similar effect on the cytotoxicity of NK cells when atropine was used to block the inhibitory effect of ACh (325). Together, these studies suggest that AChRs can affect the NK cell function in different inflammatory diseases.

#### Role in Eosinophils

Human peripheral blood eosinophils express the M_3_-M_5_ mAChRs, and activation of these mAChRs has an inhibitory effect on the activation of these cells (307, 326). Eosinophils play an important role in allergic disorders such as rhinitis, atopic dermatitis, and asthma. In ulcerative colitis, the cholinergic system in eosinophils at the mucosal barrier may contribute to mucosal inflammation (307). However, the role of mAChRs in eosinophils need detailed investigation.

#### Pre-Clinical and Clinical Importance of the Cholinergic System

The vagal efferent nerves originate at the medulla and innervate the GI tract, connecting it to the ENS. This gut-brain axis is known to regulate GI motility and secretion *via* vagal efferent fibers, which form cholinergic synapses in the ENS and respond to inflammatory stimuli (327). Cholinergic transmission between VN and reticuloendothelial organs is extensively required in maintaining arterial blood pressure, heart rate variability and modulate the innate and adaptive immune response (328). An increased proinflammatory cytokine storm is known to correspond to reduced VN activity in several inflammatory diseases, including rheumatoid arthritis (RA), systemic lupus erythematosus (SLE), sepsis, IBD (329–332). Activating the vagal efferent releases ACh at the distal end of the VN and effector ENS, inhibiting the release of proinflammatory cytokines (TNF-α, IL-1β, IL-6, and IL-18), forming the CAP (295, 333, 334). The CAP is a highly conserved pathway and plays an important role in controlling morbidity and mortality associated with various human diseases, such as endotoxemia, sepsis, IBD, and RA (293). VNS implants in patients with resistant epilepsy showed LPS-induced release of TNF-α, IL-1β, and IL-6 in post-VNS blood. Also, in the RA cohort, patients showed reduced disease severity with reduced TNF-α levels in peripheral blood samples (335, 336).

This pathway can aggressively target innate immune cells and proinflammatory cytokine production. Therefore, it is proposed as a potential therapeutic target for mitigating several infections, including sepsis and the cytokine storm recently reported in SARS-CoV-2 infection (337, 338). The neuronal circuits that control TNF-β production in macrophages and other innate cells in the spleen lack the enzymatic machinery for ACh production. Rosas-Ballina et al. identified an important connection of the inflammatory reflex by discovering the cholinergic machinery in the memory CD4^+^ T cells in the spleen (259, 339). Further, this lymphocyte-produced ACh regulates the innate immune response in the local tissue microenvironment (340). Given the importance of cholinergic signaling in inflammatory reflexes, several drugs and molecules originally designed for neurological diseases draw attention as potential drugs for inflammatory diseases. Some of the drugs that interfere with neuroimmune communication and affect inflammation and immunity are listed in **Tables 3** and **4**. Further, we discussed the notable cholinergic agents used in humans.

#### Cholinergic Agonists

Nicotine acts as a pan-agonist of various homomeric nAChRs including α7-nAChR, β2-nAChR, α3-nAChR, α4-nAChR, and α5-nAChR. However, in epidemiological and clinical trials, nicotine-induced addiction and toxicity leading to several autonomic dysfunctions, cardiovascular malfunctions, tumorigenesis, and neuropathic pain (341–344). Various selective cholinergic agonists are exploited in clinical research to reduce the adverse effects of non-selective receptor activation and cytokine dysregulation in various inflammatory conditions. Some of these selective agonists are discussed below-

##### GTS-21 [3-(2,4-dimethoxy-benzylidene) anabaseine]

GTS-21 (also known as DMBX-A) is an orally active small molecule and a selective α7-nAChR agonist used in clinical trials for AD and schizophrenia, shown to enhance memory and cognitive activity (345). GTS-21 is also known to attenuate the production of proinflammatory cytokines TNF-α and IL-1β from monocytes stimulated with Toll-like receptor (TLR) agonists (346). GTS-21 inhibits Akt and NF-κB signaling pathway, thereby reducing the LPS-induced cytokine production in macrophages (347). It has recently been shown that GTS-21 ameliorates polymicrobial sepsis-induced hepatic injury by modulating autophagy (348). GTS-21 is known to inhibit the differentiation of DCs and controls collagen-induced arthritis in mice (287). In human endotoxemia, GTS-21 induces an anti-inflammatory function (349), and higher GTS-21 concentration in the plasma significantly correlated with the lower amount of TNF-α, IL-6, and IL-1RA but not IL-10 (349, 350). It has been reported that chronic obstructive pulmonary disease (COPD) patients have high levels of IL-6 and nitric oxide (NO), and GTS-21 treatment suppresses the IL-6 and NO levels in plasma by modulating the function of PBMCs (351). In RA patients, GTS-21 suppresses the differentiation of Th1 cells and IFN-*γ* production in PBMCs (352). This drug has also displayed promising results in clinical trials for AD, schizophrenia, ameliorating disease severity in sepsis, pancreatitis, and inflammation induced by traumatic brain injury (353–355).

##### ABT-126

The α7-nAChR is an extensively studied cholinergic receptor for developing new drugs that will ameliorate cognitive deficiencies, neuropsychiatric disorders, inflammation, and autoimmune diseases. ABT-126 (trade name Neonicline) is a small molecule allosteric modulator of α7-nAChR. ABT-126 is a safe and well-tolerated α7-nAChR agonist. A phase II randomized controlled multi-center clinical trial showed a pro-cognitive effect in mild to moderate dementia AD patients (356). Phase II trials with ABT-126 also improved schizophrenia-associated cognitive impairment in non-smokers compared to smokers (357). A detailed study on the effect of ABT-21 on different immune parameters is yet to be studied. Given its importance, ABT-126 will be of great value in exploring an effective target for treating critical inflammatory and autoimmune diseases.

##### CNI-1493

CNI-1493 (also known as Semapimod) is an anti-amyloidogenic and vagal output stimulant that inhibits systemic inflammation *via* CAP (358). CNI-1493 was synthesized as an endogenous inhibitor of the synthesis of nitric oxide (NO) and inflammatory cytokines in the CNS (359, 360). In the pre-clinical AD model, CNI-1493 has a neuroprotective effect by inhibiting amyloid oligomers’ formation and subsequently suppressing the production of IL-6 and TNF-α (361). CNI-1493 has been shown to inhibit LPS-induced TNF-α, IL-1α, IL-1, IL-6, and IL-8 in macrophages and monocytes but not in T cells (362, 363). In acute bacterial infection, CNI-1493 has been reported to reduce the inflammatory response by inhibiting NO synthesis in macrophages and promoting ROS production in granulocytes (364). In the EAE model, CNI-1493 treatment has been shown to reduce DC maturation and T cell priming (365). CNI-1493 was also found to have a protective effect in clinical trials in gut inflammatory diseases like Crohn’s disease and pancreatitis (366, 367).

##### Pilocarpine

Pilocarpine is a natural alkaloid extracted from the plant *Pilocarpus.* It acts as a muscarinic agonist and is used to treat the autoimmune Sjogren’s syndrome. It stimulates saliva secretion, aqueous tears from lacrimal glands, and mucin from goblet cells (368, 369). Pilocarpine hydrochloride has been shown to inhibit *Candida albicans* biofilm formation and its pathogenicity (370). In the rat model of epilepticus seizure, intraperitoneal pilocarpine injection promotes activation of cholinergic neurons and dysregulation of brain homeostasis (371–373). It is known to have no severe side effects in humans as a parasympathomimetic drug (374). Its role in modulating immunological components in infection, cancer, and autoimmunity needs further investigation.

#### Acetylcholinesterase (AChE) inhibitors

Inhibitors of acetylcholinesterase (EC 3.1.1.7), such as galantamine, donepezil, huperzine, and rivastigmine, are some of the drugs approved for human use to treat AD, MS, and dementia (375). Most AChEIs are competitive inhibitors of AChE and allosteric modulators of nAChRs. During inflammation, the increased levels of ACh in the plasma cause these molecules to form a complex with exovesicular AChE, leading to increased nitric oxide efflux from endothelial cells (376). The AChE molecules can terminate activation of the cholinergic anti-inflammatory pathway on red blood cells (RBCs) surface (377). The AChE bound on RBCs’ surface can inactivate the plasma ACh and may enhance inflammation (376, 378). AChEI linked to the RBC membrane through a glycosylphosphatidylinositol (GPI) anchor also serves as an age marker for RBCs (377). AChEI modulates the anti-inflammatory pathway and helps in the release of ACh to compensate for the reduced number of AChRs in inflammatory and neurodegenerative diseases. Several AChEIs cross the blood-brain barrier and inhibit AChE and BChE in both the central and peripheral nervous systems. These are listed in **Table 3**. AChEIs are known to lower proinflammatory cytokines such as IFN-γ, IL-17, MCP-1, RANTES, TWEAK, and increase anti-inflammatory cytokines IL-4 and IL-10 (379).

#### Cholinergic Antagonists

Overexpression and altered parasympathetic inputs are often associated with the progression of ovarian, lung, skin cancers, and solid tumors (380, 381). Increased ACh signaling *via* M_1_ mAChR, M_2_ mAChR, and M_3_ mAChR also contributes to asthma and COPD (382). Abnormal cholinergic activity leads to immune-related pathological conditions in several skin diseases like atopic dermatitis, psoriasis, pemphigus, and palmoplantar pustulosis (19, 383, 384). Aberrant expression of ACh components, namely CHT1, ChAT, VAChT, nAChR, mAChR, and OCT1, and its release in GI tracts contribute to pathological conditions like IBD, colon cancer, and pancreatitis (70, 385, 386). Below, we discussed some of the promising antagonistic agents currently used in humans.

##### Mecamylamine

Mecamylamine (also known as inversine) is an orally available, non-selective, and non-competitive antagonist of heteromeric α4β2 and α3β4 subtype of nAChRs, and it can cross the blood-brain barrier even at a low dose (387). It is extensively used as an anti-hypertensive, anti-addictive, and anti-depressant drug (387). Mecamylamine is known to abolish the impairment of macrophages and decrease the *Mycobacterium tuberculosis* burden induced by nicotine (388). In the presence of IL‐18, mecamylamine abolishes the nicotine-induced inhibition of adhesion molecules on monocytes and cytokine production by PBMC (143). The potential for its effectiveness in treating neuroimmune diseases requires further investigation.

##### Atropine

It is widely used in treating bradycardia** **and inhibiting respiratory and oral secretion (389). *In vitro* treatment with atropine has been shown to reduce the production of IL-2 by concanavalin-A stimulated T cells and reduced the cytotoxicity of NK cells (390). Along with the suppression of T cell response, it significantly reduces tissue injury, leucocyte accumulation, and inflammatory reactions at the site of turpentine-induced inflammation (180). Atropine administration before LPS challenge in mice has been reported to reduce TNF-alpha and elevated IL-10 levels in the plasma, thus, having a protective role in endotoxic shock (233). Atropine is also shown to lower the IgA production in the small intestine of BALB/c mice (391). Currently, atropine is successfully used to treat myopia, IBD, and MG patients (392–394). However, its role in several neuroimmune and autoimmune diseases needs to be investigated.

##### Dicyclomine

Dicyclomine (also known as dicycloverine) is a selective M_1_ mAChR antagonist having an antispasmodic effect and is effectively used for treating several GI conditions such as irritable bowel syndrome (IBS) and intestinal cramping (395, 396). However, its exact mode of action in controlling mucosal homeostasis remains elusive. Apart from its antimuscarinic activities, it also has anti-fungal properties against the human pathogen *Candida albicans.* It prevents the growth, adhesion, biofilm formation, and yeast to hyphal transformation in *C. albicans* by targeting gene transduction in both the cAMP pathway and MAPK cascade (397). Dicyclomine hydrochloride is also found to have antibacterial potential in animals challenged with *Salmonella typhimurium via* competitive inhibition of lipase activity (398, 399). The anti-cholinergic implication of dicyclomine on immunological components requires further study.

##### Tiotropium

Tiotropium (trade names, Spiriva, Braltus) is a long-acting M_3_ mAChR antagonist that has an immunosuppressive function in allergic asthma and chronic obstructive pulmonary disease (COPD) and which improves airway remodeling (400). Tiotropium also reduces the Th2 cytokine production in mice (401), and antagonizes the LTB4 production in human alveolar macrophages (402). *In vitro* studies have demonstrated that the M_3_ mAChR expressed on macrophages is responsible for producing pro-inflammatory cytokines like IL-8 and leukotriene B4 (LTB-4). LTB-4 is an inflammatory mediator that causes leukocyte adhesion, activation, and inflammatory cell recruitments. M_3_ mAChR drives neutrophil recruitment *via* macrophage-derived chemotactic mediators (402). Tiotropium, an M_3_ mAChR antagonist, shows anti-chemotactic properties and reduces the ROS-mediated cytotoxicity in alveolar macrophages, thus reducing cellular inflammation (403, 404).

## Summary and Future Perspective

There is growing evidence suggesting bidirectional interactions between the nervous system and the immune system at the cellular and molecular levels. Understanding the multicellular and multidimensional signals involved and the regulatory mechanisms of immunological reflex in chronic and acute inflammatory diseases offer ample opportunity for basic and clinical research. Many neurodegenerative diseases have a close relationship with the activation of inflammation in the central nervous system and the peripheral immune system (405, 406). Given the importance of functional circuitry in the secondary lymphoid tissues (407), the cholinergic system’s influence on the immune system cannot be ignored while designing therapeutic strategies to treat even neurological disorders. In clinical trials (clinical trial registry numbers NCT00783068, NCT04470479, NCT00000172, NCT00892450), some cholinergic stimulators and pharmaceutical antagonists were used in various inflammatory diseases. These molecules can also alter the innate and adaptive response and need to be investigated further.

Further, the various activation mechanisms of the cholinergic system in different subsets of innate and adaptive immune cells need to be elucidated. A multidimensional and multifactorial systems biology approach could help connect various individual components, such as genetic disposition, cholinergic deficits, inflammatory mechanism, oxidative stress, mitochondrial dysfunction, and other neurotransmitter defects. Such an approach would serve well to understand neuro-immune diseases and may also help in customizing therapeutic regimens. The high degree of homology (64 to 68% sequence identity and 82 to 92% sequence similarity) between the transmembrane domains of mAChRs (250), makes designing small-molecule ligands that could selectively target specific mAChRs incredibly challenging. Recently, Biased M_1_ mAChR-mutant mice were used to develop next-generation drugs for AD, which hold promise for the future (408).

## Author Contributions

NH and GL conceived the idea and wrote the manuscript. All authors contributed to the article and approved the submitted version.

## Funding

NH received the Senior Research Fellowship from the Council of Scientific and Industrial Research. GL received grants from the Department of Biotechnology (Grants numbers, BT/PR15533/MED/30/1616/2015 and BT/PR14156/BRB/10/1515/2016) and Swarna Jayanti Fellowship (DST/SJF/LSA-01/2017-18) from Department of Science and Technology, Ministry of Science and Technology, Government of India.

## Conflict of Interest

The authors declare that the research was conducted in the absence of any commercial or financial relationships that could be construed as a potential conflict of interest.

## Abbreviations

α-BTX, α-bungarotoxin; ACh, Acetylcholine; AChE, AcetylcholinesteraseAChEI, Acetylcholinesterase inhibitor; AChRs, Acetylcholine receptor; AD, Alzheimer Disease; BBB, Blood-Brain Barrier; BChE, Butyrylcholinesterase; BChEI, Butyrylcholinesterase inhibitor; cAMP, Cyclase adenosine monophosphate; CD, Crohn’s disease; ChAT, Choline acetyltransferase; ChE, cholinesterase; ChEI, cholinesterase inhibitor; ChT, Choline transporter; CIA, Collagen-induced arthritis; CNS, Central nervous system; COPD, Chronic obstructive pulmonary disease; CS, Cholinergic status; CTL1, Choline transporter-like protein 1; CTLA-4, Cytotoxic T lymphocyte-associated protein 4; EAE, Experimental autoimmune encephalomyelitis; ENS, Enteric nervous system; FOXP3: Forkhead box P3; GI, Gastrointestinal; GPCRs, G protein-coupled receptors; HPA, Hypothalamic–pituitary–adrenal axis; IBD, Inflammatory bowel disease; IBS, Irritable bowel syndrome; ILs, Interleukins; JAK-STAT, Janus kinases signal transducer and activator of transcription; LTB-4, Leukotriene B4; MAPK, Mitogen-activated protein kinase; mAChRs, Muscarinic acetylcholine receptor; MG, Myasthenia Gravis; MS, Multiple sclerosis; nAChRs, Nicotinic acetylcholine receptor; NICUs, neuro-immune cell units; NK cells, Natural killer cells; NMJ, Neuromuscular junction; NNCS, Non-neuronal cholinergic system; NO, nitric oxide; OXO-M, Oxotremorine; PBMCs, Peripheral blood mononuclear cells; PHA, phytohemagglutinin; PKC, protein kinase C; PLC, Phospholipase C; RA, Rheumatoid arthritis; SLE, Systemic lupus erythematosus; VAChT; Vesicular acetylcholine transporter; VN, Vagus nerve; VNS, Vagus nerve stimulation; WT, wild-type.

## References

[1] Karmakar S Lal G. Role of serotonin receptor signaling in cancer cells and anti-tumor immunity. Theranostics (2021) 11(11):5296–312. 10.7150/thno.55986 PMC8039959 33859748

[2] Pavlov VA Tracey KJ. The vagus nerve and the inflammatory reflex–linking immunity and metabolism. Nat Rev Endocrinol (2012) 8(12):743–54. 10.1038/nrendo.2012.189 PMC4082307 23169440

[3] Berthoud HR Neuhuber WL. Functional and chemical anatomy of the afferent vagal system. Auton Neurosci (2000) 85(1-3):1–17. 10.1016/S1566-0702(00)00215-0 11189015

[4] Wessler I Kirkpatrick CJ. Cholinergic signaling controls immune functions and promotes homeostasis. Int Immunopharmacol (2020) 83:106345. 10.1016/j.intimp.2020.106345 32203906

[5] Kawashima K Fujii T. Basic and clinical aspects of non-neuronal acetylcholine: overview of non-neuronal cholinergic systems and their biological significance. J Pharmacol Sci (2008) 106(2):167–73. 10.1254/jphs.fm0070073 18285657

[6] Dani JA Bertrand D. Nicotinic acetylcholine receptors and nicotinic cholinergic mechanisms of the central nervous system. Annu Rev Pharmacol Toxicol (2007) 47:699–729. 10.1146/annurev.pharmtox.47.120505.105214 17009926

[7] Papke RL Lindstrom JM. Nicotinic acetylcholine receptors: Conventional and unconventional ligands and signaling. Neuropharmacology (2020) 168:108021. 10.1016/j.neuropharm.2020.108021 32146229 PMC7610230

[8] Prado VF Janickova H Al-Onaizi MA Prado MA. Cholinergic circuits in cognitive flexibility. Neuroscience (2017) 345:130–41. 10.1016/j.neuroscience.2016.09.013 27641830

[9] Sanders LM Zeisel SH. Choline: Dietary Requirements and Role in Brain Development. Nutr Today (2007) 42(4):181–6. 10.1097/01.NT.0000286155.55343.fa PMC2518394 18716669

[10] Papatriantafyllou M. Neuroimmunology: ChATty B cells. Nat Rev Immunol (2013) 13(2):70. 10.1038/nri3396 23348411

[11] Wessler I Kirkpatrick CJ Racke K. The cholinergic ‘pitfall’: acetylcholine, a universal cell molecule in biological systems, including humans. Clin Exp Pharmacol Physiol (1999) 26(3):198–205. 10.1046/j.1440-1681.1999.03016.x 10081614

[12] Kawashima K Fujii T. Extraneuronal cholinergic system in lymphocytes. Pharmacol Ther (2000) 86(1):29–48. 10.1016/S0163-7258(99)00071-6 10760545

[13] Stanaszek PM Snell JF O’Neill JJ. Isolation, extraction, and measurement of acetylcholine from Lactobacillus plantarum. Appl Environ Microbiol (1977) 34(2):237–9. 10.1128/AEM.34.2.237-239.1977 PMC242629 907345

[14] Reale M de Angelis F di Nicola M Capello E di Ioia M Luca G. Relation between pro-inflammatory cytokines and acetylcholine levels in relapsing-remitting multiple sclerosis patients. Int J Mol Sci (2012) 13(10):12656–64. 10.3390/ijms131012656 PMC3497293 23202919

[15] Lombardo S Maskos U. Role of the nicotinic acetylcholine receptor in Alzheimer’s disease pathology and treatment. Neuropharmacology (2015) 96(Pt B):255–62. 10.1016/j.neuropharm.2014.11.018 25514383

[16] Jia JP Jia JM Zhou WD Xu M Chu CB Yan X. Differential acetylcholine and choline concentrations in the cerebrospinal fluid of patients with Alzheimer’s disease and vascular dementia. Chin Med J (Engl) (2004) 117(8):1161–4. 15361288

[17] Profita M Albano GD Riccobono L Di Sano C Montalbano AM Gagliardo R. Increased levels of Th17 cells are associated with non-neuronal acetylcholine in COPD patients. Immunobiology (2014) 219(5):392–401. 10.1016/j.imbio.2014.01.004 24529390

[18] Apatzidou DA Iskas A Konstantinidis A Alghamdi AM Tumelty M Lappin DF. Clinical associations between acetylcholine levels and cholinesterase activity in saliva and gingival crevicular fluid and periodontal diseases. J Clin Periodontol (2018) 45(10):1173–83. 10.1111/jcpe.12989 30022504

[19] Wessler I Reinheimer T Kilbinger H Bittinger F Kirkpatrick CJ Saloga J. Increased acetylcholine levels in skin biopsies of patients with atopic dermatitis. Life Sci (2003) 72(18-19):2169–72. 10.1016/s0024-3205(03)00079-1 12628475

[20] Yuan M Han B Xia Y Liu Y Wang C Zhang C. Augmentation of peripheral lymphocyte-derived cholinergic activity in patients with acute ischemic stroke. BMC Neurol (2019) 19(1):236. 10.1186/s12883-019-1481-5 31615442 PMC6792255

[21] Fujii T Mashimo M Moriwaki Y Misawa H Ono S Horiguchi K. Expression and Function of the Cholinergic System in Immune Cells. Front Immunol (2017) 8:1085:1085. 10.3389/fimmu.2017.01085 28932225 PMC5592202

[22] Bellier JP Kimura H. Peripheral type of choline acetyltransferase: biological and evolutionary implications for novel mechanisms in cholinergic system. J Chem Neuroanat (2011) 42(4):225–35. 10.1016/j.jchemneu.2011.02.005 21382474

[23] Oda Y. Choline acetyltransferase: the structure, distribution and pathologic changes in the central nervous system. Pathol Int (1999) 49(11):921–37. 10.1046/j.1440-1827.1999.00977.x 10594838

[24] Ogawa H Fujii T Watanabe Y Kawashima K. Expression of multiple mRNA species for choline acetyltransferase in human T-lymphocytes. Life Sci (2003) 72(18-19):2127–30. 10.1016/s0024-3205(03)00072-9 12628468

[25] Misawa H Matsuura J Oda Y Takahashi R Deguchi T. Human choline acetyltransferase mRNAs with different 5’-region produce a 69-kDa major translation product. Brain Res Mol Brain Res (1997) 44(2):323–33. 10.1016/s0169-328x(96)00231-8 9073174

[26] Salamone G Lombardi G Gori S Nahmod K Jancic C Amaral MM. Cholinergic modulation of dendritic cell function. J Neuroimmunol (2011) 236(1-2):47–56. 10.1016/j.jneuroim.2011.05.007 21665296

[27] Koarai A Traves SL Fenwick PS Brown SM Chana KK Russell RE. Expression of muscarinic receptors by human macrophages. Eur Respir J (2012) 39(3):698–704. 10.1183/09031936.00136710 21885397

[28] Cox MA Duncan GS Lin GHY Steinberg BE Yu LX Brenner D. Choline acetyltransferase-expressing T cells are required to control chronic viral infection. Science (6427) 2019) 363:639–44. 10.1126/science.aau9072 PMC7181845 30733420

[29] Tayebati SK El-Assouad D Ricci A Amenta F. Immunochemical and immunocytochemical characterization of cholinergic markers in human peripheral blood lymphocytes. J Neuroimmunol (2002) 132(1-2):147–55. 10.1016/s0165-5728(02)00325-9 12417445

[30] Milara J Cervera A de Diego A Sanz C Juan G Gavaldà A. Non-neuronal cholinergic system contributes to corticosteroid resistance in chronic obstructive pulmonary disease patients. Respir Res (2016) 17(1):145. 10.1186/s12931-016-0467-8 27825347 PMC5101693

[31] Jönsson M Norrgård O Forsgren S. Presence of a marked nonneuronal cholinergic system in human colon: study of normal colon and colon in ulcerative colitis. Inflammation Bowel Dis (2007) 13(11):1347–56. 10.1002/ibd.20224 17663429

[32] Kaufman H Vadasz C Lajtha A. Effects of estradiol and dexamethasone on choline acetyltransferase activity in various rat brain regions. Brain Res (1988) 453(1-2):389–92. 10.1016/0006-8993(88)90185-0 3401777

[33] Luine VN. Estradiol increases choline acetyltransferase activity in specific basal forebrain nuclei and projection areas of female rats. Exp Neurol (1985) 89(2):484–90. 10.1016/0014-4886(85)90108-6 2990988

[34] Pierdominici M Maselli A Colasanti T Giammarioli AM Delunardo F Vacirca D. Estrogen receptor profiles in human peripheral blood lymphocytes. Immunol Lett (2010) 132(1-2):79–85. 10.1016/j.imlet.2010.06.003 20542061

[35] Beagley KW Gockel CM. Regulation of innate and adaptive immunity by the female sex hormones oestradiol and progesterone. FEMS Immunol Med Microbiol (2003) 38(1):13–22. 10.1016/S0928-8244(03)00202-5 12900050

[36] Schneider AH Kanashiro A Dutra SGV Souza RDN Veras FP Cunha FQ. Estradiol replacement therapy regulates innate immune response in ovariectomized arthritic mice. Int Immunopharmacol (2019) 72:504–10. 10.1016/j.intimp.2019.04.048 PMC7023895 31055232

[37] Rothenberger NJ Somasundaram A Stabile LP. The Role of the Estrogen Pathway in the Tumor Microenvironment. Int J Mol Sci (2018) 19(2):611. 10.3390/ijms19020611 PMC5855833 29463044

[38] Maglione A Rolla S Mercanti SF Cutrupi S Clerico M. The Adaptive Immune System in Multiple Sclerosis: An Estrogen-Mediated Point of View. Cells (2019) 8(10):1280. 10.3390/cells8101280 PMC6829884 31635066

[39] Cannon RL Hoover DB Baisden RH Woodruff ML. Effects of trimethyltin (TMT) on choline acetyltransferase activity in the rat hippocampus. Influence of dose and time following exposure. Mol Chem Neuropathol (1994) 23(1):27–45. 10.1007/BF02858505 7893329

[40] Hioe KM Jones JM. Effects of trimethyltin on the immune system of rats. Toxicol Lett (1984) 20(3):317–23. 10.1016/0378-4274(84)90166-8 6701918

[41] Holloway LN Pannell KH Whalen MM. Effects of a series of triorganotins on ATP levels in human natural killer cells. Environ Toxicol Pharmacol (2008) 25(1):43–50. 10.1016/j.etap.2007.08.008 19122738 PMC2245884

[42] Röhl C Grell M Maser E. The organotin compounds trimethyltin (TMT) and triethyltin (TET) but not tributyltin (TBT) induce activation of microglia co-cultivated with astrocytes. Toxicol In Vitro (2009) 23(8):1541–7. 10.1016/j.tiv.2009.04.013 19422909

[43] Seo YS Ang MJ Moon BC Kim HS Choi G Lim HS. Protective Effects of. Brain Sci (2019) 9(12):369. 10.3390/brainsci9120369 PMC6955677 31842431

[44] Pompili E Fabrizi C Fumagalli L Fornai F. Autophagy in trimethyltin-induced neurodegeneration. J Neural Transm (Vienna) (2020) 127(7):987–98. 10.1007/s00702-020-02210-1 32451631

[45] Clevers HC Hoeksema M Gmelig-Meyling FH Ballieux RE. Calcium ionophore A23187 induces interleukin 2 reactivity in human T cells. Scand J Immunol (1985) 22(6):633–8. 10.1111/j.1365-3083.1985.tb01925.x 3937226

[46] Peng WB Sha WH Li YY Nie YQ. In vitro anti-tumor effect of cytotoxic T lymphocyte activated by antigen- loaded dendritic cells from peripheral blood mononuclear cells treated with calcium ionophore A23187 and GM-CSF. Zhonghua Yi Xue Za Zhi (2010) 90(26):1849–53. 20979834

[47] Boot JH Van Hilten JA. The use of the divalent calcium-ionophore A23187 as a biochemical tool in pharmacological and in vitro toxicological studies. Cell Struct Funct (1996) 21(2):97–9. 10.1247/csf.21.97 8790938

[48] Fujii T Ushiyama N Hosonuma K Suenaga A Kawashima K. Effects of human antithymocyte globulin on acetylcholine synthesis, its release and choline acetyltransferase transcription in a human leukemic T-cell line. J Neuroimmunol (2002) 128(1-2):1–8. 10.1016/s0165-5728(02)00111-x 12098504

[49] Roider T Katzfuß M Matos C Singer K Renner K Oefner PJ. Antithymocyte Globulin Induces a Tolerogenic Phenotype in Human Dendritic Cells. Int J Mol Sci (2016) 17(12):2081. 10.3390/ijms17122081 PMC5187881 27973435

[50] Duftner C Dejaco C Hengster P Bijuklic K Joannidis M Margreiter R. Apoptotic effects of antilymphocyte globulins on human pro-inflammatory CD4+CD28- T-cells. PloS One (2012) 7(3):e33939. 10.1371/journal.pone.0033939 22479483 PMC3316508

[51] Gharekhani A Entezari-Maleki T Dashti-Khavidaki S Khalili H. A review on comparing two commonly used rabbit anti-thymocyte globulins as induction therapy in solid organ transplantation. Expert Opin Biol Ther (2013) 13(9):1299–313. 10.1517/14712598.2013.822064 23875884

[52] Fujii T Takada-Takatori Y Kawashima K. Regulatory mechanisms of acetylcholine synthesis and release by T cells. Life Sci (2012) 91(21-22):981–5. 10.1016/j.lfs.2012.04.031 22569292

[53] Jun H Yu H Gong J Jiang J Qiao X Perkey E. An immune-beige adipocyte communication via nicotinic acetylcholine receptor signaling. Nat Med (2018) 24(6):814–22. 10.1038/s41591-018-0032-8 PMC5992032 29785025

[54] Cook-Mills JM Mokyr MB Cohen RL Perlman RL Chambers DA. Neurotransmitter suppression of the in vitro generation of a cytotoxic T lymphocyte response against the syngeneic MOPC-315 plasmacytoma. Cancer Immunol Immunother (1995) 40(2):79–87. 10.1007/BF01520288 7882386 PMC11037700

[55] Gilbert KM Hoffmann MK. cAMP is an essential signal in the induction of antibody production by B cells but inhibits helper function of T cells. J Immunol (1985) 135(3):2084–9. 2991378

[56] Ikeda C Morita I Mori A Fujimoto K Suzuki T Kawashima K. Phorbol ester stimulates acetylcholine synthesis in cultured endothelial cells isolated from porcine cerebral microvessels. Brain Res (1994) 655(1-2):147–52. 10.1016/0006-8993(94)91608-X 7529125

[57] Chalimoniuk M King-Pospisil K Pedersen WA Malecki A Wylegala E Mattson MP. Arachidonic acid increases choline acetyltransferase activity in spinal cord neurons through a protein kinase C-mediated mechanism. J Neurochem (2004) 90(3):629–36. 10.1111/j.1471-4159.2004.02535.x 15255940

[58] Kawashima K Fujii T. Expression of non-neuronal acetylcholine in lymphocytes and its contribution to the regulation of immune function. Front Biosci (2004) 9:2063–85. 10.2741/1390 15353271

[59] Fujii T Yamada S Watanabe Y Misawa H Tajima S Fujimoto K. Induction of choline acetyltransferase mRNA in human mononuclear leukocytes stimulated by phytohemagglutinin, a T-cell activator. J Neuroimmunol (1998) 82(1):101–7. 10.1016/S0165-5728(97)00195-1 9526852

[60] Graham KL Zhang JV Lewén S Burke TM Dang T Zoudilova M. A novel CMKLR1 small molecule antagonist suppresses CNS autoimmune inflammatory disease. PloS One (2014) 9(12):e112925. 10.1371/journal.pone.0112925 25437209 PMC4249827

[61] Fujii T Tsuchiya T Yamada S Fujimoto K Suzuki T Kasahara T. Localization and synthesis of acetylcholine in human leukemic T cell lines. J Neurosci Res (1996) 44(1):66–72. 10.1002/(SICI)1097-4547(19960401)44:1<66::AID-JNR9>3.0.CO;2-G 8926632

[62] Imai T Tsuda E Hozuki T Yamauchi R Saitoh M Hisahara S. Early effect of tacrolimus in improving excitation-contraction coupling in myasthenia gravis. Clin Neurophysiol (2012) 123(9):1886–90. 10.1016/j.clinph.2012.01.017 22386321

[63] Sharma K. Cholinesterase inhibitors as Alzheimer’s therapeutics (Review). Mol Med Rep (2019) 20(2):1479–87. 10.3892/mmr.2019.10374 PMC6625431 31257471

[64] Darvesh S Hopkins DA Geula C. Neurobiology of butyrylcholinesterase. Nat Rev Neurosci (2003) 4(2):131–8. 10.1038/nrn1035 12563284

[65] Mason HJ. The recovery of plasma cholinesterase and erythrocyte acetylcholinesterase activity in workers after over-exposure to dichlorvos. Occup Med (Lond) (2000) 50(5):343–7. 10.1093/occmed/50.5.343 10975133

[66] Kasprzak H Salpeter MM. Recovery of acetylcholinesterase at intact neuromuscular junctions after in vivo inactivation with di-isopropylfluorophosphate. J Neurosci (1985) 5(4):951–5. 10.1523/JNEUROSCI.05-04-00951.1985 PMC6564997 3981251

[67] Grisaru D Sternfeld M Eldor A Glick D Soreq H. Structural roles of acetylcholinesterase variants in biology and pathology. Eur J Biochem (1999) 264(3):672–86. 10.1046/j.1432-1327.1999.00693.x 10491113

[68] Szelenyi J Paldi-Haris P Hollan S. Changes in the cholinergic system of lymphocytes due to mitogenic stimulation. Immunol Lett (1987) 16(1):49–54. 10.1016/0165-2478(87)90060-5 3480875

[69] Hod K Sperber AD Maharshak N Ron Y Shapira I David Z. Serum cholinesterase activity is elevated in female diarrhea-predominant irritable bowel syndrome patients compared to matched controls. Neurogastroenterol Motil (2018) 30(12):e13464. 10.1111/nmo.13464 30240124

[70] Maharshak N Shenhar-Tsarfaty S Aroyo N Orpaz N Guberman I Canaani J. MicroRNA-132 modulates cholinergic signaling and inflammation in human inflammatory bowel disease. Inflammation Bowel Dis (2013) 19(7):1346–53. 10.1097/MIB.0b013e318281f47d 23598815

[71] García-Ayllón MS Millán C Serra-Basante C Bataller R Sáez-Valero J. Readthrough acetylcholinesterase is increased in human liver cirrhosis. PloS One (2012) 7(9):e44598. 10.1371/journal.pone.0044598 23028565 PMC3441564

[72] García-Ayllón MS Riba-Llena I Serra-Basante C Alom J Boopathy R Sáez-Valero J. Altered levels of acetylcholinesterase in Alzheimer plasma. PloS One (2010) 5(1):e8701. 10.1371/journal.pone.0008701 20090844 PMC2806824

[73] Darvesh S Leblanc AM Macdonald IR Reid GA Bhan V Macaulay RJ. Butyrylcholinesterase activity in multiple sclerosis neuropathology. Chem Biol Interact (2010) 187(1-3):425–31. 10.1016/j.cbi.2010.01.037 20122907

[74] Brenner T Hamra-Amitay Y Evron T Boneva N Seidman S Soreq H. The role of readthrough acetylcholinesterase in the pathophysiology of myasthenia gravis. FASEB J (2003) 17(2):214–22. 10.1096/fj.02-0609com 12554700

[75] Rogers SL Friedhoff LT. The efficacy and safety of donepezil in patients with Alzheimer’s disease: results of a US Multicentre, Randomized, Double-Blind, Placebo-Controlled Trial. The Donepezil Study Group. Dementia (1996) 7(6):293–303. 10.1159/000106895 8915035

[76] Bar-On P Millard CB Harel M Dvir H Enz A Sussman JL. Kinetic and structural studies on the interaction of cholinesterases with the anti-Alzheimer drug rivastigmine. Biochemistry (2002) 41(11):3555–64. 10.1021/bi020016x 11888271

[77] Gowayed MA Rothe K Rossol M Attia AS Wagner U Baerwald C. The role of α7nAChR in controlling the anti-inflammatory/anti-arthritic action of galantamine. Biochem Pharmacol (2019) 170:113665. 10.1016/j.bcp.2019.113665 31606410

[78] Takata K Kitamura Y Saeki M Terada M Kagitani S Kitamura R. Galantamine-induced amyloid-{beta} clearance mediated via stimulation of microglial nicotinic acetylcholine receptors. J Biol Chem (2010) 285(51):40180–91. 10.1074/jbc.M110.142356 PMC3001000 20947502

[79] Ji H Rabbi MF Labis B Pavlov VA Tracey KJ Ghia JE. Central cholinergic activation of a vagus nerve-to-spleen circuit alleviates experimental colitis. Mucosal Immunol (2014) 7(2):335–47. 10.1038/mi.2013.52 PMC3859808 23881354

[80] Raskind MA Peskind ER Wessel T Yuan W. Galantamine in AD: A 6-month randomized, placebo-controlled trial with a 6-month extension. The Galantamine USA-1 Study Group. Neurology (2000) 54(12):2261–8. 10.1212/wnl.54.12.2261 10881250

[81] Shifrin H Nadler-Milbauer M Shoham S Weinstock M. Rivastigmine alleviates experimentally induced colitis in mice and rats by acting at central and peripheral sites to modulate immune responses. PloS One (2013) 8(2):e57668. 10.1371/journal.pone.0057668 23469045 PMC3585220

[82] Nizri E Irony-Tur-Sinai M Faranesh N Lavon I Lavi E Weinstock M. Suppression of neuroinflammation and immunomodulation by the acetylcholinesterase inhibitor rivastigmine. J Neuroimmunol (2008) 203(1):12–22. 10.1016/j.jneuroim.2008.06.018 18692909

[83] Emre M Aarsland D Albanese A Byrne EJ Deuschl G De Deyn PP. Rivastigmine for dementia associated with Parkinson’s disease. N Engl J Med (2004) 351(24):2509–18. 10.1056/NEJMoa041470 15590953

[84] Rösler M Anand R Cicin-Sain A Gauthier S Agid Y Dal-Bianco P. Efficacy and safety of rivastigmine in patients with Alzheimer’s disease: international randomised controlled trial. BMJ (1999) 318(7184):633–8. 10.1136/bmj.318.7184.633 PMC27767 10066203

[85] Huang W Zhu S Liu X Huang L Han Y Han Q. Cholinergic anti-inflammatory pathway involves in the neuroprotective effect of huperzine A on sepsis-associated encephalopathy. Zhonghua Wei Zhong Bing Ji Jiu Yi Xue (2016) 28(5):450–4. 29920043

[86] Damar U Gersner R Johnstone JT Schachter S Rotenberg A. Huperzine A as a neuroprotective and antiepileptic drug: a review of preclinical research. Expert Rev Neurother (2016) 16(6):671–80. 10.1080/14737175.2016.1175303 27086593

[87] Desilets AR Gickas JJ Dunican KC. Role of huperzine a in the treatment of Alzheimer’s disease. Ann Pharmacother (2009) 43(3):514–8. 10.1345/aph.1L402 19240260

[88] Parthasarathy G Ravi K Camilleri M Andrews C Szarka LA Low PA. Effect of neostigmine on gastroduodenal motility in patients with suspected gastrointestinal motility disorders. Neurogastroenterol Motil (2015) 27(12):1736–46. 10.1111/nmo.12669 PMC4659742 26387781

[89] Frankel A Gillespie C Lu CT Hewett P Wattchow D. Subcutaneous neostigmine appears safe and effective for acute colonic pseudo-obstruction (Ogilvie’s syndrome). ANZ J Surg (2019) 89(6):700–5. 10.1111/ans.15265 31083785

[90] Valdes-Ferrer SI Crispin JC Belaunzaran PF Cantu-Brito CG Sierra-Madero J Alcocer-Varela J. Acetylcholine-esterase inhibitor pyridostigmine decreases T cell overactivation in patients infected by HIV. AIDS Res Hum Retroviruses (2009) 25(8):749–55. 10.1089/aid.2008.0257 19645607

[91] Singh SP Chand HS Banerjee S Agarwal H Raizada V Roy S. Acetylcholinesterase Inhibitor Pyridostigmine Bromide Attenuates Gut Pathology and Bacterial Dysbiosis in a Murine Model of Ulcerative Colitis. Dig Dis Sci (2020) 65(1):141–9. 10.1007/s10620-019-05838-6 PMC6943409 31643033

[92] Rocha JA Ribeiro SP França CM Coelho O Alves G Lacchini S. Increase in cholinergic modulation with pyridostigmine induces anti-inflammatory cell recruitment soon after acute myocardial infarction in rats. Am J Physiol Regul Integr Comp Physiol (2016) 310(8):R697–706. 10.1152/ajpregu.00328.2015 PMC4867407 26791829

[93] Maggi L Mantegazza R. Treatment of myasthenia gravis: focus on pyridostigmine. Clin Drug Investig (2011) 31(10):691–701. 10.2165/11593300-000000000-00000 21815707

[94] Manini ML Camilleri M Grothe R Di Lorenzo C. Application of Pyridostigmine in Pediatric Gastrointestinal Motility Disorders: A Case Series. Paediatr Drugs (2018) 20(2):173–80. 10.1007/s40272-017-0277-6 29243034

[95] Kalb A von Haefen C Sifringer M Tegethoff A Paeschke N Kostova M. Acetylcholinesterase inhibitors reduce neuroinflammation and -degeneration in the cortex and hippocampus of a surgery stress rat model. PloS One (2013) 8(5):e62679. 10.1371/journal.pone.0062679 23671623 PMC3643957

[96] Arens AM Kearney T. Adverse Effects of Physostigmine. J Med Toxicol (2019) 15(3):184–91. 10.1007/s13181-019-00697-z PMC6597673 30747326

[97] Agatonovic-Kustrin S Kettle C Morton DW. A molecular approach in drug development for Alzheimer’s disease. BioMed Pharmacother (2018) 106:553–65. 10.1016/j.biopha.2018.06.147 29990843

[98] Matsueda K Hongo M Tack J Aoki H Saito Y Kato H. Clinical trial: dose-dependent therapeutic efficacy of acotiamide hydrochloride (Z-338) in patients with functional dyspepsia - 100 mg t.i.d. is an optimal dosage. Neurogastroenterol Motil (2010) 22(6):618–e173. 10.1111/j.1365-2982.2009.01449.x 20059698

[99] StatPearls. (2021).

[100] Maroli A Di Lascio S Drufuca L Cardani S Setten E Locati M. Effect of donepezil on the expression and responsiveness to LPS of CHRNA7 and CHRFAM7A in macrophages: A possible link to the cholinergic anti-inflammatory pathway. J Neuroimmunol (2019) 332:155–66. 10.1016/j.jneuroim.2019.04.012 31048268

[101] Jiang Y Zou Y Chen S Zhu C Wu A Liu Y. The anti-inflammatory effect of donepezil on experimental autoimmune encephalomyelitis in C57 BL/6 mice. Neuropharmacology (2013) 73:415–24. 10.1016/j.neuropharm.2013.06.023 23831366

[102] Karu TI Riabykh TP Sidorova TA Dobrynin IV. Comparison of blast cell sensitivity to low-intensity laser radiation and chemotherapeutic drugs. Dokl Akad Nauk (1997) 353(1):114–7. 9273047

[103] Arikawa M Kakinuma Y Noguchi T Todaka H Sato T. Donepezil, an acetylcholinesterase inhibitor, attenuates LPS-induced inflammatory response in murine macrophage cell line RAW 264.7 through inhibition of nuclear factor kappa B translocation. Eur J Pharmacol (2016) 789:17–26. 10.1016/j.ejphar.2016.06.053 27373848

[104] Sato T Enoki Y Sakamoto Y Yokota K Okubo M Matsumoto M. Donepezil prevents RANK-induced bone loss via inhibition of osteoclast differentiation by downregulating acetylcholinesterase. Heliyon (2015) 1(1):e00013. 10.1016/j.heliyon.2015.e00013 27441211 PMC4939821

[105] Lee JH Jeong SK Kim BC Park KW Dash A. Donepezil across the spectrum of Alzheimer’s disease: dose optimization and clinical relevance. Acta Neurol Scand (2015) 131(5):259–67. 10.1111/ane.12386 25690270

[106] Tuğal O Yazici KM Anil Yağcioğlu AE Göğüş A. A double-blind, placebo controlled, cross-over trial of adjunctive donepezil for cognitive impairment in schizophrenia. Int J Neuropsychopharmacol (2004) 7(2):117–23. 10.1017/S1461145703004024 14741060

[107] Muccioli G Raso GM Ghe C Di Carlo R. Effect of L-alpha glycerylphosphorylcholine on muscarinic receptors and membrane microviscosity of aged rat brain. Prog Neuropsychopharmacol Biol Psychiatry (1996) 20(2):323–39. 10.1016/0278-5846(95)00313-4 8861196

[108] Saegusa Y Takeda H Muto S Oridate N Nakagawa K Sadakane C. Decreased motility of the lower esophageal sphincter in a rat model of gastroesophageal reflux disease may be mediated by reductions of serotonin and acetylcholine signaling. Biol Pharm Bull (2011) 34(5):704–11. 10.1248/bpb.34.704 21532161

[109] Dou Y Luo J Wu X Wei Z Tong B Yu J. Curcumin attenuates collagen-induced inflammatory response through the “gut-brain axis”. J Neuroinflamm (2018) 15(1):6. 10.1186/s12974-017-1047-7 PMC5756354 29306322

[110] Peeyush Kumar T Antony S Soman S Kuruvilla KP George N Paulose CS. Role of curcumin in the prevention of cholinergic mediated cortical dysfunctions in streptozotocin-induced diabetic rats. Mol Cell Endocrinol (2011) 331(1):1–10. 10.1016/j.mce.2010.07.004 20637830

[111] Okuda T Haga T Kanai Y Endou H Ishihara T Katsura I. Identification and characterization of the high-affinity choline transporter. Nat Neurosci (2000) 3(2):120–5. 10.1038/72059 10649566

[112] Iwao B Yara M Hara N Kawai Y Yamanaka T Nishihara H. Functional expression of choline transporter like-protein 1 (CTL1) and CTL2 in human brain microvascular endothelial cells. Neurochem Int (2016) 93:40–50. 10.1016/j.neuint.2015.12.011 26746385

[113] Fujii T Okuda T Haga T Kawashima K. Detection of the high-affinity choline transporter in the MOLT-3 human leukemic T-cell line. Life Sci (2003) 72(18-19):2131–4. 10.1016/S0024-3205(03)00073-0 12628469

[114] Snider SA Margison KD Ghorbani P LeBlond ND O’Dwyer C Nunes JRC. Choline transport links macrophage phospholipid metabolism and inflammation. J Biol Chem (2018) 293(29):11600–11. 10.1074/jbc.RA118.003180 PMC6065184 29880645

[115] Varoqui H Erickson JD. Active transport of acetylcholine by the human vesicular acetylcholine transporter. J Biol Chem (1996) 271(44):27229–32. 10.1074/jbc.271.44.27229 8910293

[116] Weihe E Tao-Cheng JH Schäfer MK Erickson JD Eiden LE. Visualization of the vesicular acetylcholine transporter in cholinergic nerve terminals and its targeting to a specific population of small synaptic vesicles. Proc Natl Acad Sci USA (1996) 93(8):3547–52. 10.1073/pnas.93.8.3547 PMC39647 8622973

[117] Hoover DB Poston MD Brown S Lawson SE Bond CE Downs AM. Cholinergic leukocytes in sepsis and at the neuroimmune junction in the spleen. Int Immunopharmacol (2020) 81:106359. 10.1016/j.intimp.2020.106359 32143148 PMC7315439

[118] Wu XQ Zhao YN Ding J Si Z Cheng DF Shi HC. Decreased vesicular acetylcholine transporter related to memory deficits in epilepsy: A. Epilepsia (2018) 59(9):1655–66. 10.1111/epi.14533 30126014

[119] Efange SM Garland EM Staley JK Khare AB Mash DC. Vesicular acetylcholine transporter density and Alzheimer’s disease. Neurobiol Aging (1997) 18(4):407–13. 10.1016/s0197-4580(97)00038-9 9330972

[120] Leite HR Oliveira-Lima OC Pereira LM Oliveira VEM Prado VF Prado MAM. Vesicular acetylcholine transporter knock down-mice are more susceptible to inflammation, c-Fos expression and sickness behavior induced by lipopolysaccharide. Brain Behav Immun (2016) 57:282–92. 10.1016/j.bbi.2016.05.005 27179819

[121] Elwary SM Chavan B Schallreuter KU. The vesicular acetylcholine transporter is present in melanocytes and keratinocytes in the human epidermis. J Invest Dermatol (2006) 126(8):1879–84. 10.1038/sj.jid.5700268 16763548

[122] Rodriguez-Diaz R Dando R Jacques-Silva MC Fachado A Molina J Abdulreda MH. Alpha cells secrete acetylcholine as a non-neuronal paracrine signal priming beta cell function in humans. Nat Med (2011) 17(7):888–92. 10.1038/nm.2371 PMC3132226 21685896

[123] Sopori ML Kozak W Savage SM Geng Y Soszynski D Kluger MJ. Effect of nicotine on the immune system: possible regulation of immune responses by central and peripheral mechanisms. Psychoneuroendocrinology (1998) 23(2):189–204. 10.1016/S0306-4530(97)00076-0 9621398

[124] Itier V Bertrand D. Neuronal nicotinic receptors: from protein structure to function. FEBS Lett (2001) 504(3):118–25. 10.1016/S0014-5793(01)02702-8 11532443

[125] Gharpure A Teng J Zhuang Y Noviello CM Walsh RM Jr Cabuco R. Agonist Selectivity and Ion Permeation in the alpha3beta4 Ganglionic Nicotinic Receptor. Neuron (2019) 104(3):501–11.e6. 10.1016/j.neuron.2019.07.030 31488329 PMC6842111

[126] Gotti C Clementi F Fornari A Gaimarri A Guiducci S Manfredi I. Structural and functional diversity of native brain neuronal nicotinic receptors. Biochem Pharmacol (2009) 78(7):703–11. 10.1016/j.bcp.2009.05.024 19481063

[127] Tomizawa M Maltby D Talley TT Durkin KA Medzihradszky KF Burlingame AL. Atypical nicotinic agonist bound conformations conferring subtype selectivity. Proc Natl Acad Sci USA (2008) 105(5):1728–32. 10.1073/pnas.0711724105 PMC2234212 18230720

[128] Beker F Weber M Fink RH Adams DJ. Muscarinic and nicotinic ACh receptor activation differentially mobilize Ca2+ in rat intracardiac ganglion neurons. J Neurophysiol (2003) 90(3):1956–64. 10.1152/jn.01079.2002 12761283

[129] Lu B Kwan K Levine YA Olofsson PS Yang H Li J. alpha7 nicotinic acetylcholine receptor signaling inhibits inflammasome activation by preventing mitochondrial DNA release. Mol Med (2014) 20:350–8. 10.2119/molmed.2013.00117 PMC4153835 24849809

[130] Nakane S Mukaino A Higuchi O Watari M Maeda Y Yamakawa M. Autoimmune autonomic ganglionopathy: an update on diagnosis and treatment. Expert Rev Neurother (2018) 18(12):953–65. 10.1080/14737175.2018.1540304 30352532

[131] Yamakawa M Mukaino A Kimura A Nagasako Y Kitazaki Y Maeda Y. Antibodies to the α3 subunit of the ganglionic-type nicotinic acetylcholine receptors in patients with autoimmune encephalitis. J Neuroimmunol (2020) 349:577399. 10.1016/j.jneuroim.2020.577399 32980672

[132] Paz ML Barrantes FJ. Autoimmune Attack of the Neuromuscular Junction in Myasthenia Gravis: Nicotinic Acetylcholine Receptors and Other Targets. ACS Chem Neurosci (2019) 10(5):2186–94. 10.1021/acschemneuro.9b00041 30916550

[133] Hagforsen E Edvinsson M Nordlind K Michaëlsson G. Expression of nicotinic receptors in the skin of patients with palmoplantar pustulosis. Br J Dermatol (2002) 146(3):383–91. 10.1046/j.1365-2133.2002.04640.x 11952537

[134] Abu Zeid O Abdel-Aziz A Rashed LA Said ER. Role of the cutaneous extraneuronal cholinergic system in the pathogenesis of psoriasis: a case-control study. Clin Exp Dermatol (2020) 45(4):432–7. 10.1111/ced.14124 31614011

[135] Westman M Engström M Catrina AI Lampa J. Cell specific synovial expression of nicotinic alpha 7 acetylcholine receptor in rheumatoid arthritis and psoriatic arthritis. Scand J Immunol (2009) 70(2):136–40. 10.1111/j.1365-3083.2009.02266.x 19630919

[136] Chen J Cheuk IWY Shin VY Kwong A. Acetylcholine receptors: Key players in cancer development. Surg Oncol (2019) 31:46–53. 10.1016/j.suronc.2019.09.003 31536927

[137] Shulepko MA Bychkov ML Shlepova OV Shenkarev ZO Kirpichnikov MP Lyukmanova EN. Human secreted protein SLURP-1 abolishes nicotine-induced proliferation, PTEN down-regulation and α7-nAChR expression up-regulation in lung cancer cells. Int Immunopharmacol (2020) 82:106303. 10.1016/j.intimp.2020.106303 32106059

[138] Tian G Lu JY Hu S Lü Y Wang HW Yang Y. Effect of carbachol on dendritic cell function in the lipopolysaccharides induced murine sepsis model. Zhongguo Wei Zhong Bing Ji Jiu Yi Xue (2006) 18(11):684–6. 17092423

[139] Zhou L Jiang ZM Qiu XM Zhang YK Zhang FX Wang YX. Carbachol alleviates myocardial injury in septic rats through PI3K/AKT signaling pathway. Eur Rev Med Pharmacol Sci (2020) 24(10):5650–8. 10.26355/eurrev_202005_21356 32495900

[140] Kanefsky J Lenburg M Hai CM. Cholinergic receptor and cyclic stretch-mediated inflammatory gene expression in intact ASM. Am J Respir Cell Mol Biol (2006) 34(4):417–25. 10.1165/rcmb.2005-0326OC PMC2644203 16339998

[141] Duncan G Collison DJ. Role of the non-neuronal cholinergic system in the eye: a review. Life Sci (2003) 72(18-19):2013–9. 10.1016/s0024-3205(03)00064-x 12628451

[142] Zhang S Petro TM. The effect of nicotine on murine CD4 T cell responses. Int J Immunopharmacol (1996) 18(8-9):467–78. 10.1016/s0192-0561(96)00054-9 9023586

[143] Takahashi HK Iwagaki H Hamano R Yoshino T Tanaka N Nishibori M. Effect of nicotine on IL-18-initiated immune response in human monocytes. J Leukoc Biol (2006) 80(6):1388–94. 10.1189/jlb.0406236 16966384

[144] Balfour DJ Fagerström KO. Pharmacology of nicotine and its therapeutic use in smoking cessation and neurodegenerative disorders. Pharmacol Ther (1996) 72(1):51–81. 10.1016/s0163-7258(96)00099-x 8981571

[145] Vieyra-Reyes P Venebra-Muñoz A Rivas-Santiago B García-García F. Nicotine as an antidepressant and regulator of sleep in subjects with depression. Rev Neurol (2009) 49(12):661–7. 10.33588/rn.4912.2009158 20013719

[146] Matthews JB Chen FM Milward MR Wright HJ Carter K McDonagh A. Effect of nicotine, cotinine and cigarette smoke extract on the neutrophil respiratory burst. J Clin Periodontol (2011) 38(3):208–18. 10.1111/j.1600-051X.2010.01676.x 21214612

[147] Balter M. Firing of toxicologist prompts protest. Science (1994) 264(5162):1076. 10.1126/science.8178163 8178163

[148] Donnelly-Roberts DL Xue IC Arneric SP Sullivan JP. In vitro neuroprotective properties of the novel cholinergic channel activator (ChCA), ABT-418. Brain Res (1996) 719(1-2):36–44. 10.1016/0006-8993(96)00063-7 8782861

[149] Wilens TE Biederman J Spencer TJ Bostic J Prince J Monuteaux MC. A pilot controlled clinical trial of ABT-418, a cholinergic agonist, in the treatment of adults with attention deficit hyperactivity disorder. Am J Psychiatry (1999) 156(12):1931–7. 10.1176/ajp.156.12.1931 10588407

[150] Potter A Corwin J Lang J Piasecki M Lenox R Newhouse PA. Acute effects of the selective cholinergic channel activator (nicotinic agonist) ABT-418 in Alzheimer’s disease. Psychopharmacol (Berl) (1999) 142(4):334–42. 10.1007/s002130050897 10229057

[151] Koval L Lykhmus O Zhmak M Khruschov A Tsetlin V Magrini E. Differential involvement of alpha4beta2, alpha7 and alpha9alpha10 nicotinic acetylcholine receptors in B lymphocyte activation in vitro. Int J Biochem Cell Biol (2011) 43(4):516–24. 10.1016/j.biocel.2010.12.003 21146628

[152] Mellon RD Bayer BM. The effects of morphine, nicotine and epibatidine on lymphocyte activity and hypothalamic-pituitary-adrenal axis responses. J Pharmacol Exp Ther (1999) 288(2):635–42. 9918569

[153] Marshall CG Ogden DC Colquhoun D. The actions of suxamethonium (succinyldicholine) as an agonist and channel blocker at the nicotinic receptor of frog muscle. J Physiol (1990) 428:155–74. 10.1113/jphysiol.1990.sp018205 PMC1181640 2133043

[154] Delogu G Antonucci A Moretti S Marandola M Tellan G Signore M. Oxidative stress and mitochondrial glutathione in human lymphocytes exposed to clinically relevant anesthetic drug concentrations. J Clin Anesth (2004) 16(3):189–94. 10.1016/j.jclinane.2003.07.007 15217658

[155] Sánchez Palacios A Ortiz Ponce M Rodríguez Pérez A Schamann Medina F García Marrero JA. Modification of mediators of immune reaction after general anaesthesia. Allergol Immunopathol (Madr) (2004) 32(6):352–60. 10.1016/s0301-0546(04)79268-x 15617663

[156] Shao Z Li Q Wang S Chen Z. Protective effects of PNU−282987 on sepsis−induced acute lung injury in mice. Mol Med Rep (2019) 19(5):3791–8. 10.3892/mmr.2019.10016 30864715

[157] Pinheiro NM Santana FP Almeida RR Guerreiro M Martins MA Caperuto LC. Acute lung injury is reduced by the α7nAChR agonist PNU-282987 through changes in the macrophage profile. FASEB J (2017) 31(1):320–32. 10.1096/fj.201600431R 27729414

[158] Zanetti SR Ziblat A Torres NI Zwirner NW Bouzat C. Expression and Functional Role of alpha7 Nicotinic Receptor in Human Cytokine-stimulated Natural Killer (NK) Cells. J Biol Chem (2016) 291(32):16541–52. 10.1074/jbc.M115.710574 PMC4974370 27284006

[159] Vicens P Ribes D Heredia L Torrente M Domingo JL. Motor and anxiety effects of PNU-282987, an alpha7 nicotinic receptor agonist, and stress in an animal model of Alzheimer’s disease. Curr Alzheimer Res (2013) 10(5):516–23. 10.2174/15672050113109990130 23566346

[160] Hou Z Zhou Y Yang H Liu Y Mao X Qin X. Alpha7 nicotinic acetylcholine receptor activation protects against myocardial reperfusion injury through modulation of autophagy. Biochem Biophys Res Commun (2018) 500(2):357–64. 10.1016/j.bbrc.2018.04.077 29665360

[161] Sudo RT Hayashida K Santos AN Kawatani M Monteiro CE Moreira RD. Novel agonist of alpha4beta2* neuronal nicotinic receptor with antinociceptive efficacy in rodent models of acute and chronic pain. J Pain Res (2018) 11:2453–62. 10.2147/JPR.S169637 PMC6214310 30464575

[162] Liu EYL Xia Y Kong X Guo MSS Yu AXD Zheng BZY. Interacting with. Acta Pharm Sin B (2020) 10(10):1926–42. 10.1016/j.apsb.2020.05.005 PMC7606108 33163344

[163] Xue R Wan Y Sun X Zhang X Gao W Wu W. Nicotinic Mitigation of Neuroinflammation and Oxidative Stress After Chronic Sleep Deprivation. Front Immunol (2019) 10:2546. 10.3389/fimmu.2019.02546 31736967 PMC6828928

[164] Krafft PR Altay O Rolland WB Duris K Lekic T Tang J. α7 nicotinic acetylcholine receptor agonism confers neuroprotection through GSK-3β inhibition in a mouse model of intracerebral hemorrhage. Stroke (2012) 43(3):844–50. 10.1161/STROKEAHA.111.639989 PMC3293395 22207510

[165] Sérrière S Doméné A Vercouillie J Mothes C Bodard S Rodrigues N. Assessment of the Protection of Dopaminergic Neurons by an α7 Nicotinic Receptor Agonist, PHA 543613 Using [(18)F]LBT-999 in a Parkinson’s Disease Rat Model. Front Med (Lausanne) (2015) 2:61. 10.3389/fmed.2015.00061 26389120 PMC4556971

[166] Bali ZK Inkeller J Csurgyók R Bruszt N Horváth H Hernádi I. Differential effects of α7 nicotinic receptor agonist PHA-543613 on spatial memory performance of rats in two distinct pharmacological dementia models. Behav Brain Res (2015) 278:404–10. 10.1016/j.bbr.2014.10.030 25447295

[167] Thomsen MS Mikkelsen JD. The α7 nicotinic acetylcholine receptor ligands methyllycaconitine, NS6740 and GTS-21 reduce lipopolysaccharide-induced TNF-α release from microglia. J Neuroimmunol (2012) 251(1-2):65–72. 10.1016/j.jneuroim.2012.07.006 22884467

[168] Bagdas D Wilkerson JL Kulkarni A Toma W AlSharari S Gul Z. The α7 nicotinic receptor dual allosteric agonist and positive allosteric modulator GAT107 reverses nociception in mouse models of inflammatory and neuropathic pain. Br J Pharmacol (2016) 173(16):2506–20. 10.1111/bph.13528 PMC4959951 27243753

[169] Gauthier AG Wu J Lin M Sitapara R Kulkarni A Thakur GA. The Positive Allosteric Modulation of alpha7-Nicotinic Cholinergic Receptors by GAT107 Increases Bacterial Lung Clearance in Hyperoxic Mice by Decreasing Oxidative Stress in Macrophages. Antioxidants (Basel) (2021) 10(1):135. 10.3390/antiox10010135 33477969 PMC7835977

[170] Grandi A Zini I Flammini L Cantoni AM Vivo V Ballabeni V. α7 Nicotinic Agonist AR-R17779 Protects Mice against 2,4,6-Trinitrobenzene Sulfonic Acid-Induced Colitis in a Spleen-Dependent Way. Front Pharmacol (2017) 8:809. 10.3389/fphar.2017.00809 29167641 PMC5682330

[171] The FO Boeckxstaens GE Snoek SA Cash JL Bennink R Larosa GJ. Activation of the cholinergic anti-inflammatory pathway ameliorates postoperative ileus in mice. Gastroenterology (2007) 133(4):1219–28. 10.1053/j.gastro.2007.07.022 17919496

[172] van Maanen MA Lebre MC van der Poll T LaRosa GJ Elbaum D Vervoordeldonk MJ. Stimulation of nicotinic acetylcholine receptors attenuates collagen-induced arthritis in mice. Arthritis Rheum (2009) 60(1):114–22. 10.1002/art.24177 19116908

[173] Lopes F Graepel R Reyes JL Wang A Petri B McDougall JJ. Involvement of Mast Cells in α7 Nicotinic Receptor Agonist Exacerbation of Freund’s Complete Adjuvant-Induced Monoarthritis in Mice. Arthritis Rheumatol (2016) 68(2):542–52. 10.1002/art.39411 26314943

[174] Galitovskiy V Kuruvilla SA Sevriokov E Corches A Pan ML Kalantari-Dehaghi M. Development of novel approach to diagnostic imaging of lung cancer with. J Cancer Res Ther (Manch) (2013) 1(4):128–37. 10.14312/2052-4994.2013-20 PMC5443253 28553544

[175] Fraser PJ. Pharmacological actions of pure muscarine chloride. Br J Pharmacol Chemother (1957) 12(1):47–52. 10.1111/j.1476-5381.1957.tb01361.x 13413151 PMC1509643

[176] Wilson ID Soltis RD Olson RE Erlandsen SL. Cholinergic stimulation of immunoglobulin A secretion in rat intestine. Gastroenterology (1982) 83(4):881–8. 10.1016/S0016-5085(82)80020-6 6125453

[177] Yu P Zhou W Liu L Tang YB Song Y Lu JJ. L-Satropane Prevents Retinal Neuron Damage by Attenuating Cell Apoptosis and Abeta Production via Activation of M1 Muscarinic Acetylcholine Receptor. Curr Eye Res (2017) 42(9):1319–26. 10.1080/02713683.2017.1315142 28632409

[178] Fujino H Kitamura Y Yada T Uehara T Nomura Y. Stimulatory roles of muscarinic acetylcholine receptors on T cell antigen receptor/CD3 complex-mediated interleukin-2 production in human peripheral blood lymphocytes. Mol Pharmacol (1997) 51(6):1007–14. 10.1124/mol.51.6.1007 9187267

[179] Nomura J Hosoi T Okuma Y Nomura Y. The presence and functions of muscarinic receptors in human T cells: the involvement in IL-2 and IL-2 receptor system. Life Sci (2003) 72(18-19):2121–6. 10.1016/s0024-3205(03)00071-7 12628467

[180] Razani-Boroujerdi S Behl M Hahn FF Pena-Philippides JC Hutt J Sopori ML. Role of muscarinic receptors in the regulation of immune and inflammatory responses. J Neuroimmunol (2008) 194(1-2):83–8. 10.1016/j.jneuroim.2007.11.019 PMC2323336 18190972

[181] Munyaka P Rabbi MF Pavlov VA Tracey KJ Khafipour E Ghia JE. Central muscarinic cholinergic activation alters interaction between splenic dendritic cell and CD4+CD25- T cells in experimental colitis. PloS One (2014) 9(10):e109272. 10.1371/journal.pone.0109272 25295619 PMC4190311

[182] Pavlov VA Ochani M Gallowitsch-Puerta M Ochani K Huston JM Czura CJ. Central muscarinic cholinergic regulation of the systemic inflammatory response during endotoxemia. Proc Natl Acad Sci USA (2006) 103(13):5219–23. 10.1073/pnas.0600506103 PMC1405626 16549778

[183] Weber J Keating GM. Cevimeline. Drugs (2008) 68(12):1691–8. 10.2165/00003495-200868120-00006 18681491

[184] Renz BW Tanaka T Sunagawa M Takahashi R Jiang Z Macchini M. Cholinergic Signaling via Muscarinic Receptors Directly and Indirectly Suppresses Pancreatic Tumorigenesis and Cancer Stemness. Cancer Discovery (2018) 8(11):1458–73. 10.1158/2159-8290.CD-18-0046 PMC6214763 30185628

[185] McLean LP Smith A Cheung L Sun R Grinchuk V Vanuytsel T. Type 3 Muscarinic Receptors Contribute to Clearance of Citrobacter rodentium. Inflammation Bowel Dis (2015) 21(8):1860–71. 10.1097/MIB.0000000000000408 PMC4821008 25985244

[186] Ginder PA Ousley M Hinthorn D Liu C Abdou NI. Hidradenitis suppurativa: evidence for a bactericidal defect correctable by cholinergic agonist in vitro and in vivo. J Clin Immunol (1982) 2(3):237–41. 10.1007/BF00915227 6126491

[187] Cosyns SM Shiva S Lefebvre RA. Protective effect of exogenous nitrite in postoperative ileus. Br J Pharmacol (2015) 172(20):4864–74. 10.1111/bph.13255 PMC4621985 26227770

[188] Greco FA Simms NJ Athappilly GK. Bethanechol as a Corrective for Urinary Retention Associated With Olanzapine Administration. Prim Care Companion CNS Disord (2019) 21(5):19l02429. 10.4088/PCC.19l02429 31682336

[189] Cristofaro I Limongi C Piscopo P Crestini A Guerriero C Fiore M. M2 Receptor Activation Counteracts the Glioblastoma Cancer Stem Cell Response to Hypoxia Condition. Int J Mol Sci (2020) 21(5):1700. 10.3390/ijms21051700 PMC7084794 32131421

[190] Park HY Park C Hwang HJ Kim BW Kim GY Kim CM. 7,8-Dihydroxyflavone attenuates the release of pro-inflammatory mediators and cytokines in lipopolysaccharide-stimulated BV2 microglial cells through the suppression of the NF-κB and MAPK signaling pathways. Int J Mol Med (2014) 33(4):1027–34. 10.3892/ijmm.2014.1652 24535427

[191] Park HY Kim GY Hyun JW Hwang HJ Kim ND Kim BW. 7,8-Dihydroxyflavone exhibits anti-inflammatory properties by downregulating the NF-κB and MAPK signaling pathways in lipopolysaccharide-treated RAW264.7 cells. Int J Mol Med (2012) 29(6):1146–52. 10.3892/ijmm.2012.935 22427249

[192] Chen C Wang Z Zhang Z Liu X Kang SS Zhang Y. The prodrug of 7,8-dihydroxyflavone development and therapeutic efficacy for treating Alzheimer’s disease. Proc Natl Acad Sci USA (2018) 115(3):578–83. 10.1073/pnas.1718683115 PMC5777001 29295929

[193] Yang YJ Li YK Wang W Wan JG Yu B Wang MZ. Small-molecule TrkB agonist 7,8-dihydroxyflavone reverses cognitive and synaptic plasticity deficits in a rat model of schizophrenia. Pharmacol Biochem Behav (2014) 122:30–6. 10.1016/j.pbb.2014.03.013 24662915

[194] Jayasuriya GM Elmslie G Burstein ES Ellis J. Dronedarone Modulates M1 and M3 Muscarinic Receptors with Subtype Selectivity, Functional Selectivity, and Probe Dependence. Pharmacology (2017) 99(3-4):128–38. 10.1159/000453362 27992867

[195] Stahl E Ellis J. Novel allosteric effects of amiodarone at the muscarinic M5 receptor. J Pharmacol Exp Ther (2010) 334(1):214–22. 10.1124/jpet.109.165316 PMC2912050 20348203

[196] Nakajima K Yamazaki K Yamada E Kanaji Y Kosaka S Sato K. Amiodarone stimulates interleukin-6 production in cultured human thyrocytes, exerting cytotoxic effects on thyroid follicles in suspension culture. Thyroid (2001) 11(2):101–9. 10.1089/105072501300042703 11288978

[197] Fogoros RN. Major clinical trials assessing the prophylactic use of amiodarone in patients with ventricular tachyarrhythmias. Control Clin Trials (1996) 17(3 Suppl):37S–46S. 10.1016/s0197-2456(96)00017-7 8877266

[198] Naccarelli GV Wolbrette DL Dell’Orfano JT Patel HM Luck JC. Amiodarone: what have we learned from clinical trials? Clin Cardiol (2000) 23(2):73–82. 10.1002/clc.4960230203 10676597 PMC6655150

[199] Rosas-Ballina M Valdés-Ferrer SI Dancho ME Ochani M Katz D Cheng KF. Xanomeline suppresses excessive pro-inflammatory cytokine responses through neural signal-mediated pathways and improves survival in lethal inflammation. Brain Behav Immun (2015) 44:19–27. 10.1016/j.bbi.2014.07.010 25063706 PMC4624331

[200] Shekhar A Potter WZ Lightfoot J Lienemann J Dubé S Mallinckrodt C. Selective muscarinic receptor agonist xanomeline as a novel treatment approach for schizophrenia. Am J Psychiatry (2008) 165(8):1033–9. 10.1176/appi.ajp.2008.06091591 18593778

[201] Mirza NR Peters D Sparks RG. Xanomeline and the antipsychotic potential of muscarinic receptor subtype selective agonists. CNS Drug Rev (2003) 9(2):159–86. 10.1111/j.1527-3458.2003.tb00247.x PMC6741650 12847557

[202] Stelmach JE Liu L Patel SB Pivnichny JV Scapin G Singh S. Design and synthesis of potent, orally bioavailable dihydroquinazolinone inhibitors of p38 MAP kinase. Bioorg Med Chem Lett (2003) 13(2):277–80. 10.1016/s0960-894x(02)00752-7 12482439

[203] Chen ML Tsai TC Wang LK Lin YY Tsai YM Lee MC. Clozapine inhibits Th1 cell differentiation and causes the suppression of IFN-γ production in peripheral blood mononuclear cells. Immunopharmacol Immunotoxicol (2012) 34(4):686–94. 10.3109/08923973.2011.651535 22268679

[204] Chen ML Wu S Tsai TC Wang LK Tsai FM. Regulation of macrophage immune responses by antipsychotic drugs. Immunopharmacol Immunotoxicol (2013) 35(5):573–80. 10.3109/08923973.2013.828744 23981042

[205] Chen ML Wu S Tsai TC Wang LK Tsai FM. Regulation of neutrophil phagocytosis of Escherichia coli by antipsychotic drugs. Int Immunopharmacol (2014) 23(2):550–7. 10.1016/j.intimp.2014.09.030 25448498

[206] Leucht S Cipriani A Spineli L Mavridis D Orey D Richter F. Comparative efficacy and tolerability of 15 antipsychotic drugs in schizophrenia: a multiple-treatments meta-analysis. Lancet (2013) 382(9896):951–62. 10.1016/S0140-6736(13)60733-3 23810019

[207] Kanashiro A Talbot J Peres RS Pinto LG Bassi GS Cunha TM. Neutrophil Recruitment and Articular Hyperalgesia in Antigen-Induced Arthritis are Modulated by the Cholinergic Anti-Inflammatory Pathway. Basic Clin Pharmacol Toxicol (2016) 119(5):453–7. 10.1111/bcpt.12611 27098245

[208] Pothoulakis C Castagliuolo I Leeman SE. Neuroimmune mechanisms of intestinal responses to stress. Role of corticotropin-releasing factor and neurotensin. Ann N Y Acad Sci (1998) 840:635–48. 10.1111/j.1749-6632.1998.tb09602.x 9629290

[209] Young HS Herbette LG Skita V. Alpha-bungarotoxin binding to acetylcholine receptor membranes studied by low angle X-ray diffraction. Biophys J (2003) 85(2):943–53. 10.1016/S0006-3495(03)74533-0 PMC1303215 12885641

[210] De Rosa MJ Dionisio L Agriello E Bouzat C. Esandi MeC. Alpha 7 nicotinic acetylcholine receptor modulates lymphocyte activation. Life Sci (2009) 85(11-12):444–9. 10.1016/j.lfs.2009.07.010 19632243

[211] McMahon LR. Green tobacco sickness: mecamylamine, varenicline, and nicotine vaccine as clinical research tools and potential therapeutics. Expert Rev Clin Pharmacol (2019) 12(3):189–95. 10.1080/17512433.2019.1570844 PMC6786486 30650314

[212] Damaj MI Flood P Ho KK May EL Martin BR. Effect of dextrometorphan and dextrorphan on nicotine and neuronal nicotinic receptors: in vitro and in vivo selectivity. J Pharmacol Exp Ther (2005) 312(2):780–5. 10.1124/jpet.104.075093 15356218

[213] Stahl SM. Dextromethorphan/Bupropion: A Novel Oral NMDA (N-methyl-d-aspartate) Receptor Antagonist with Multimodal Activity. CNS Spectr (2019) 24(5):461–6. 10.1017/S1092852919001470 31566163

[214] Chen DY Song PS Hong JS Chu CL Pan IH Chen YM. Dextromethorphan inhibits activations and functions in dendritic cells. Clin Dev Immunol (2013) 2013:125643. 10.1155/2013/125643 23781253 PMC3679715

[215] Werling LL Lauterbach EC Calef U. Dextromethorphan as a potential neuroprotective agent with unique mechanisms of action. Neurologist (2007) 13(5):272–93. 10.1097/NRL.0b013e3180f60bd8 17848867

[216] Zhang W Wang T Qin L Gao HM Wilson B Ali SF. Neuroprotective effect of dextromethorphan in the MPTP Parkinson’s disease model: role of NADPH oxidase. FASEB J (2004) 18(3):589–91. 10.1096/fj.03-0983fje 14734632

[217] Chechneva OV Mayrhofer F Daugherty DJ Pleasure DE Hong JS Deng W. Low dose dextromethorphan attenuates moderate experimental autoimmune encephalomyelitis by inhibiting NOX2 and reducing peripheral immune cells infiltration in the spinal cord. Neurobiol Dis (2011) 44(1):63–72. 10.1016/j.nbd.2011.06.004 21704706 PMC3153572

[218] Chen DY Lin CC Chen YM Chao YH Yang DH. Dextromethorphan Exhibits Anti-inflammatory and Immunomodulatory Effects in a Murine Model of Collagen-Induced Arthritis and in Human Rheumatoid Arthritis. Sci Rep (2017) 7(1):11353. 10.1038/s41598-017-11378-8 28900117 PMC5595833

[219] Cummings JL Lyketsos CG Peskind ER Porsteinsson AP Mintzer JE Scharre DW. Effect of Dextromethorphan-Quinidine on Agitation in Patients With Alzheimer Disease Dementia: A Randomized Clinical Trial. JAMA (2015) 314(12):1242–54. 10.1001/jama.2015.10214 26393847

[220] Gredal O Werdelin L Bak S Christensen PB Boysen G Kristensen MO. A clinical trial of dextromethorphan in amyotrophic lateral sclerosis. Acta Neurol Scand (1997) 96(1):8–13. 10.1111/j.1600-0404.1997.tb00231.x 9262126

[221] De-Pu Z Li-Sha G Guang-Yi C Xiaohong G Chao X Cheng Z. The cholinergic anti-inflammatory pathway ameliorates acute viral myocarditis in mice by regulating CD4. Virulence (2018) 9(1):1364–76. 10.1080/21505594.2018.1482179 PMC6141146 30176160

[222] Kamens HM Peck C Garrity C Gechlik A Jenkins BC Rajan A. alpha6beta2 nicotinic acetylcholine receptors influence locomotor activity and ethanol consumption. Alcohol (2017) 61:43–9. 10.1016/j.alcohol.2017.02.178 PMC5466835 28457669

[223] Beckmann JS Meyer AC Pivavarchyk M Horton DB Zheng G Smith AM. r-bPiDI, an α6β2* Nicotinic Receptor Antagonist, Decreases Nicotine-Evoked Dopamine Release and Nicotine Reinforcement. Neurochem Res (2015) 40(10):2121–30. 10.1007/s11064-015-1680-4 PMC4639919 26227997

[224] Cuny H Yu R Tae HS Kompella SN Adams DJ. alpha-Conotoxins active at alpha3-containing nicotinic acetylcholine receptors and their molecular determinants for selective inhibition. Br J Pharmacol (2018) 175(11):1855–68. 10.1111/bph.13852 PMC5979624 28477355

[225] Zazueta-Favela D Donis-Maturano L Licea-Navarro AF Bernáldez-Sarabia J Dan KWL Cota-Arce JM. Marine peptides as immunomodulators. Immunopharmacol Immunotoxicol (2019) 41(4):463–8. 10.1080/08923973.2019.1641114 31339393

[226] Padilla A Keating P Hartmann JX Marí F. Effects of α-conotoxin ImI on TNF-α, IL-8 and TGF-β expression by human macrophage-like cells derived from THP-1 pre-monocytic leukemic cells. Sci Rep (2017) 7(1):12742. 10.1038/s41598-017-11586-2 28986583 PMC5630575

[227] Gundisch D Eibl C. Nicotinic acetylcholine receptor ligands, a patent review (2006-2011). Expert Opin Ther Pat (2011) 21(12):1867–96. 10.1517/13543776.2011.637919 PMC3495178 22098319

[228] Zaveri N Jiang F Olsen C Polgar W Toll L. Novel α3β4 nicotinic acetylcholine receptor-selective ligands. Discovery, structure-activity studies, and pharmacological evaluation. J Med Chem (2010) 53(22):8187–91. 10.1021/jm1006148 PMC2997436 20979364

[229] Maisonneuve IM Glick SD. Anti-addictive actions of an iboga alkaloid congener: a novel mechanism for a novel treatment. Pharmacol Biochem Behav (2003) 75(3):607–18. 10.1016/s0091-3057(03)00119-9 12895678

[230] Toll L Zaveri NT Polgar WE Jiang F Khroyan TV Zhou W. AT-1001: a high affinity and selective alpha3beta4 nicotinic acetylcholine receptor antagonist blocks nicotine self-administration in rats. Neuropsychopharmacology (2012) 37(6):1367–76. 10.1038/npp.2011.322 PMC3327842 22278092

[231] Leffler DA Kelly CP Abdallah HZ Colatrella AM Harris LA Leon F. A randomized, double-blind study of larazotide acetate to prevent the activation of celiac disease during gluten challenge. Am J Gastroenterol (2012) 107(10):1554–62. 10.1038/ajg.2012.211 PMC3463856 22825365

[232] Brown KC Lau JK Dom AM Witte TR Luo H Crabtree CM. MG624, an alpha7-nAChR antagonist, inhibits angiogenesis via the Egr-1/FGF2 pathway. Angiogenesis (2012) 15(1):99–114. 10.1007/s10456-011-9246-9 22198237

[233] Fuentes JM Fulton WB Nino D Talamini MA Maio AD. Atropine treatment modifies LPS-induced inflammatory response and increases survival. Inflammation Res (2008) 57(3):111–7. 10.1007/s00011-007-7134-y 18369575

[234] McDermott SW Altekruse JM. Dynamic model for preventing mental retardation in the population: the importance of poverty and deprivation. Res Dev Disabil (1994) 15(1):49–65. 10.1016/0891-4222(94)90038-8 8190972

[235] Qiu YH Peng YP Zhang QQ Wang JH. [Effect of acetylcholine on the proliferation of T lymphocyte of rat spleen]. Sheng Li Xue Bao (1995) 47(3):275–80. 7570114

[236] Wu XJ Liu HM Song XM Zhao B Leng Y Wang EY. Penehyclidine hydrochloride inhibits TLR4 signaling and inflammation, and attenuates blunt chest trauma and hemorrhagic shock-induced acute lung injury in rats. Mol Med Rep (2018) 17(5):6327–36. 10.3892/mmr.2018.8644 PMC5928610 29488614

[237] Bhattacharjee A Chaudhuri R Dash JJ Saha M Choudhury L Roy S. Pre-treatment with Scopolamine Naturally Suppresses Japanese Encephalitis Viral Load in Embryonated Chick Through Regulation of Multiple Signaling Pathways. Appl Biochem Biotechnol (2021). 10.1007/s12010-021-03526-8 33620666

[238] Pitcher JD De Paiva CS Pelegrino FS McClellan AJ Raince JK Pangelinan SB. Pharmacological cholinergic blockade stimulates inflammatory cytokine production and lymphocytic infiltration in the mouse lacrimal gland. Invest Ophthalmol Vis Sci (2011) 52(6):3221–7. 10.1167/iovs.09-4212 PMC3109024 21273534

[239] Spinks A Wasiak J. Scopolamine (hyoscine) for preventing and treating motion sickness. Cochrane Database Syst Rev (2011) 6:CD002851. 10.1002/14651858.CD002851.pub4 PMC7138049 21678338

[240] Ripamonti C Mercadante S Groff L Zecca E De Conno F Casuccio A. Role of octreotide, scopolamine butylbromide, and hydration in symptom control of patients with inoperable bowel obstruction and nasogastric tubes: a prospective randomized trial. J Pain Symptom Manage (2000) 19(1):23–34. 10.1016/s0885-3924(99)00147-5 10687323

[241] Smith PM Troughton AH Gleeson F Walters J McCarthy CF. Pirenzepine in non-ulcer dyspepsia: a double-blind multicentre trial. J Int Med Res (1990) 18(1):16–20. 10.1177/030006059001800103 2185962

[242] Morelli A Pelli A Narducci F Spadacini A. Pirenzepine in the treatment of gastric ulcer. A double-blind short-term clinical trial. Scand J Gastroenterol Suppl (1979) 57:51–5. 396658

[243] Miao Y Bi XY Zhao M Jiang HK Liu JJ Li DL. Acetylcholine inhibits tumor necrosis factor α activated endoplasmic reticulum apoptotic pathway via EGFR-PI3K signaling in cardiomyocytes. J Cell Physiol (2015) 230(4):767–74. 10.1002/jcp.24800 25201632

[244] Croom KF Keating GM. Darifenacin: in the treatment of overactive bladder. Drugs Aging (2004) 21(13):885–92. 10.2165/00002512-200421130-00005 discussion 93-4. 15493952

[245] Mashimo M Fujii M Sakagawa N Fukuda Y Imanaka R Fujii T. Muscarinic Acetylcholine Receptors Modulate Interleukin-6 Production and Immunoglobulin Class Switching in Daudi Cells. Biol Pharm Bull (2020) 43(12):1950–3. 10.1248/bpb.b20-00461 33268714

[246] Xu ZP Song Y Yang K Zhou W Hou LN Zhu L. M3 mAChR-mediated IL-8 expression through PKC/NF-κB signaling pathways. Inflammation Res (2014) 63(6):463–73. 10.1007/s00011-014-0718-4 24522860

[247] Yazdani N Sadeghi R Momeni-Moghaddam H Zarifmahmoudi L Ehsaei A. Comparison of cyclopentolate versus tropicamide cycloplegia: A systematic review and meta-analysis. J Optom (2018) 11(3):135–43. 10.1016/j.optom.2017.09.001 PMC6039578 29132914

[248] Caulfield MP Birdsall NJ. International Union of Pharmacology. XVII. Classification of muscarinic acetylcholine receptors. Pharmacol Rev (1998) 50(2):279–90. 9647869

[249] Hulme EC Birdsall NJ Buckley NJ. Muscarinic receptor subtypes. Annu Rev Pharmacol Toxicol (1990) 30:633–73. 10.1146/annurev.pa.30.040190.003221 2188581

[250] Maeda S Qu Q Robertson MJ Skiniotis G Kobilka BK. Structures of the M1 and M2 muscarinic acetylcholine receptor/G-protein complexes. Science (6440) 2019) 364:552–7. 10.1126/science.aaw5188 PMC7034192 31073061

[251] Lehner KR Silverman HA Addorisio ME Roy A Al-Onaizi MA Levine Y. Forebrain Cholinergic Signaling Regulates Innate Immune Responses and Inflammation. Front Immunol (2019) 10:585. 10.3389/fimmu.2019.00585 31024522 PMC6455130

[252] Lee BH Gauna AE Perez G Park YJ Pauley KM Kawai T. Autoantibodies against muscarinic type 3 receptor in Sjögren’s syndrome inhibit aquaporin 5 trafficking. PloS One (2013) 8(1):e53113. 10.1371/journal.pone.0053113 23382834 PMC3559734

[253] Scherbaum I Heidecke H Bunte K Peters U Beikler T Boege F. Autoantibodies against M. Aging (Albany NY) (2020) 12(16):16609–20. 10.18632/aging.103864 PMC7485715 32857064

[254] Loebel M Grabowski P Heidecke H Bauer S Hanitsch LG Wittke K. Antibodies to β adrenergic and muscarinic cholinergic receptors in patients with Chronic Fatigue Syndrome. Brain Behav Immun (2016) 52:32–9. 10.1016/j.bbi.2015.09.013 26399744

[255] Liu T Xie C Chen X Zhao F Liu AM Cho DB. Role of muscarinic receptor activation in regulating immune cell activity in nasal mucosa. Allergy (2010) 65(8):969–77. 10.1111/j.1398-9995.2009.02281.x 19951374

[256] Sethi A Kulkarni N Sonar S Lal G. Role of miRNAs in CD4 T cell plasticity during inflammation and tolerance. Front Genet (2013) 4:8. 10.3389/fgene.2013.00008 23386861 PMC3560369

[257] Kulkarni N Sonar SA Lal G. Plasticity of Th17 and Tregs and its clinical importance as therapeutic target in inflammatory bowel disease. Indian J Inflammation Res (2018) 1(1):R2. 10.15305/ijir/v1i1/258

[258] Willemze RA Brinkman DJ Welting O van Hamersveld PHP Verseijden C Luyer MD. Acetylcholine-producing T cells augment innate immune-driven colitis but are redundant in T cell-driven colitis. Am J Physiol Gastrointest Liver Physiol (2019) 317(5):G557–G68. 10.1152/ajpgi.00067.2019 31322912

[259] Rosas-Ballina M Olofsson PS Ochani M Valdes-Ferrer SI Levine YA Reardon C. Acetylcholine-synthesizing T cells relay neural signals in a vagus nerve circuit. Science (2011) 334(6052):98–101. 10.1126/science.1209985 21921156 PMC4548937

[260] Fujii T Horiguchi K Sunaga H Moriwaki Y Misawa H Kasahara T. SLURP-1, an endogenous alpha7 nicotinic acetylcholine receptor allosteric ligand, is expressed in CD205(+) dendritic cells in human tonsils and potentiates lymphocytic cholinergic activity. J Neuroimmunol (2014) 267(1-2):43–9. 10.1016/j.jneuroim.2013.12.003 24365495

[261] Nouri-Shirazi M Guinet E. Evidence for the immunosuppressive role of nicotine on human dendritic cell functions. Immunology (2003) 109(3):365–73. 10.1046/j.1365-2567.2003.01655.x PMC1782971 12807482

[262] Hernandez CP Morrow K Velasco C Wyczechowska DD Naura AS Rodriguez PC. Effects of cigarette smoke extract on primary activated T cells. Cell Immunol (2013) 282(1):38–43. 10.1016/j.cellimm.2013.04.005 23665673 PMC3676722

[263] Geng Y Savage SM Razani-Boroujerdi S Sopori ML. Effects of nicotine on the immune response. II. Chronic nicotine treatment induces T cell anergy. J Immunol (1996) 156(7):2384–90. 8786295

[264] Fujii YX Fujigaya H Moriwaki Y Misawa H Kasahara T Grando SA. Enhanced serum antigen-specific IgG1 and proinflammatory cytokine production in nicotinic acetylcholine receptor alpha7 subunit gene knockout mice. J Neuroimmunol (2007) 189(1-2):69–74. 10.1016/j.jneuroim.2007.07.003 17675251

[265] Oloris SC Frazer-Abel AA Jubala CM Fosmire SP Helm KM Robinson SR. Nicotine-mediated signals modulate cell death and survival of T lymphocytes. Toxicol Appl Pharmacol (2010) 242(3):299–309. 10.1016/j.taap.2009.10.020 19896492 PMC2813922

[266] Mashimo M Komori M Matsui YY Murase MX Fujii T Takeshima S. Distinct Roles of alpha7 nAChRs in Antigen-Presenting Cells and CD4(+) T Cells in the Regulation of T Cell Differentiation. Front Immunol (2019) 10:1102. 10.3389/fimmu.2019.01102 31214160 PMC6554293

[267] Zdanowski R Krzyzowska M Ujazdowska D Lewicka A Lewicki S. Role of alpha7 nicotinic receptor in the immune system and intracellular signaling pathways. Cent Eur J Immunol (2015) 40(3):373–9. 10.5114/ceji.2015.54602 PMC4655390 26648784

[268] De Rosa MJ Dionisio L Agriello E Bouzat C Esandi Mdel C. Alpha 7 nicotinic acetylcholine receptor modulates lymphocyte activation. Life Sci (2009) 85(11-12):444–9. 10.1016/j.lfs.2009.07.010 19632243

[269] Nizri E Irony-Tur-Sinai M Lory O Orr-Urtreger A Lavi E Brenner T. Activation of the cholinergic anti-inflammatory system by nicotine attenuates neuroinflammation via suppression of Th1 and Th17 responses. J Immunol (2009) 183(10):6681–8. 10.4049/jimmunol.0902212 19846875

[270] Liu Z Han B Li P Wang Z Fan Q. Activation of alpha7nAChR by nicotine reduced the Th17 response in CD4(+)T lymphocytes. Immunol Invest (2014) 43(7):667–74. 10.3109/08820139.2014.914532 24949556

[271] Wang DW Zhou RB Yao YM Zhu XM Yin YM Zhao GJ. Stimulation of α7 nicotinic acetylcholine receptor by nicotine increases suppressive capacity of naturally occurring CD4+CD25+ regulatory T cells in mice in vitro. J Pharmacol Exp Ther (2010) 335(3):553–61. 10.1124/jpet.110.169961 20843956

[272] Fujii T Watanabe Y Inoue T Kawashima K. Upregulation of mRNA encoding the M5 muscarinic acetylcholine receptor in human T- and B-lymphocytes during immunological responses. Neurochem Res (2003) 28(3-4):423–9. 10.1023/a:1022840416292 12675126

[273] Fujino H Uehara T Murayama T Okuma Y Ariga H Nomura Y. Extracellular signal regulated protein kinase and c-jun N-terminal kinase are involved in ml muscarinic receptor-enhanced interleukin-2 production pathway in Jurkat cells. Biol Pharm Bull (2000) 23(10):1198–205. 10.1248/bpb.23.1198 11041251

[274] Asthana S Greig NH Holloway HW Raffaele KC Berardi A Schapiro MB. Clinical pharmacokinetics of arecoline in subjects with Alzheimer’s disease. Clin Pharmacol Ther (1996) 60(3):276–82. 10.1016/S0009-9236(96)90054-5 8841150

[275] Wen XM Zhang YL Liu XM Guo SX Wang H. Immune responses in mice to arecoline mediated by lymphocyte muscarinic acetylcholine receptor. Cell Biol Int (2006) 30(12):1048–53. 10.1016/j.cellbi.2006.09.015 17084646

[276] Darby M Schnoeller C Vira A Culley FJ Bobat S Logan E. The M3 muscarinic receptor is required for optimal adaptive immunity to helminth and bacterial infection. PloS Pathog (2015) 11(1):e1004636. 10.1371/journal.ppat.1004636 25629518 PMC4309615

[277] Skok MV Grailhe R Agenes F Changeux JP. The role of nicotinic receptors in B-lymphocyte development and activation. Life Sci (2007) 80(24-25):2334–6. 10.1016/j.lfs.2007.02.005 17363009

[278] Skok M Grailhe R Agenes F Changeux JP. The role of nicotinic acetylcholine receptors in lymphocyte development. J Neuroimmunol (2006) 171(1-2):86–98. 10.1016/j.jneuroim.2005.09.011 16253349

[279] Skok M Grailhe R Changeux JP. Nicotinic receptors regulate B lymphocyte activation and immune response. Eur J Pharmacol (2005) 517(3):246–51. 10.1016/j.ejphar.2005.05.011 15963492

[280] Koval L Kalashnyk O Lykhmus O Skok M. alpha7 nicotinic acetylcholine receptors are involved in suppression of the antibody immune response. J Neuroimmunol (2018) 318:8–14. 10.1016/j.jneuroim.2018.01.012 29395323

[281] Koval LM Yu Lykhmus O Omelchenko DM Komisarenko SV Skok MV. The role of alpha7 nicotinic acetylcholine receptors in B lymphocyte activation. Ukr Biokhim Zh (1999) (2009) 81(4):5–11. 20387628

[282] Fujii YX Tashiro A Arimoto K Fujigaya H Moriwaki Y Misawa H. Diminished antigen-specific IgG1 and interleukin-6 production and acetylcholinesterase expression in combined M1 and M5 muscarinic acetylcholine receptor knockout mice. J Neuroimmunol (2007) 188(1-2):80–5. 10.1016/j.jneuroim.2007.05.017 17586055

[283] Hainke S Wildmann J Del Rey A. Deletion of muscarinic type 1 acetylcholine receptors alters splenic lymphocyte functions and splenic noradrenaline concentration. Int Immunopharmacol (2015) 29(1):135–42. 10.1016/j.intimp.2015.05.010 26002586

[284] Fujii T Kawashima K. Calcium signaling and c-Fos gene expression via M3 muscarinic acetylcholine receptors in human T- and B-cells. Jpn J Pharmacol (2000) 84(2):124–32. 10.1254/jjp.84.124 11128034

[285] Nagaraju K Cox A Casciola-Rosen L Rosen A. Novel fragments of the Sjogren’s syndrome autoantigens alpha-fodrin and type 3 muscarinic acetylcholine receptor generated during cytotoxic lymphocyte granule-induced cell death. Arthritis Rheum (2001) 44(10):2376–86. 10.1002/1529-0131(200110)44:10<2376::AID-ART402>3.0.CO;2-E 11665980

[286] Hogg N. Nicotine has suppressive effects on dendritic cell function. Immunology (2003) 109(3):329–30. 10.1046/j.1365-2567.2003.01685.x PMC1782992 12807476

[287] Liu D Li T Luo H Zuo X Liu S Wu S. The effect of the cholinergic anti-inflammatory pathway on collagen-induced arthritis involves the modulation of dendritic cell differentiation. Arthritis Res Ther (2018) 20(1):263. 10.1186/s13075-018-1759-9 30486874 PMC6262974

[288] Mashimo M Takeshima S Okuyama H Matsurida A Murase M Ono S. α7 nAChRs expressed on antigen presenting cells are insensitive to the conventional antagonists α-bungarotoxin and methyllycaconitine. Int Immunopharmacol (2020) 81:106276. 10.1016/j.intimp.2020.106276 32044666

[289] Gori S Vermeulen M Remes-Lenicov F Jancic C Scordo W Ceballos A. Acetylcholine polarizes dendritic cells toward a Th2-promoting profile. Allergy (2017) 72(2):221–31. 10.1111/all.12926 27138374

[290] Nouri-Shirazi M Kahlden C Nishino P Guinet E. Nicotine exposure alters the mRNA expression of Notch ligands in dendritic cells and their response to Th1-/Th2-promoting stimuli. Scand J Immunol (2015) 81(2):110–20. 10.1111/sji.12254 25418282

[291] Gao FG Wan da F Gu JR. Ex vivo nicotine stimulation augments the efficacy of therapeutic bone marrow-derived dendritic cell vaccination. Clin Cancer Res (2007) 13(12):3706–12. 10.1158/1078-0432.CCR-07-0028 17575236

[292] Mikulski Z Hartmann P Jositsch G Zaslona Z Lips KS Pfeil U. Nicotinic receptors on rat alveolar macrophages dampen ATP-induced increase in cytosolic calcium concentration. Respir Res (2010) 11:133. 10.1186/1465-9921-11-133 20920278 PMC2955664

[293] Wang H Yu M Ochani M Amella CA Tanovic M Susarla S. Nicotinic acetylcholine receptor alpha7 subunit is an essential regulator of inflammation. Nature (2003) 421(6921):384–8. 10.1038/nature01339 12508119

[294] Tarnawski L Reardon C Caravaca AS Rosas-Ballina M Tusche MW Drake AR. Adenylyl Cyclase 6 Mediates Inhibition of TNF in the Inflammatory Reflex. Front Immunol (2018) 9:2648. 10.3389/fimmu.2018.02648 30538698 PMC6277584

[295] Borovikova LV Ivanova S Zhang M Yang H Botchkina GI Watkins LR. Vagus nerve stimulation attenuates the systemic inflammatory response to endotoxin. Nature (6785) 2000) 405:458–62. 10.1038/35013070 10839541

[296] de Jonge WJ van der Zanden EP The FO Bijlsma MF van Westerloo DJ Bennink RJ. Stimulation of the vagus nerve attenuates macrophage activation by activating the Jak2-STAT3 signaling pathway. Nat Immunol (2005) 6(8):844–51. 10.1038/ni1229 16025117

[297] Yang YH Li DL Bi XY Sun L Yu XJ Fang HL. Acetylcholine Inhibits LPS-Induced MMP-9 Production and Cell Migration via the alpha7 nAChR-JAK2/STAT3 Pathway in RAW264.7 Cells. Cell Physiol Biochem (2015) 36(5):2025–38. 10.1159/000430170 26202362

[298] Maldifassi MC Atienza G Arnalich F Lopez-Collazo E Cedillo JL Martin-Sanchez C. A new IRAK-M-mediated mechanism implicated in the anti-inflammatory effect of nicotine via alpha7 nicotinic receptors in human macrophages. PloS One (2014) 9(9):e108397. 10.1371/journal.pone.0108397 25259522 PMC4178160

[299] Nemethova A Michel K Gomez-Pinilla PJ Boeckxstaens GE Schemann M. Nicotine attenuates activation of tissue resident macrophages in the mouse stomach through the beta2 nicotinic acetylcholine receptor. PloS One (2013) 8(11):e79264. 10.1371/journal.pone.0079264 24223920 PMC3815157

[300] Cailotto C Gomez-Pinilla PJ Costes LM van der Vliet J Di Giovangiulio M Nemethova A. Neuro-anatomical evidence indicating indirect modulation of macrophages by vagal efferents in the intestine but not in the spleen. PloS One (2014) 9(1):e87785. 10.1371/journal.pone.0087785 24489965 PMC3906221

[301] Moussa AT Rabung A Reichrath S Wagenpfeil S Dinh T Krasteva-Christ G. Modulation of macrophage phagocytosis in vitro-A role for cholinergic stimulation? Ann Anat (2017) 214:31–5. 10.1016/j.aanat.2017.07.007 28823709

[302] de la Torre E Genaro AM Ribeiro ML Pagotto R Pignataro OP Sales ME. Proliferative actions of muscarinic receptors expressed in macrophages derived from normal and tumor bearing mice. Biochim Biophys Acta (2008) 1782(2):82–9. 10.1016/j.bbadis.2007.11.005 18078830

[303] Mishra NC Rir-sima-ah J Boyd RT Singh SP Gundavarapu S Langley RJ. Nicotine inhibits Fc epsilon RI-induced cysteinyl leukotrienes and cytokine production without affecting mast cell degranulation through alpha 7/alpha 9/alpha 10-nicotinic receptors. J Immunol (2010) 185(1):588–96. 10.4049/jimmunol.0902227 PMC2954495 20505147

[304] Fantozzi R Masini E Blandina P Mannaioni PF Bani-Sacchi T. Release of histamine from rat mast cells by acetylcholine. Nature (1978) 273(5662):473–4. 10.1038/273473a0 78450

[305] Kaliner M Orange RP Austen KF. Immunological release of histamine and slow reacting substance of anaphylaxis from human lung. J Exp Med (1972) 136(3):556–67. 10.1084/jem.136.3.556 PMC2139264 4115132

[306] Sudheer PS Hall JE Donev R Read G Rowbottom A Williams PE. Nicotinic acetylcholine receptors on basophils and mast cells. Anaesthesia (2006) 61(12):1170–4. 10.1111/j.1365-2044.2006.04870.x 17090238

[307] Wallon C Persborn M Jonsson M Wang A Phan V Lampinen M. Eosinophils express muscarinic receptors and corticotropin-releasing factor to disrupt the mucosal barrier in ulcerative colitis. Gastroenterology (2011) 140(5):1597–607. 10.1053/j.gastro.2011.01.042 21277851

[308] Reinheimer T Baumgartner D Hohle KD Racke K Wessler I. Acetylcholine via muscarinic receptors inhibits histamine release from human isolated bronchi. Am J Respir Crit Care Med (1997) 156(2 Pt 1):389–95. 10.1164/ajrccm.156.2.96-12079 9279214

[309] Chahdi A Daeffler L Bueb JL Gies JP Landry Y. The M2 muscarinic receptor antagonist methoctramine activates mast cells via pertussis toxin-sensitive G proteins. Naunyn Schmiedebergs Arch Pharmacol (1998) 357(4):357–62. 10.1007/PL00005179 9606019

[310] Windmiller DA Backer JM. Distinct phosphoinositide 3-kinases mediate mast cell degranulation in response to G-protein-coupled versus FcepsilonRI receptors. J Biol Chem (2003) 278(14):11874–8. 10.1074/jbc.M211787200 12529321

[311] Nishida K Yamasaki S Ito Y Kabu K Hattori K Tezuka T. Fc{epsilon}RI-mediated mast cell degranulation requires calcium-independent microtubule-dependent translocation of granules to the plasma membrane. J Cell Biol (2005) 170(1):115–26. 10.1083/jcb.200501111 PMC2171390 15998803

[312] Djouder N Aneiros E Cavalie A Aktories K. Effects of large clostridial cytotoxins on activation of RBL 2H3-hm1 mast cells indicate common and different roles of Rac in FcepsilonRI and M1-receptor signaling. J Pharmacol Exp Ther (2003) 304(3):1243–50. 10.1124/jpet.102.045351 12604702

[313] Safronova VG Vulfius CA Shelukhina IV Mal’tseva VN Berezhnov AV Fedotova EI. Nicotinic receptor involvement in regulation of functions of mouse neutrophils from inflammatory site. Immunobiology (2016) 221(7):761–72. 10.1016/j.imbio.2016.01.016 26965141

[314] Huston JM Rosas-Ballina M Xue X Dowling O Ochani K Ochani M. Cholinergic neural signals to the spleen down-regulate leukocyte trafficking via CD11b. J Immunol (2009) 183(1):552–9. 10.4049/jimmunol.0802684 PMC2806576 19542466

[315] Dutta G Ghosh T. Effects of stimulation of muscarinic acetylcholine receptors in medial septum on some immune responses in rats. Neurosci Lett (2016) 619:155–61. 10.1016/j.neulet.2016.03.023 26987722

[316] Profita M Bonanno A Siena L Ferraro M Montalbano AM Pompeo F. Acetylcholine mediates the release of IL-8 in human bronchial epithelial cells by a NFkB/ERK-dependent mechanism. Eur J Pharmacol (2008) 582(1-3):145–53. 10.1016/j.ejphar.2007.12.029 18242599

[317] Carmona-Rivera C Purmalek MM Moore E Waldman M Walter PJ Garraffo HM. A role for muscarinic receptors in neutrophil extracellular trap formation and levamisole-induced autoimmunity. JCI Insight (2017) 2(3):e89780. 10.1172/jci.insight.89780 28194438 PMC5291726

[318] Kistemaker LE van Os RP Dethmers-Ausema A Bos IS Hylkema MN van den Berge M. Muscarinic M3 receptors on structural cells regulate cigarette smoke-induced neutrophilic airway inflammation in mice. Am J Physiol Lung Cell Mol Physiol (2015) 308(1):L96–103. 10.1152/ajplung.00259.2014 25381025 PMC4315453

[319] Profita M Bonanno A Montalbano AM Albano GD Riccobono L Siena L. beta(2) long-acting and anticholinergic drugs control TGF-beta1-mediated neutrophilic inflammation in COPD. Biochim Biophys Acta (2012) 1822(7):1079–89. 10.1016/j.bbadis.2012.03.002 22440430

[320] Nie Z Scott GD Weis PD Itakura A Fryer AD Jacoby DB. Role of TNF-alpha in virus-induced airway hyperresponsiveness and neuronal M(2) muscarinic receptor dysfunction. Br J Pharmacol (2011) 164(2b):444–52. 10.1111/j.1476-5381.2011.01393.x PMC3188913 21457223

[321] Paul S Lal G. The Molecular Mechanism of Natural Killer Cells Function and Its Importance in Cancer Immunotherapy. Front Immunol (2017) 8:1124. 10.3389/fimmu.2017.01124 28955340 PMC5601256

[322] Jiang W Li D Han R Zhang C Jin WN Wood K. Acetylcholine-producing NK cells attenuate CNS inflammation via modulation of infiltrating monocytes/macrophages. Proc Natl Acad Sci USA (2017) 114:E–6202-E11. 10.1073/pnas.1705491114 PMC5544318 28696300

[323] Hao J Shi FD Abdelwahab M Shi SX Simard A Whiteaker P. Nicotinic receptor beta2 determines NK cell-dependent metastasis in a murine model of metastatic lung cancer. PloS One (2013) 8(2):e57495. 10.1371/journal.pone.0057495 23469004 PMC3585320

[324] Marshall-Gradisnik S Huth T Chacko A Johnston S Smith P Staines D. Natural killer cells and single nucleotide polymorphisms of specific ion channels and receptor genes in myalgic encephalomyelitis/chronic fatigue syndrome. Appl Clin Genet (2016) 9:39–47. 10.2147/TACG.S99405 27099524 PMC4821384

[325] Jiang JL Qiu YH Peng YP. [Effect of acetylcholine on the cytotoxicity of natural killer cells]. Zhongguo Ying Yong Sheng Li Xue Za Zhi (2005) 21(3):330–3. 21162212

[326] Verbout NG Jacoby DB Gleich GJ Fryer AD. Atropine-enhanced, antigen challenge-induced airway hyperreactivity in guinea pigs is mediated by eosinophils and nerve growth factor. Am J Physiol Lung Cell Mol Physiol (2009) 297(2):L228–37. 10.1152/ajplung.90540.2008 PMC2742791 19447892

[327] Browning KN Travagli RA. Central nervous system control of gastrointestinal motility and secretion and modulation of gastrointestinal functions. Compr Physiol (2014) 4(4):1339–68. 10.1002/cphy.c130055 PMC4858318 25428846

[328] Tracey KJ. Reflex control of immunity. Nat Rev Immunol (2009) 9(6):418–28. 10.1038/nri2566 PMC4535331 19461672

[329] Stein KS McFarlane IC Goldberg N Ginzler EM. Heart rate variability in patients with systemic lupus erythematosus. Lupus (1996) 5(1):44–8. 10.1177/096120339600500109 8646225

[330] Pontet J Contreras P Curbelo A Medina J Noveri S Bentancourt S. Heart rate variability as early marker of multiple organ dysfunction syndrome in septic patients. J Crit Care (2003) 18(3):156–63. 10.1016/j.jcrc.2003.08.005 14595568

[331] Reisner BS Straley SC. Yersinia pestis YopM: thrombin binding and overexpression. Infect Immun (1992) 60(12):5242–52. 10.1128/IAI.60.12.5242-5252.1992 PMC258303 1452357

[332] Lindgren S Stewenius J Sjölund K Lilja B Sundkvist G. Autonomic vagal nerve dysfunction in patients with ulcerative colitis. Scand J Gastroenterol (1993) 28(7):638–42. 10.3109/00365529309096103 8362220

[333] Zi S Li J Liu L Liu F. Cholinergic anti-inflammatory pathway and its role in treatment of sepsis. Zhong Nan Da Xue Xue Bao Yi Xue Ban (2020) 45(1):68–73. 10.11817/j.issn.1672-7347.2020.180651 32132300

[334] Huston JM. The vagus nerve and the inflammatory reflex: wandering on a new treatment paradigm for systemic inflammation and sepsis. Surg Infect (Larchmt) (2012) 13(4):187–93. 10.1089/sur.2012.126 22913335

[335] Koopman FA Chavan SS Miljko S Grazio S Sokolovic S Schuurman PR. Vagus nerve stimulation inhibits cytokine production and attenuates disease severity in rheumatoid arthritis. Proc Natl Acad Sci USA (2016) 113(29):8284–9. 10.1073/pnas.1605635113 PMC4961187 27382171

[336] Onuora S. Rheumatoid arthritis: Vagus nerve stimulation reduces RA severity in patients. Nat Rev Rheumatol (2016) 12(9):500. 10.1038/nrrheum.2016.126 27440426

[337] Bonaz B Sinniger V Pellissier S. Targeting the cholinergic anti-inflammatory pathway with vagus nerve stimulation in patients with Covid-19? Bioelectron Med (2020) 6:15. 10.1186/s42234-020-00051-7 32743022 PMC7387121

[338] Porzionato A Emmi A Barbon S Boscolo-Berto R Stecco C Stocco E. Sympathetic activation: a potential link between comorbidities and COVID-19. FEBS J (2020) 287(17):3681–8. 10.1111/febs.15481 PMC7405290 32779891

[339] Rosas-Ballina M. al. e. Splenic nerve is required for cholinergic antiinflammatory pathway control of TNF in endotoxemia. PNAS (2008) 105(31):11008–13. 10.1073/pnas.0803237105 PMC2504833 18669662

[340] Reardon C Duncan GS Brustle A Brenner D Tusche MW Olofsson PS. Lymphocyte-derived ACh regulates local innate but not adaptive immunity. Proc Natl Acad Sci USA (2013) 110(4):1410–5. 10.1073/pnas.1221655110 PMC3557089 23297238

[341] Benowitz NL Burbank AD. Cardiovascular toxicity of nicotine: Implications for electronic cigarette use. Trends Cardiovasc Med (2016) 26(6):515–23. 10.1016/j.tcm.2016.03.001 PMC4958544 27079891

[342] Shao XM López-Valdés HE Liang J Feldman JL. Inhaled nicotine equivalent to cigarette smoking disrupts systemic and uterine hemodynamics and induces cardiac arrhythmia in pregnant rats. Sci Rep (2017) 7(1):16974. 10.1038/s41598-017-17301-5 29209071 PMC5717237

[343] Haussmann HJ Fariss MW. Comprehensive review of epidemiological and animal studies on the potential carcinogenic effects of nicotine per se. Crit Rev Toxicol (2016) 46(8):701–34. 10.1080/10408444.2016.1182116 PMC5020336 27278157

[344] Bagdas D AlSharari SD Freitas K Tracy M Damaj MI. The role of alpha5 nicotinic acetylcholine receptors in mouse models of chronic inflammatory and neuropathic pain. Biochem Pharmacol (2015) 97(4):590–600. 10.1016/j.bcp.2015.04.013 25931144 PMC4600420

[345] Kitagawa H Takenouchi T Azuma R Wesnes KA Kramer WG Clody DE. Safety, pharmacokinetics, and effects on cognitive function of multiple doses of GTS-21 in healthy, male volunteers. Neuropsychopharmacology (2003) 28(3):542–51. 10.1038/sj.npp.1300028 12629535

[346] Rosas-Ballina M Goldstein RS Gallowitsch-Puerta M Yang L Valdés-Ferrer SI Patel NB. The selective alpha7 agonist GTS-21 attenuates cytokine production in human whole blood and human monocytes activated by ligands for TLR2, TLR3, TLR4, TLR9, and RAGE. Mol Med (2009) 15(7-8):195–202. 10.2119/molmed.2009.00039 19593403 PMC2707516

[347] Yue Y Liu R Cheng W Hu Y Li J Pan X. GTS-21 attenuates lipopolysaccharide-induced inflammatory cytokine production in vitro by modulating the Akt and NF-κB signaling pathway through the α7 nicotinic acetylcholine receptor. Int Immunopharmacol (2015) 29(2):504–12. 10.1016/j.intimp.2015.10.005 26490221

[348] Wu XJ Yan XT Yang XM Zhang Y Wang HY Luo H. GTS-21 ameliorates polymicrobial sepsis-induced hepatic injury by modulating autophagy through alpha7nAchRs in mice. Cytokine (2020) 128:155019. 10.1016/j.cyto.2020.155019 32018068

[349] Kox M Pompe JC Gordinou de Gouberville MC van der Hoeven JG Hoedemaekers CW Pickkers P. Effects of the alpha7 nicotinic acetylcholine receptor agonist GTS-21 on the innate immune response in humans. Shock (Augusta Ga) (2011) 36(1):5–11. 10.1097/SHK.0b013e3182168d56 21368716

[350] Ulloa L. The anti-inflammatory potential of selective cholinergic agonists. Shock (2011) 36(1):97–8. 10.1097/SHK.0b013e31821820d2 PMC3174083 21677557

[351] Douaoui S Djidjik R Boubakeur M Ghernaout M Touil-Boukoffa C Oumouna M. GTS-21, an α7nAChR agonist, suppressed the production of key inflammatory mediators by PBMCs that are elevated in COPD patients and associated with impaired lung function. Immunobiology (2020) 225(3):151950. 10.1016/j.imbio.2020.151950 32387130 PMC7194070

[352] Wu S Zhao H Luo H Xiao X Zhang H Li T. GTS-21, an α7-nicotinic acetylcholine receptor agonist, modulates Th1 differentiation in CD4. Exp Ther Med (2014) 8(2):557–62. 10.3892/etm.2014.1754 PMC4079428 25009619

[353] Shin SS Dixon CE. Alterations in Cholinergic Pathways and Therapeutic Strategies Targeting Cholinergic System after Traumatic Brain Injury. J Neurotrauma (2015) 32(19):1429–40. 10.1089/neu.2014.3445 PMC4842943 25646580

[354] Yang T Xiao T Sun Q Wang K. The current agonists and positive allosteric modulators of. Acta Pharm Sin B (2017) 7(6):611–22. 10.1016/j.apsb.2017.09.001 PMC5687317 29159020

[355] van Westerloo DJ Giebelen IA Florquin S Bruno MJ Larosa GJ Ulloa L. The vagus nerve and nicotinic receptors modulate experimental pancreatitis severity in mice. Gastroenterology (2006) 130(6):1822–30. 10.1053/j.gastro.2006.02.022 16697744

[356] Gault LM Ritchie CW Robieson WZ Pritchett Y Othman AA Lenz RA. A phase 2 randomized, controlled trial of the alpha7 agonist ABT-126 in mild-to-moderate Alzheimer’s dementia. Alzheimers Dement (N Y) (2015) 1(1):81–90. 10.1016/j.trci.2015.06.001 29854928 PMC5974973

[357] Haig GM Wang D Zhao J Othman AA Bain EE. Efficacy and Safety of the alpha7-Nicotinic Acetylcholine Receptor Agonist ABT-126 in the Treatment of Cognitive Impairment Associated With Schizophrenia: Results From a Phase 2b Randomized Controlled Study in Smokers. J Clin Psychiatry (2018) 79(3):16m11162. 10.4088/JCP.16m11162 28922590

[358] Bernik TR Friedman SG Ochani M DiRaimo R Ulloa L Yang H. Pharmacological stimulation of the cholinergic antiinflammatory pathway. J Exp Med (2002) 195(6):781–8. 10.1084/jem.20011714 PMC2193742 11901203

[359] Bianchi M Ulrich P Bloom O Meistrell M Zimmerman GA Schmidtmayerova H. An inhibitor of macrophage arginine transport and nitric oxide production (CNI-1493) prevents acute inflammation and endotoxin lethality. Mol Med (1995) 1(3):254–66. 10.1007/BF03401550 PMC2229913 8529104

[360] Borovikova LV Ivanova S Nardi D Zhang M Yang H Ombrellino M. Role of vagus nerve signaling in CNI-1493-mediated suppression of acute inflammation. Auton Neurosci (2000) 85(1-3):141–7. 10.1016/S1566-0702(00)00233-2 11189021

[361] Bach JP Mengel D Wahle T Kautz A Balzer-Geldsetzer M Al-Abed Y. The role of CNI-1493 in the function of primary microglia with respect to amyloid-β. J Alzheimers Dis (2011) 26(1):69–80. 10.3233/JAD-2011-110179 21593565

[362] Björk L Tracey KJ Ulrich P Bianchi M Cohen PS Akerlund K. Targeted suppression of cytokine production in monocytes but not in T lymphocytes by a tetravalent guanylhydrazone (CNI-1493). J Infect Dis (1997) 176(5):1303–12. 10.1086/514126 9359732

[363] Cohen PS Nakshatri H Dennis J Caragine T Bianchi M Cerami A. CNI-1493 inhibits monocyte/macrophage tumor necrosis factor by suppression of translation efficiency. Proc Natl Acad Sci USA (1996) 93(9):3967–71. 10.1073/pnas.93.9.3967 PMC39469 8632999

[364] Abdalla H Forslund T Schön T Stendahl O Sundqvist T. Effects of CNI-1493 on human granulocyte functions. Immunobiology (2006) 211(3):191–7. 10.1016/j.imbio.2005.09.006 16530086

[365] Zinser E Turza N Steinkasserer A. CNI-1493 mediated suppression of dendritic cell activation in vitro and in vivo. Immunobiology (2004) 209(1-2):89–97. 10.1016/j.imbio.2004.04.004 15481144

[366] Hommes D van den Blink B Plasse T Bartelsman J Xu C Macpherson B. Inhibition of stress-activated MAP kinases induces clinical improvement in moderate to severe Crohn’s disease. Gastroenterology (2002) 122(1):7–14. 10.1053/gast.2002.30770 11781274

[367] van Westerloo DJ Rauws EA Hommes D de Vos AF van der Poll T Powers BL. Pre-ERCP infusion of semapimod, a mitogen-activated protein kinases inhibitor, lowers post-ERCP hyperamylasemia but not pancreatitis incidence. Gastrointest Endosc (2008) 68(2):246–54. 10.1016/j.gie.2008.01.034 18455169

[368] Tsifetaki N Kitsos G Paschides CA Alamanos Y Eftaxias V Voulgari PV. Oral pilocarpine for the treatment of ocular symptoms in patients with Sjogren’s syndrome: a randomised 12 week controlled study. Ann Rheumatic Dis (2003) 62(12):1204–7. 10.1136/ard.2002.003889 PMC1754388 14644860

[369] Habek M. Immune and autonomic nervous system interactions in multiple sclerosis: clinical implications. Clin Auton Res (2019) 29(3):267–75. 10.1007/s10286-019-00605-z 30963343

[370] Nile C Falleni M Cirasola D Alghamdi A Anderson OF Delaney C. Repurposing Pilocarpine Hydrochloride for Treatment of Candida albicans Infections. mSphere (2019) 4(1):e00689–18. 10.1128/mSphere.00689-18 PMC6344604 30674648

[371] Minagi HO Ikai K Araie T Sakai M Sakai T. Benefits of long-term pilocarpine due to increased muscarinic acetylcholine receptor 3 in salivary glands. Biochem Biophys Res Commun (2018) 503(2):1098–102. 10.1016/j.bbrc.2018.06.125 29953856

[372] Marchi N Oby E Batra A Uva L De Curtis M Hernandez N. In vivo and in vitro effects of pilocarpine: relevance to ictogenesis. Epilepsia (2007) 48(10):1934–46. 10.1111/j.1528-1167.2007.01185.x PMC3900294 17645533

[373] Scorza FA Arida RM Naffah-Mazzacoratti M Scerni DA Calderazzo L Cavalheiro EA. The pilocarpine model of epilepsy: what have we learned? Acad Bras Cienc (2009) 81(3):345–65. 10.1590/s0001-37652009000300003 19722008

[374] Tomiita M Takei S Kuwada N Nonaka Y Saito K Shimojo N. Efficacy and safety of orally administered pilocarpine hydrochloride for patients with juvenile-onset Sjögren’s syndrome. Mod Rheumatol (2010) 20(5):486–90. 10.1007/s10165-010-0313-7 20517630

[375] Hampel H Mesulam MM Cuello AC Farlow MR Giacobini E Grossberg GT. The cholinergic system in the pathophysiology and treatment of Alzheimer’s disease. Brain (2018) 141(7):1917–33. 10.1093/brain/awy132 PMC6022632 29850777

[376] Saldanha C. Human Erythrocyte Acetylcholinesterase in Health and Disease. Molecules (2017) 22(9):1499. 10.3390/molecules22091499 PMC6151671 28885588

[377] Freitas Leal JK Adjobo-Hermans MJW Brock R Bosman G. Acetylcholinesterase provides new insights into red blood cell ageing in vivo and in vitro. Blood Transfus (2017) 15(3):232–8. 10.2450/2017.0370-16 PMC5448829 28518050

[378] Das UN. Acetylcholinesterase and butyrylcholinesterase as possible markers of low-grade systemic inflammation. Med Sci Monit (2007) 13(12):RA214–21. 18049445

[379] Pohanka M. Inhibitors of acetylcholinesterase and butyrylcholinesterase meet immunity. Int J Mol Sci (2014) 15(6):9809–25. 10.3390/ijms15069809 PMC4100123 24893223

[380] Song P Spindel ER. Basic and clinical aspects of non-neuronal acetylcholine: expression of non-neuronal acetylcholine in lung cancer provides a new target for cancer therapy. J Pharmacol Sci (2008) 106(2):180–5. 10.1254/jphs.fm0070091 18285655

[381] Predescu DV Cretoiu SM Cretoiu D Pavelescu LA Suciu N Radu BM. G Protein-Coupled Receptors (GPCRs)-Mediated Calcium Signaling in Ovarian Cancer: Focus on GPCRs activated by Neurotransmitters and Inflammation-Associated Molecules. Int J Mol Sci (2019) 20(22):5568. 10.3390/ijms20225568 PMC6888001 31703453

[382] Buels KS Fryer AD. Muscarinic receptor antagonists: effects on pulmonary function. Handb Exp Pharmacol (2012) 208):317–41. 10.1007/978-3-642-23274-9_14 PMC4104281 22222705

[383] Tsuji H Okamoto K Matsuzaka Y Iizuka H Tamiya G Inoko H. SLURP-2, a novel member of the human Ly-6 superfamily that is up-regulated in psoriasis vulgaris. Genomics (2003) 81(1):26–33. 10.1016/s0888-7543(02)00025-3 12573258

[384] Baroni A Buommino E Ruocco E Petrazzuolo M De Filippis A Satriano RA. Captopril modulates acetylcholinesterase in human keratinocytes. Arch Dermatol Res (2011) 303(7):491–7. 10.1007/s00403-011-1124-1 21286734

[385] Yang WL Frucht H. Cholinergic receptor up-regulates COX-2 expression and prostaglandin E(2) production in colon cancer cells. Carcinogenesis (2000) 21(10):1789–93. 10.1093/carcin/21.10.1789 11023534

[386] Samuel I Zaheer S Fisher RA Zaheer A. Cholinergic receptor induction and JNK activation in acute pancreatitis. Am J Surg (2003) 186(5):569–74. 10.1016/j.amjsurg.2003.07.016 14599627

[387] Bacher I Wu B Shytle DR George TP. Mecamylamine - a nicotinic acetylcholine receptor antagonist with potential for the treatment of neuropsychiatric disorders. Expert Opin Pharmacother (2009) 10(16):2709–21. 10.1517/14656560903329102 19874251

[388] Bai X Stitzel JA Bai A Zambrano CA Phillips M Marrack P. Nicotine Impairs Macrophage Control of Mycobacterium tuberculosis. Am J Respir Cell Mol Biol (2017) 57(3):324–33. 10.1165/rcmb.2016-0270OC PMC5625222 28398760

[389] Brady WJ Swart G DeBehnke DJ Ma OJ Aufderheide TP. The efficacy of atropine in the treatment of hemodynamically unstable bradycardia and atrioventricular block: prehospital and emergency department considerations. Resuscitation (1999) 41(1):47–55. 10.1016/s0300-9572(99)00032-5 10459592

[390] Qiu YH Peng YP Jiang JL Wang JJ. Effect of acetylcholine on in vitro IL-2 production and NK cell cytotoxicity of rats. Lymphology (2004) 37(1):31–8. 15109075

[391] Freier S Eran M Faber J. Effect of cholecystokinin and of its antagonist, of atropine, and of food on the release of immunoglobulin A and immunoglobulin G specific antibodies in the rat intestine. Gastroenterology (1987) 93(6):1242–6. 10.1016/0016-5085(87)90251-4 3678742

[392] Juneja M Baidoo L Schwartz MB Barrie A Regueiro M Dunn M. Geriatric inflammatory bowel disease: phenotypic presentation, treatment patterns, nutritional status, outcomes, and comorbidity. Dig Dis Sci (2012) 57(9):2408–15. 10.1007/s10620-012-2083-x 22359191

[393] Gold R Hohlfeld R Toyka KV. Progress in the treatment of myasthenia gravis. Ther Adv Neurol Disord (2008) 1(2):36–51. 10.1177/1756285608093888 21180568 PMC3002545

[394] Galvis V Tello A Parra MM Merayo-Lloves J Larrea J Julian Rodriguez C. Topical Atropine in the Control of Myopia. Med Hypothesis Discovery Innov Ophthalmol (2016) 5(3):78–88. PMC5347209 28293653

[395] Page JG Dirnberger GM. Treatment of the irritable bowel syndrome with Bentyl (dicyclomine hydrochloride). J Clin Gastroenterol (1981) 3(2):153–6. 10.1097/00004836-198106000-00009 7016973

[396] Nee J Zakari M Lembo AJ. Novel Therapies in IBS-D Treatment. Curr Treat Options Gastroenterol (2015) 13(4):432–40. 10.1007/s11938-015-0068-5 26432092

[397] Ali A Jadhav A Jangid P Patil R Shelar A Karuppayil SM. The human muscarinic acetylcholine receptor antagonist, Dicyclomine targets signal transduction genes and inhibits the virulence factors in the human pathogen, Candida albicans. J Antibiot (Tokyo) (2018) 71(4):456–66. 10.1038/s41429-017-0013-z 29348527

[398] Karak P Kumar KA Mazumdar K Mookerjee M Dastidar SG. Antibacterial potential of an antispasmodic drug dicyclomine hydrochloride. Indian J Med Res (2003) 118:192–6. 14723484

[399] Talebi M Minai-Tehrani D Fazilati M Minai-Tehrani A. Inhibitory action of dicyclomine on lipase activity, kinetics and molecular study. Int J Biol Macromol (2018) 107(Pt B):2422–8. 10.1016/j.ijbiomac.2017.10.123 29055706

[400] Matera MG Rinaldi B Berardo C Rinaldi M Cazzola M. A review of the pharmacokinetics of M3 muscarinic receptor antagonists used for the treatment of asthma. Expert Opin Drug Metab Toxicol (2020) 16(2):143–8. 10.1080/17425255.2020.1716730 31958237

[401] Ohta S Oda N Yokoe T Tanaka A Yamamoto Y Watanabe Y. Effect of tiotropium bromide on airway inflammation and remodelling in a mouse model of asthma. Clin Exp Allergy (2010) 40(8):1266–75. 10.1111/j.1365-2222.2010.03478.x 20337647

[402] Buhling F Lieder N Kuhlmann UC Waldburg N Welte T. Tiotropium suppresses acetylcholine-induced release of chemotactic mediators in vitro. Respir Med (2007) 101(11):2386–94. 10.1016/j.rmed.2007.06.009 17761412

[403] Vacca G Randerath WJ Gillissen A. Inhibition of granulocyte migration by tiotropium bromide. Respir Res (2011) 12:24. 10.1186/1465-9921-12-24 21352583 PMC3051905

[404] Sato E Koyama S Okubo Y Kubo K Sekiguchi M. Acetylcholine stimulates alveolar macrophages to release inflammatory cell chemotactic activity. Am J Physiol (1998) 274(6 Pt 1):L970–9. 10.1152/ajplung.1998.274.6.L970 9609736

[405] Dantzer R. Neuroimmune Interactions: From the Brain to the Immune System and Vice Versa. Physiol Rev (2018) 98(1):477–504. 10.1152/physrev.00039.2016 29351513 PMC5866360

[406] Reardon C Murray K Lomax AE. Neuroimmune Communication in Health and Disease. Physiol Rev (2018) 98(4):2287–316. 10.1152/physrev.00035.2017 PMC6170975 30109819

[407] Murray K Barboza M Rude KM Brust-Mascher I Reardon C. Functional circuitry of neuro-immune communication in the mesenteric lymph node and spleen. Brain Behav Immun (2019) 82:214–23. 10.1016/j.bbi.2019.08.188 PMC6800652 31445965

[408] Bradley SJ Molloy C Valuskova P Dwomoh L Scarpa M Rossi M. Biased M1-muscarinic-receptor-mutant mice inform the design of next-generation drugs. Nat Chem Biol (2020) 16(3):240–9. 10.1038/s41589-019-0453-9 PMC7616160 32080630

